# Engineering Metal‐Organic‐Framework‐Based STING Nanoagonists for PROTAC‐Enhanced Cancer Chemo‐Metalloimmunotherapy

**DOI:** 10.1002/advs.202515006

**Published:** 2025-10-13

**Authors:** Zhenzhen Chen, Zhe Feng, Siyuan Wang, Jingjing Zhang

**Affiliations:** ^1^ State Key Laboratory of Analytical Chemistry for Life Science School of Chemistry and Chemical Engineering Chemistry and Biomedicine Innovation Center (ChemBIC) Nanjing University Nanjing 210023 China

**Keywords:** cancer therapy, chemotherapy, metalloimmunotherapy, PROTAC, STING activation

## Abstract

Chemo‐metalloimmunotherapy is emerging as a promising strategy for cancer treatment by integrating chemotherapy‐induced immunogenicity with metal ion‐mediated immune activation. However, its efficacy is hampered by chemoresistance and immune escape driven by PD‐L1 upregulation. Here, a multifunctional manganese‐based metal‐organic framework nanoplatform (Mn‐CDDP‐dBET6@CM) is reported that integrates metalloimmunotherapy, chemotherapy, and Proteolysis‐targeting chimera (PROTAC) ‐mediated epigenetic modulation for enhanced cancer treatment. This system co‐delivers Mn^2+^ to activate the stimulator of interferon genes (STING) pathway, cisplatin (CDDP) to induce nucleus DNA damage, and the bromodomain‐containing protein 4 (BRD4) ‐targeting PROTAC dBET6 to promote mitochondrial DNA release and suppress PD‐L1‐mediated immune evasion. Coated with tumor cell membranes for homologous targeting and immune evasion, Mn‐CDDP‐dBET6@CM effectively induces cellular senescence, robust innate and adaptive immune activation, and tumor microenvironment remodeling. In vitro and in vivo studies demonstrate potent tumor growth inhibition, enhance dendritic cell maturation, and increase cytotoxic T cell infiltration. This nanoplatform offers a promising strategy to overcome chemoresistance and immunosuppression, providing a versatile approach for next‐generation chemo‐metalloimmunotherapy.

## Graphical abstract

Schematic overview of PROTAC‐enhanced chemo‐metalloimmunotherapy, integrating senescence induction, multi‐modal STING pathway activation, PD‐L1 blockade, and immune amplification via coordinated PROTACs, metal ions, and chemotherapy to elicit robust antitumor immunity and circumvent immune evasion.

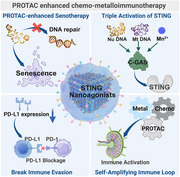

## 1 Introduction

Recent advances in immunology have highlighted the critical role of metal ions in modulating immune responses, fostering the emerging field of metalloimmunotherapy.^[^ ^1^, ^2^ ^]^ Metal ions such as Mn^2+^, Ca^2+^, and Zn^2+^ exhibit diverse immunomodulatory effects, providing promising strategies to enhance antitumor immunity.^[^ ^3^, ^4^, ^5^ ^]^ Among them, Mn^2+^ uniquely activates the cyclic GMP‐AMP synthase–stimulator of interferon genes (cGAS‐STING) pathway, a central axis in innate immune sensing, while promoting dendritic cell (DC) maturation and type I interferon production.^[^ ^6^, ^7^ ^]^ This positions Mn^2+^ as a leading candidate in metalloimmunotherapy development.^[^ ^8^ ^]^ Despite the exciting potential, the clinical translation of Mn^2+^‐based immunotherapy faces significant challenges.^[^ ^9^, ^10^ ^]^ Free Mn^2+^ ions exhibit rapid systemic clearance, nonspecific biodistribution, and risk of off‐target inflammation and neurotoxicity at high doses, limiting their therapeutic window.^[^ ^11^ ^]^ Moreover, efficient delivery and sustained activation of the cGAS‐STING pathway within the immunosuppressive tumor microenvironment (TME) remain problematic, as tumor heterogeneity and physical barriers reduce metal ion accumulation and immunostimulatory efficacy.^[^ ^12^ ^]^ These obstacles highlight the urgent need for advanced nanoplatforms enabling tumor‐targeted, controlled release of metal ions to maximize immunotherapeutic outcomes while minimizing systemic toxicity.^[^ ^13^ ^]^

**Figure 1:**
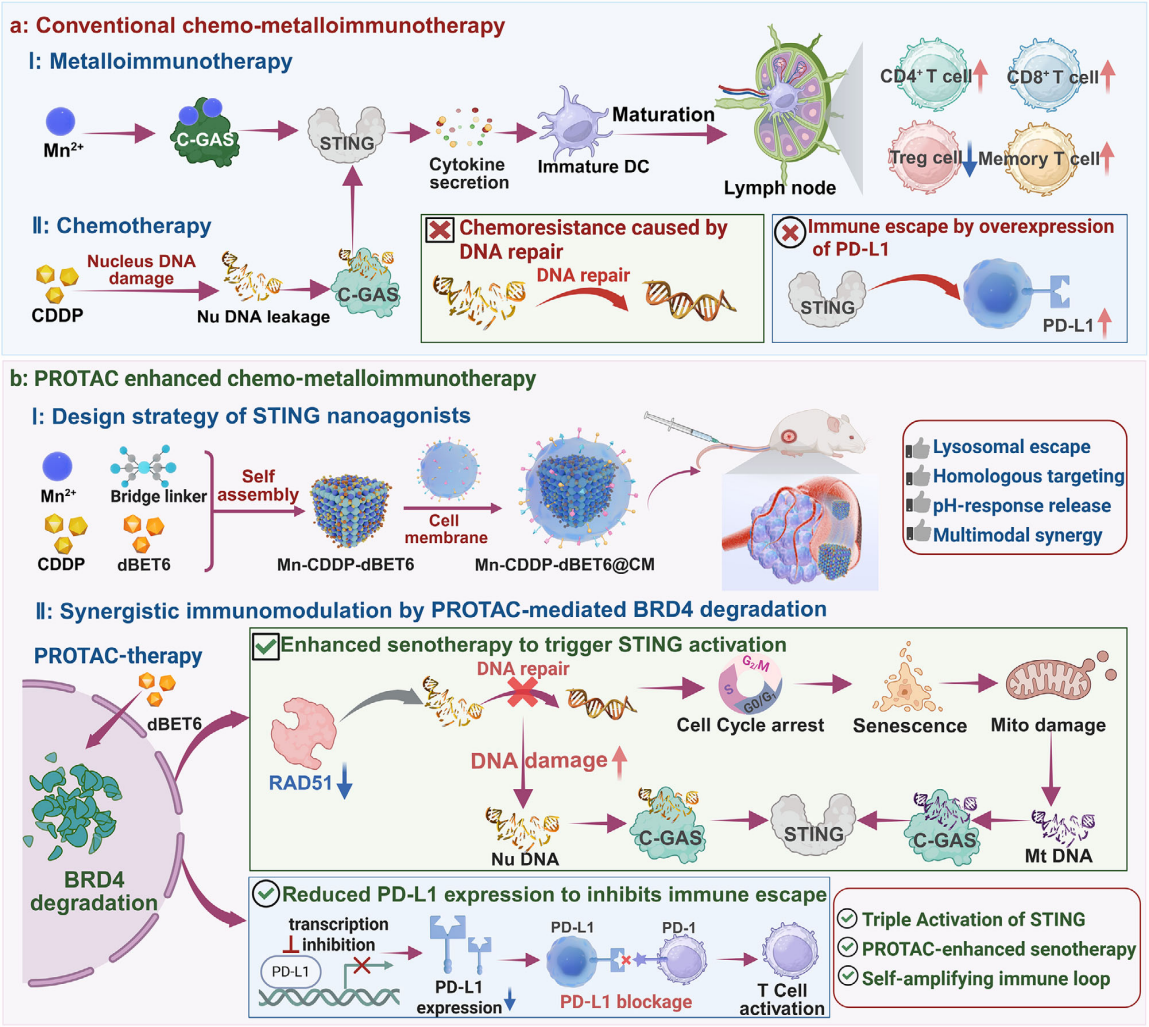
a) Conventional chemo‐metalloimmunotherapy is limited by chemoresistance caused by DNA repair and immune escape mediated by PD‐L1 overexpression. b) Our PROTAC‐enhanced chemo‐metalloimmunotherapy strategy, employing a manganese‐based MOF for co‐delivery of CDDP and a BRD4‐targeting PROTAC, drives triple STING activation, senescence‐mediated immune amplification, and PD‐L1 suppression, thereby overcoming chemoresistance and immune evasion through synergistic metallo‐immunomodulation and self‐sustaining innate immune stimulation. (Certain schematic elements in this figure were created using BioRender.com and are used under academic license).

Endogenous cGAS‐STING activation can also be triggered by cytoplasmic double‐stranded DNA (dsDNA) released from tumor cells^[^ ^14^ ^]^ following chemotherapy‐induced DNA damage.^[^ ^15^ ^]^ Agents like cisplatin (CDDP) not only cause direct cytotoxicity^[^ ^16^, ^17^ ^]^ but also enhance innate immune activation via ds DNA‐mediated STING stimulation.^[^ ^18^, ^19^ ^]^ Synergistic antitumor effects have been observed in conventional Chemo‐metalloimmunotherapy when combining Mn^2+^ with CDDP, leveraging both metalloimmunomodulation and immunogenic cell death (**Figure** **1**a).^[^ ^20^ ^]^ However, two major limitations impede clinical success, first, tumor resistance to CDDP arises primarily through enhanced DNA repair pathways, diminishing therapeutic efficacy;^[^ ^21^, ^22^ ^]^ second, prolonged STING activation paradoxically upregulates immune checkpoint molecules such as programmed death‐ligand 1 (PD‐L1). This effect is largely mediated by the sustained release of proinflammatory cytokines, including type I interferons and TNF‐α, which drive PD‐L1 transcription in both tumor and immune cells. As a consequence, tumor immune evasion is facilitated and cytotoxic T cell responses are suppressed (Figure 1a).^[^ ^23^, ^24^, ^25^ ^]^ Hence, innovative approaches are required to sustain and amplify STING signaling while concurrently overcoming chemoresistance and immunosuppressive feedback.

Proteolysis‐targeting chimeras (PROTACs) represent a transformative modality for selective and catalytic degradation of intracellular proteins by harnessing the ubiquitin‐proteasome system.^[^ ^26^ ^]^ Unlike conventional inhibitors that reversibly block protein function, PROTACs induce irreversible target degradation at low doses, mitigating off‐target effects and resistance development.^[^ ^27^ ^]^ In cancer therapy, PROTACs targeting epigenetic regulators such as bromodomain‐containing protein 4 (BRD4) have shown remarkable potential.^[^ ^28^, ^29^ ^]^ BRD4 functions as a chromatin reader orchestrating transcriptional programs involved in DNA damage repair, cell cycle progression, inflammatory signaling, and immune checkpoint expression.^[^ ^30^, ^31^, ^32^ ^]^ Targeted BRD4 degradation via PROTACs like dBET6 thus offers dual benefits by enhancing DNA damage‐induced tumor immunogenicity^[^ ^33^ ^]^ and suppressing PD‐L1‐mediated immune escape.^[^ ^34^, ^35^, ^36^, ^37^ ^]^

Building upon these insights, we herein present the rational design of a multifunctional nanoplatform, Mn‐CDDP‐dBET6@CM, that integrates metalloimmunotherapy with PROTAC‐assisted chemotherapy for synergistic cancer treatment (Figure 1b). The system co‐encapsulates CDDP and the BRD4‐targeting PROTAC dBET6 within a manganese‐based metal‐organic framework (Mn‐MOF), cloaked by homologous tumor cell membranes to achieve tumor‐specific targeting.^[^ ^38^ ^]^ This engineered nanoplatform is designed to simultaneously, (i) activate STING signaling via sustained Mn^2+^ release within the acidic TME;^[^ ^39^, ^40^ ^]^ (ii) induce nucleus DNA (Nu DNA) damage through controlled delivery of CDDP, with enhanced efficacy due to suppressed DNA repair via BRD4 degradation; and (iii) promote mitochondrial and nucleus ds DNA leakage triggered by BRD4 degradation‐induced cellular senescence and mitochondrial dysfunction.^[^ ^41^, ^42^ ^]^ Importantly, the PROTAC‐mediated BRD4 degradation also effectively downregulates PD‐L1 expression, thereby alleviating immune checkpoint‐mediated immunosuppression and preventing adaptive immune resistance. Mechanistic investigations reveal that Mn‐CDDP‐dBET6@CM not only induces robust nucleus γ‐H2AX‐marked DNA damage and cell cycle arrest but also promotes mitochondrial depolarization and mitochondrial DNA (Mt DNA) release, amplifying cytosolic DNA sensing via the cGAS‐STING axis.^[^ ^43^ ^]^ This multi‐source cytoplasmic DNA accumulation synergizes with Mn^2+^ stimulation to enhance STING pathway phosphorylation of TBK1 and IRF3, driving potent type I interferon responses.^[^ ^44^ ^]^ Consequent dendritic cell maturation and effector T cell activation foster a durable adaptive immune response, while transcriptomic analyses highlight broad gene expression changes encompassing DNA damage response, cellular senescence, and immune activation pathways.

Furthermore, tumor cell membrane cloaking confers homologous targeting, significantly enhancing cellular uptake, tumor accumulation, and immune evasion in vivo, leading to superior tumor growth inhibition without systemic toxicity. Importantly, the platform remodels the tumor immune microenvironment by enriching CD8⁺ and CD4⁺ T cell infiltration, reducing regulatory T (Treg) cell populations, and establishing immunological memory, thereby overcoming key limitations of traditional chemo‐metalloimmunotherapy. Collectively, this study demonstrates a closed‐loop, self‐amplifying nanoplatform that leverages PROTAC‐enabled epigenetic modulation to enhance metal ion‐based immunotherapy, chemotherapy, and senescence induction synergistically.

## 2 Results and Discussion

### 2.1 Synthesis and Characterization of Mn‐CDDP‐dBET6@CM

**Figure 2:**
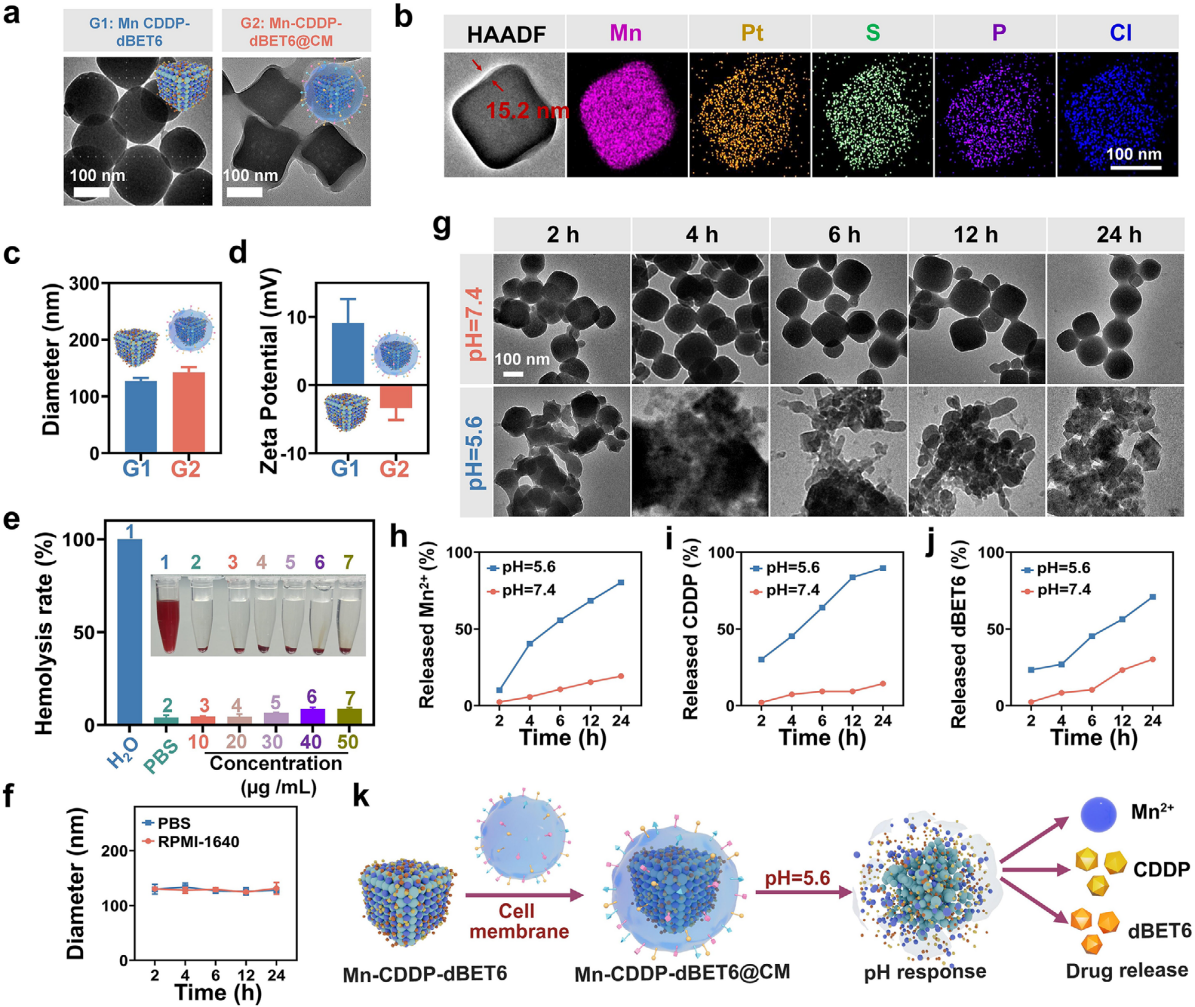
Preparation and characterization of Mn‐CDDP‐dBET6@CM. a) TEM images of Mn‐CDDP‐dBET6 and Mn‐CDDP‐dBET6@CM. b) HAADF‐STEM image and elemental mapping of Mn‐CDDP‐dBET6@CM. c) DLS and d) surface potential results of Mn‐CDDP‐dBET6 and Mn‐CDDP‐dBET6@CM. e) Hemolysis assay of Mn‐CDDP‐dBET6@CM at various concentrations; UV–vis spectra of supernatants and hemolysis percentage. f) Hydrodynamic particle size changes of Mn‐CDDP‐dBET6@CM during incubation in PBS buffer and RPMI‐1640 medium. g) Time‐dependent TEM images of Mn‐CDDP‐dBET6 under different pH conditions (pH 7.4 and 5.6). h–j) In vitro release profiles of Mn^2+^ (h), CDDP (i), and dBET6 (j) under pH 7.4 and 5.6. k) Schematic diagram illustrating the synthesis of Mn‐CDDP‐dBET6@CM and its pH‐responsive drug release property. Data presented as mean ± S.D. (n = 3). *P < 0.05, **P < 0.01, ***P < 0.001, ****P < 0.0001.

To achieve precise tumor targeting and multimodal therapeutic synergy, we developed a manganese‐based nanoplatform, Mn‐CDDP‐dBET6@CM, by co‐encapsulating CDDP and dBET6 into a Mn‐based metal‐organic framework^[^ ^45^ ^]^ (Mn‐CDDP‐dBET6), followed by cloaking with homologous 4T1 tumor cell membranes (CM). The successful construction and structural integrity of the nanoplatform were thoroughly validated. Powder X‐ray diffraction (XRD) patterns confirmed that the crystalline structure of Mn‐MOF remained intact after drug loading^[^ ^46^ ^]^ (Figure S1, Supporting Information). Transmission electron microscopy (TEM) revealed a well‐defined cubic morphology of Mn‐CDDP‐dBET6, whereas the CM‐coated formulation exhibited a distinct core‐shell architecture (**Figure** **2**a), indicating successful membrane encapsulation. SDS‐PAGE analysis further demonstrated that membrane protein profiles of Mn‐CDDP‐dBET6@CM closely resembled those of native 4T1 cell membranes (Figure S2, Supporting Information), suggesting preservation of functional surface proteins relevant to immune evasion and homotypic targeting. High‐angle annular dark‐field scanning transmission electron microscopy (HAADF‐STEM) and elemental mapping confirmed the uniform distribution of Mn and Pt elements across the nanostructure (Figure 2b), supporting homogeneous drug incorporation and membrane coverage. Dynamic light scattering (DLS) analysis showed an increase in hydrodynamic diameter after membrane coating, accompanied by a shift in zeta potential from +9.11 ± 3.51 mV to −3.36 ± 1.76 mV (Figure 2c,d), consistent with successful surface functionalization by negatively charged cell membranes. The biocompatibility of Mn‐CDDP‐dBET6@CM was confirmed by hemolysis assays, which revealed hemolysis rates below 10% across a range of concentrations (Figure 2e; Figure S3, Supporting Information), indicating excellent hemocompatibility suitable for systemic administration. Additionally, the nanoplatform exhibited strong colloidal stability in both PBS and RPMI‐1640 media, with minimal changes in hydrodynamic size during incubation (Figure 2f). To evaluate pH responsiveness, we assessed both morphological and drug release profiles under physiological (pH 7.4) and tumor‐mimicking acidic (pH 5.6) conditions. Time‐dependent TEM imaging showed that the nanostructure remained stable at pH 7.4 but underwent substantial disassembly at pH 5.6 (Figure 2g), reflecting acid‐triggered destabilization in the TME. Correspondingly, drug release assays demonstrated accelerated release of Mn^2+^, CDDP, and dBET6 at pH 5.6 compared to pH 7.4, with cumulative release reaching approximately 80% within 24 h (Figure 2h–j). A schematic illustration of the construction strategy and pH‐responsive behavior is provided in Figure 2k, highlighting the design rationale for tumor‐selective delivery and controlled release. Collectively, these results confirm that Mn‐CDDP‐dBET6@CM exhibits excellent stability, biocompatibility, and pH‐responsive degradability in acidic TMEs, establishing a robust foundation for subsequent therapeutic investigations.

### 2.2 Cellular Uptake Capacity and Targeting Specificity of Mn‐CDDP‐dBET6@CM

**Figure 3:**
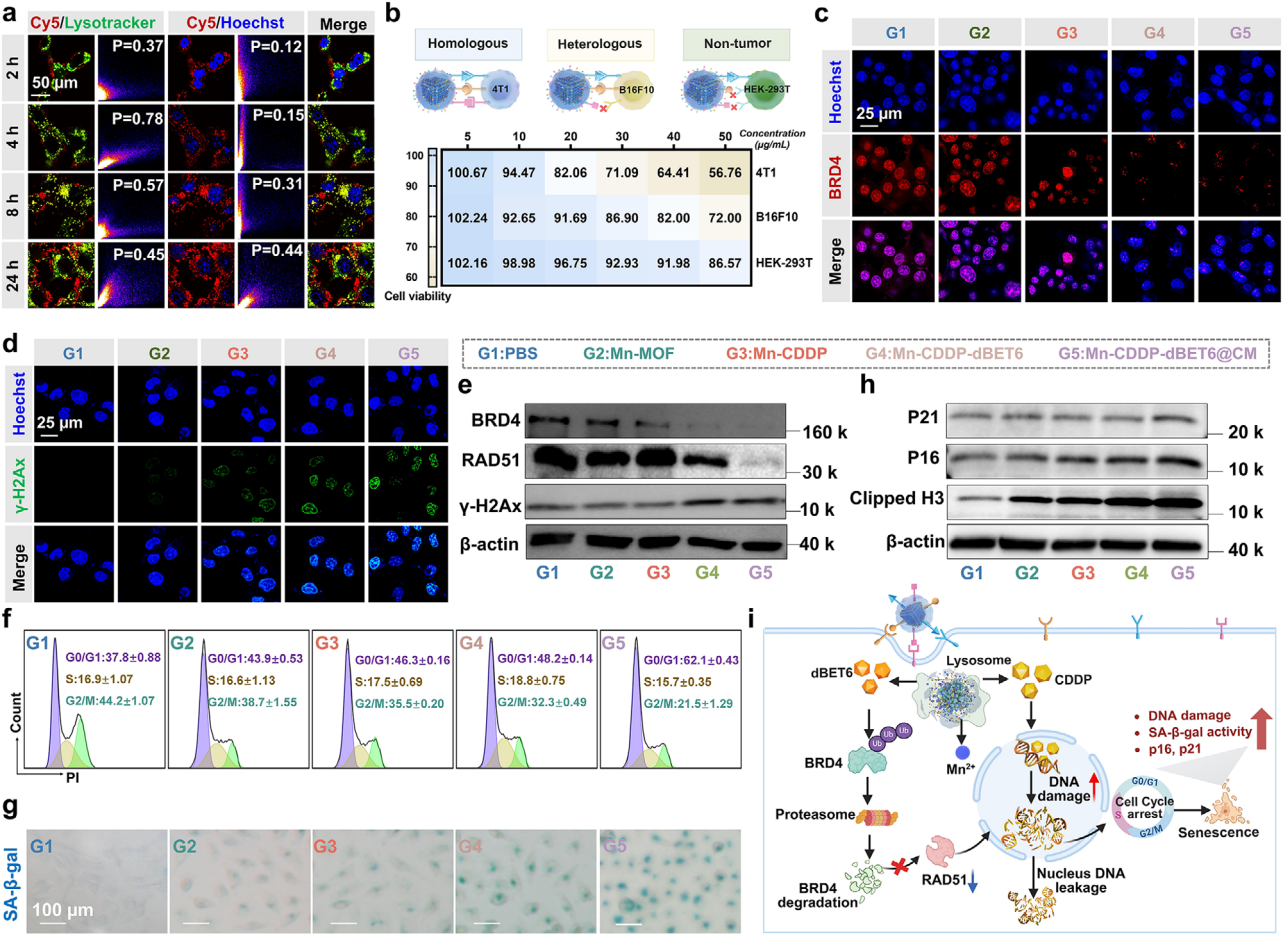
Mechanistic investigation of nucleus DNA damage and senescence induction by Mn‐CDDP‐dBET6@CM. a) Representative CLSM images of 4T1 cells incubated with Mn‐CDDP‐dBET6@4T1 CM for 2, 4, 8, and 24 hours. b) Cell viability assays showing Mn‐CDDP‐dBET6@CM displays significantly enhanced antiproliferative efficacy through homologous cell membrane‐targeting. c) Intracellular BRD4 (red) and d) γ‐H2Ax (green) in 4T1 cells after different treatments. e) Western blot analysis of BRD4, RAD51, and γ‐H2Ax protein expression in 4T1 cells after various treatments. f) Cell cycle analysis of 4T1 cells after different treatments, as determined by flow cytometry using propidium iodide staining. g) Cell senescence was assessed by SA‐β‐Gal staining under different treatments. h) Western blot analysis on the expression of p21, p16, and Clipped H3 expression after indicated treatments. i) Schematic diagram of Mn‐CDDP‐dBET6@CM inducing nucleus DNA damage and cellular senescence.

Following the successful construction and physicochemical characterization of Mn‐CDDP‐dBET6@CM, we next investigated its cellular uptake efficiency and tumor‐targeting specificity. Given the design rationale of homologous membrane cloaking for enhanced recognition, we sought to determine whether the presence of tumor‐derived cell membranes could promote selective internalization in homologous cancer cells. To assess this, Cyanine5 (cy5)‐labeled Mn‐CDDP‐dBET6 and its membrane‐coated formulation, Mn‐CDDP‐dBET6@CM, were incubated with 4T1 cells, and cellular uptake was analyzed via flow cytometry (Figure  S4a–d, Supporting Information). Both formulations exhibited time‐dependent internalization, but Mn‐CDDP‐dBET6@CM consistently demonstrated significantly higher uptake at all time points, suggesting that membrane camouflage facilitated improved interaction with the target cells. To further examine targeting selectivity, Mn‐CDDP‐dBET6@4T1 CM was incubated with three different cell types, homologous 4T1 breast cancer cells, heterologous B16F10 melanoma cells, and non‐malignant HEK‐293T cells. Confocal laser scanning microscopy (CLSM) revealed strong intracellular fluorescence in 4T1 cells within 2 h, which increased over time and was accompanied by clear evidence of lysosomal escape at 8 hours (**Figure** **3**a). The notably faster internalization in 4T1 cells was quantified by a high Pearson's colocalization coefficient with lysosomes of 0.78 at just 4 h, compared to 0.56 and 0.45 for B16F10 and HEK‐293T cells at 24 h, respectively, underscoring that homotypic targeting accelerates cellular entry. In contrast, B16F10 cells exhibited significantly weaker fluorescence signals, while HEK‐293T cells showed minimal uptake (Figures S5,S6, Supporting Information). These findings were quantitatively corroborated by flow cytometry (Figure S7, Supporting Information), which confirmed markedly higher mean fluorescence intensity (MFI) in 4T1 cells 2.08‐fold and 3.25‐fold higher than that in B16F10 and HEK‐293T cells at 24 h, respectively. The pronounced cellular selectivity observed in uptake was directly reflected in cytotoxicity outcomes. As shown in Figure 3b, Mn‐CDDP‐dBET6@4T1 CM exhibited a dose‐dependent cytostatic effect in 4T1 cells, while having substantially reduced cytotoxicity in B16F10 cells, and minimal toxicity in HEK‐293T cells. This selective toxicity was starkly evident at a concentration of 50 µg mL^−1^, where the viability of HEK‐293T, B16F10, and 4T1 cells was 86.57%, 72.00%, and 56.76%, respectively. These results suggest that the enhanced uptake and therapeutic efficacy in 4T1 cells can be attributed to a homotypic recognition mechanism, wherein membrane‐derived ligands on the nanoparticle surface selectively interact with receptors on the parental cancer cell type. This receptor‐ligand compatibility is likely attenuated in heterologous cancer cells and largely absent in normal cells, resulting in the observed differences in endocytosis and cytotoxic response.^[^ ^47^ ^]^ Altogether, these findings validate that tumor cell membrane camouflaging not only promotes efficient cellular uptake but also confers selective tumor targeting through a homologous recognition mechanism.

### 2.3 Mn‐CDDP‐dBET6@CM Achieves Enhanced Anti‐Tumor Effects by Inducing Nucleus DNA Damage and Cellular Senescence

Building on the confirmed homologous targeting and efficient intracellular delivery of Mn‐CDDP‐dBET6@CM, we next investigated its anti‐tumor efficacy and underlying mechanisms at the cellular level. Cytotoxicity of different group on 4T1 tumor cells was assessed by CCK‐8 assay. As shown in Figure S8 (Supporting Information), Mn‐CDDP‐dBET6@CM significantly inhibited 4T1 cell proliferation within 24 h. Notably, its cytotoxicity was stronger than that of the uncoated Mn‐CDDP‐dBET6 as well as any single‐drug treatment, indicating a synergistic therapeutic effect arising from the integration of chemo‐metalloimmunotherapy and PROTAC therapy. To further evaluate the mode of cell death, we performed Annexin V‐FITC/PI double staining and observed a marked increase late apoptotic populations following Mn‐CDDP‐dBET6@CM treatment (Figure S9a,b, Supporting Information). Consistently, live/dead cell staining showed prominent PI‐positive signals in treated cells, indicating widespread cell death (Figure S10, Supporting Information). These findings supported the potent cytotoxic activity of the nanoplatform.^[^ ^48^ ^]^ We then turned our attention to the mechanistic basis of this cytotoxicity, focusing on two central axes of the platform's design, BRD4 degradation and DNA damage. Immunofluorescence staining showed a clear reduction in BRD4 expression, accompanied by enhanced γ‐H2AX foci formation (Figure 3c,d). Western blot results further confirmed significant downregulation of BRD4 and the DNA repair protein RAD51, along with upregulation of γ‐H2AX^[^ ^49^ ^]^ (Figure 3e, uncropped blot image shown in Figure S11, Supporting Information), indicating that the nanoplatform not only induces genotoxic stress but also suppresses homologous recombination repair. As DNA damage commonly leads to cell cycle arrest,^[^ ^50^ ^]^ we next assessed the cell cycle distribution. Flow cytometry revealed an accumulation of cells in the G0/G1 phase following treatment, with the S phase largely unchanged and the G2/M phase showing an opposite trend to G0/G1 (Figure 3f; Figure S12, Supporting Information), consistent with checkpoint activation in response to unrepaired DNA damage. Prolonged cell cycle blockade often promotes a senescent phenotype, and indeed, SA‐β‐Gal staining revealed extensive cellular senescence in treated cells^[^ ^51^, ^52^ ^]^ (Figure 3g). Western blot further demonstrated increased expression of senescence markers p21 and p16, as well as Clipped H3, a histone variant associated with chromatin remodeling during senescence^[^ ^52^ ^]^ (Figure 3h, uncropped blot image shown in Figure  S13, Supporting Information). These results were collectively illustrated in the schematic shown in Figure 3i. Taken together, these findings confirm that Mn‐CDDP‐dBET6@CM induces potent anti‐tumor effects through a coordinated mechanism involving BRD4 degradation, platinum‐induced DNA damage, and cell cycle arrest‐driven senescence. Specifically, DNA damage was evidenced by γ‐H2AX accumulation, which led to upregulation of cell cycle inhibitors p21 and p16, ultimately resulting in increased SA‐β‐Gal staining and cellular senescence. This triple‐action strategy is tightly aligned with our therapeutic design, by co‐delivering a chemotherapeutic and a PROTAC within a Mn^2+^‐based immunomodulatory framework, the platform not only overcomes DNA repair‐associated resistance but also amplifies cellular stress responses, leading to irreversible growth inhibition through programmed senescence. This mechanistic synergy lays the foundation for subsequent immune activation and TME remodeling in vivo.

### 2.4 Mitochondrial Stress‐Induced mt DNA Release and BRD4‐Mediated PD‐L1 Downregulation

**Figure 4:**
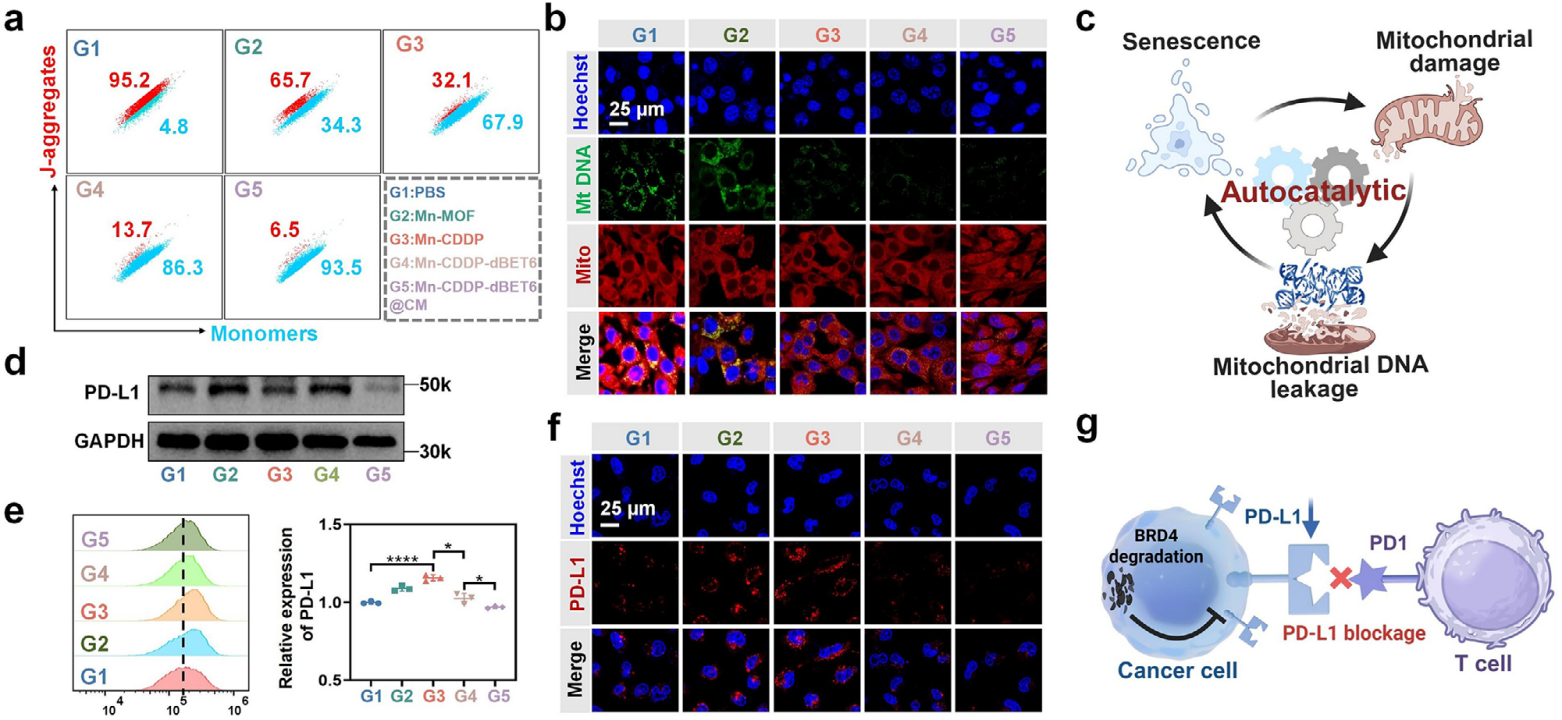
Analysis of mt DNA release during senescence and BRD4 degradation‐mediated modulation of PD‐L1 expression. a) Representative flow cytometry histograms of 4T1 cells treated with the mitochondrial probe JC‐1 after different treatments. b) Immunofluorescence images of mt DNA release in 4T1 cells. c) Schematic illustration depicting mt DNA release associated with cellular senescence. Western blot d), flow cytometry e), and immunofluorescence staining f) analysis of the expression of PD‐L1 in 4T1 cells after different treatments. g) Schematic representation of PROTAC‐induced BRD4 degradation enabling downregulation of PD‐L1 and suppression of immune evasion pathways. Data presented as mean ± S.D. (n = 3). *P < 0.05, **P < 0.01, ***P < 0.001, ****P < 0.0001.

Having confirmed that Mn‐CDDP‐dBET6@CM induces significant nu DNA damage and promotes cellular senescence, we further investigated whether these effects are accompanied by mitochondrial dysfunction and changes in immune regulatory markers. Mitochondrial stress is a well‐documented feature of senescent cells and is often associated with the release of mt DNA into the cytosol, which may contribute to innate immune activation.^[^ ^53^, ^54^ ^]^ To assess mitochondrial integrity, we performed JC‐1 staining to evaluate mitochondrial membrane potential. In untreated cells, JC‐1 forms aggregate within healthy mitochondria, exhibiting red fluorescence, while mitochondrial depolarization causes a shift toward green fluorescence due to monomeric JC‐1. Both flow cytometry and confocal laser scanning microscopy (CLSM) revealed a marked decrease in the red/green fluorescence ratio in Mn‐CDDP‐dBET6@CM‐treated 4T1 cells (**Figure** **4**a; Figures S14,S15, Supporting Information), indicating substantial loss of mitochondrial membrane potential.

To examine mt DNA release, we conducted immunofluorescence staining of mt DNA‐binding protein TFAM (Transcription Factor A, Mitochondrial).^[^ ^55^ ^]^ In control cells, TFAM exhibited a filamentous, network‐like distribution along intact mitochondria. In contrast, Mn‐CDDP‐dBET6@CM‐treated cells showed disrupted and punctate TFAM signals dispersed in the cytoplasm (Figure 4b), suggesting translocation of mt DNA due to mitochondrial stress. These findings indicate that Mn‐CDDP‐dBET6@CM induces dual genomic insults, nu DNA damage and mitochondrial dysfunction, collectively promoting cytoplasmic accumulation of both nu DNA and mt DNA. Senescent cells subsequently amplify the feedback loop via secreting pro‐inflammatory factors, which exacerbate mitochondrial instability and mt DNA release, forming a self‐reinforcing autocatalytic cascade. This integrated mechanism, schematically illustrated in Figure 4c, highlights a synergistic metalloimmunomodulatory strategy targeting both genomic and mitochondrial stress pathways.

Given that BRD4 plays a critical role not only in transcription and DNA repair but also in immune regulation‐particularly the transcriptional control of PD‐L1. We further explored whether BRD4 degradation by dBET6 modulates PD‐L1 expression. Western blot analysis revealed that Mn‐CDDP‐dBET6@CM significantly downregulated PD‐L1 expression in parallel with BRD4 depletion (Figure 4d, uncropped blot image shown in Figure S16, Supporting Information), suggesting that the PROTAC component effectively disrupts BRD4 mediated immunosuppressive signaling. Flow cytometry (Figure 4e) and immunofluorescence analysis (Figure 4f) corroborated these findings, showing consistent decreases in PD‐L1 protein levels. The mechanistic illustration in Figure 4g depicts this regulatory axis, in which BRD4 degradation suppresses PD‐L1 expression and potentially enhances the susceptibility of tumor cells to immune surveillance. These findings collectively demonstrate that Mn‐CDDP‐dBET6@CM induces mitochondrial dysfunction and mt DNA release as a downstream consequence of senescence, while concurrently suppressing PD‐L1 expression through BRD4 degradation. By coordinating mitochondrial stress‐associated immune activation and epigenetic checkpoint regulation, the nanoplatform provides a dual regulatory mechanism that both enhances innate immune signaling and reduces immune escape. This multifaceted immunomodulatory effect is not only consistent with the rational design of the nanoplatform, but also highlights its potential as a multifunctional strategy for chemo‐metalloimmunotherapy.

### 2.5 STING Pathway Activation and Immune Cell Stimulation by Mn‐CDDP‐dBET6@CM In Vitro

**Figure 5:**
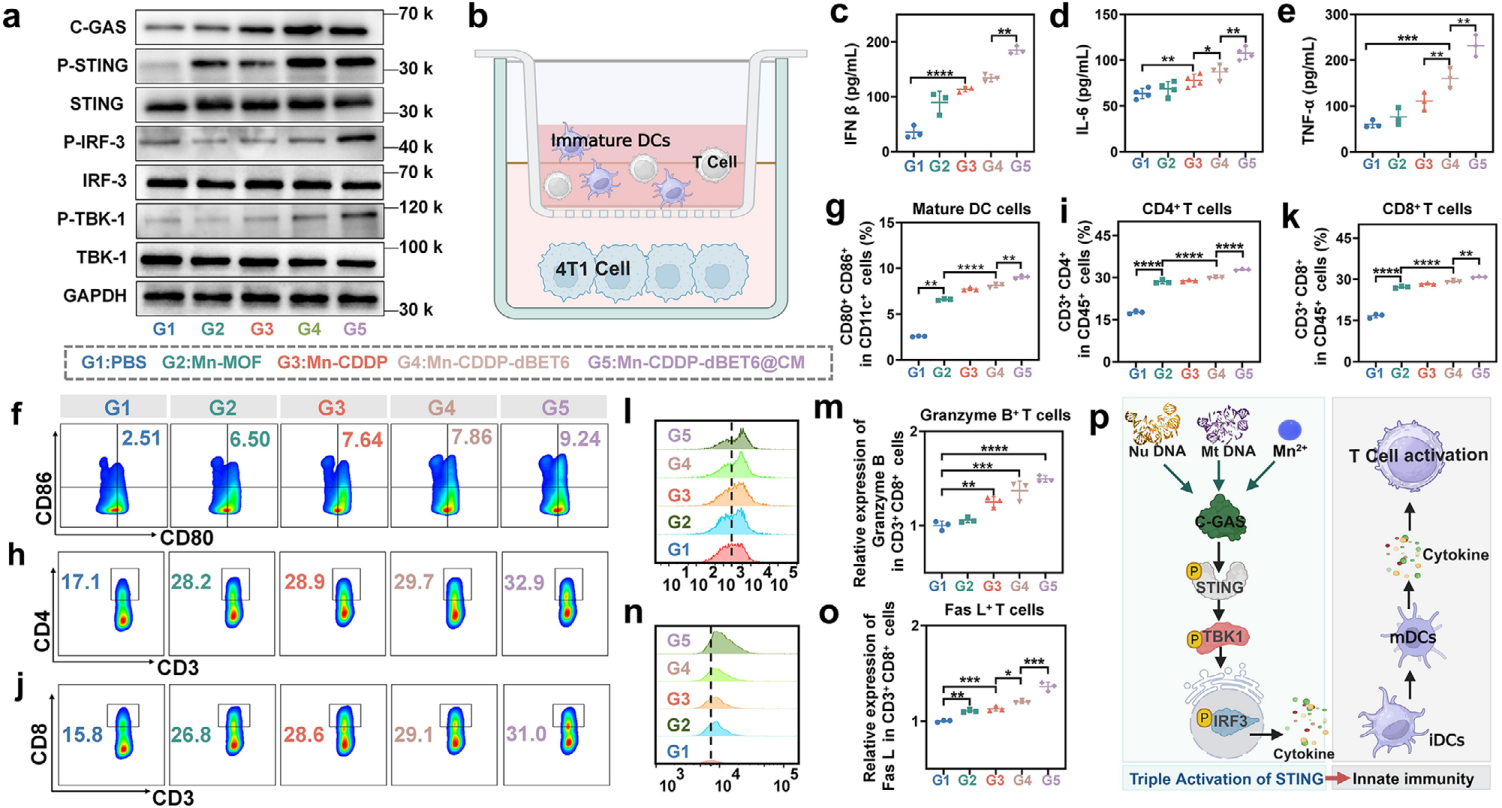
STING activation by Mn‐CDDP‐dBET6@CM in vitro. a) Protein expression of cGAS‐STING in 4T1 cells. b) The transwell system was utilized to explore the maturation of DCs induced by different treatments for tumor cells. The upper layer is DCs and T cells but the lower layer is 4T1 cells. c–e) Cytokine concentrations of IFN‐β (c), IL‐6 (d), and TNF‐α (e) in supernatants after indicated treatments. f, g) Flow cytometry analysis of BMDCs maturation induced by culture medium supernatant of tumor cells and the corresponding quantitative analysis. CD4^+^ T cells h), CD8^+^ T cells j) was induced by culture medium supernatant of tumor cells and the corresponding quantitative analysis i, k) pretreated with different treatments. l, m) Representative flow cytometry plots showing Granzyme B expression on CD8⁺ T cells and quantitative analysis of the MFI of Granzyme B on CD8⁺ T cells. n, o) Representative flow cytometry plots showing Fas ligand (Fas L) expression on CD8⁺ T cells and quantitative analysis of the MFI of Fas L on CD8⁺ T cells. p) Potential mechanism of cytoplasmic DNA accumulation and Mn^2+^ activating innate immunity. Immature dendritic cells, iDCs; mature dendritic cells, mDCs. Data presented as mean ± S.D. (n = 3). *P < 0.05, **P < 0.01, ***P < 0.001, ****P < 0.0001.

Following confirmation that Mn‐CDDP‐dBET6@CM induces DNA damage and cellular senescence, we further examined whether these stress responses could activate innate immune signaling in tumor cells. Given the role of the cGAS‐STING axis as a key DNA‐sensing pathway that bridges genotoxic stress and immune activation, we first evaluated its activation status in 4T1 cells. Western blot analysis revealed that treatment with Mn‐CDDP‐dBET6@CM significantly upregulated cGAS and the phosphorylation levels of STING, TBK1, and IRF3^[^ ^6^ ^]^ (**Figure** **5**a, uncropped blot image shown in Figure S17, Supporting Information), indicating robust activation of the STING signaling cascade at the tumor cell level. To assess whether this activation could be translated into functional stimulation of immune cells, we established a Trans well‐based in vitro co‐culture system to simulate the tumor‐immune interaction microenvironment (Figure 5b).^[^ ^56^ ^]^ In this system, 4T1 tumor cells subjected to different treatments were placed in the lower chamber, while bone marrow‐derived dendritic cells (BMDCs) and T cells were seeded in the upper chamber. Analysis of the culture supernatants revealed that the Mn‐CDDP‐dBET6@CM group induced significantly higher secretion of IFN‐β, IL‐6, and TNF‐α compared to single‐drug or untreated controls (Figure 5c–e), suggesting that treated tumor cells and BMDCs released potent immune‐stimulating signals. ELISA showed increased IL‐6 secretion after Mn‐CDDP‐dBET6@CM treatment. As IL‐6 is a well‐recognized component of the senescence‐associated secretory phenotype (SASP), its upregulation further supports that Mn‐CDDP‐dBET6@CM induces cellular senescence in addition to DNA damage and cell cycle arrest. Correspondingly, flow cytometry analysis showed that the tumor‐conditioned medium from the Mn‐CDDP‐dBET6@CM group markedly promoted the maturation of DCs, as indicated by increased expression of CD80 and CD86 (Figure 5f,g, gating strategy shown in Figure S18, Supporting Information), along with the activation and expansion of both CD4⁺ T cells and CD8⁺ T cells (Figure 5h–k, gating strategy shown in Figure S19, Supporting Information). To further investigate the cytotoxic potential of the activated CD8⁺ T cells, we examined the expression of Granzyme B, a pivotal cytolytic effector molecule, along with the Fas/Fas ligand (Fas L) pathway. Flow cytometry analysis revealed a substantial increase in Granzyme B expression in CD8⁺ T cells from the Mn‐CDDP‐dBET6@CM group (Figure 5l,m, gating strategy shown in Figure S20, Supporting Information). Furthermore, a 1.36‐fold upregulation in the MFI of Fas ligand (Fas L) was observed on these CD8⁺ T cells relative to controls (Figure 5n–o; gating strategy provided in Figure S21, Supporting Information). Concomitantly, Fas expression on tumor cells was markedly elevated by 2.10‐fold (Figure S22, Supporting Information). This coordinated enhancement of Granzyme B, along with the Fas L on effector T cells and its cognate Fas receptor on target cells, provides compelling evidence for the activation of dual cytotoxic mechanisms, underscoring a robust CD8⁺ T cell‐mediated immune response. These results collectively suggest that the nanoplatform not only activates innate immunity in tumor cells, but also promotes dendritic cell maturation, effectively triggering downstream adaptive immune responses including T cell activation and the acquisition of cytotoxic effector function. A schematic illustration (Figure 5p) summarizes the underlying mechanism, in which Mn‐CDDP‐dBET6@CM synergistically activates the STING pathway through cytoplasmic DNA accumulation and Mn^2+^ stimulation, leading to dendritic cell maturation and T cell activation. These findings provide compelling mechanistic evidence that Mn‐CDDP‐dBET6@CM serves as a potent immunomodulatory agent, and lay the groundwork for subsequent in vivo investigations of its therapeutic efficacy.

### 2.6 RNA‐Seq Analysis of Tumor Cells During Cellular Senescence or STING Activation

**Figure 6:**
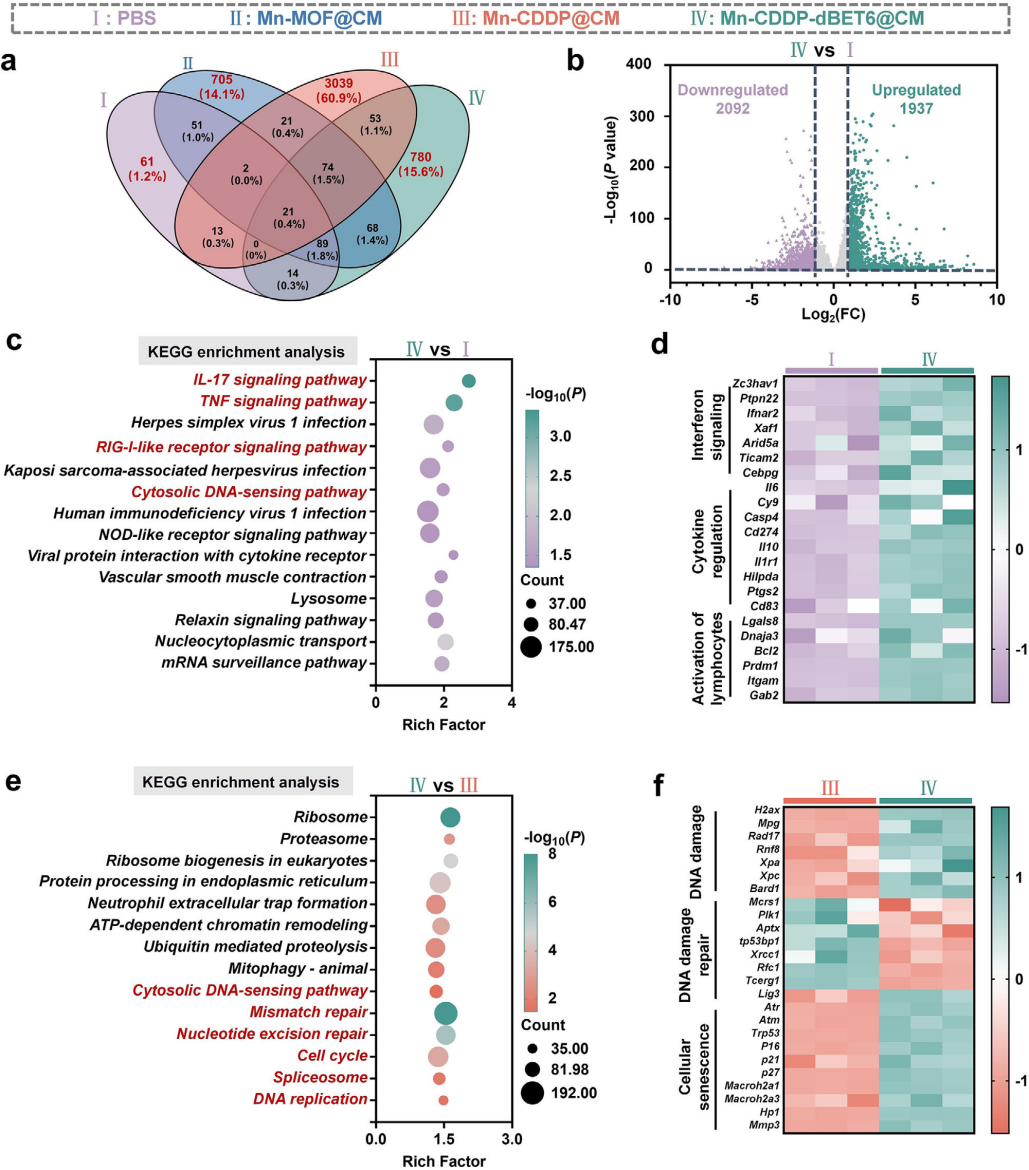
RNA‐seq analysis of 4T1 cells treated with different groups. a) Venn diagram showing the number of genes transcribed in each treatment group. b) Volcano plot showing differentially expressed genes (DEGs) between PBS and Mn‐CDDP‐dBET6@CM‐treated cells. c) KEGG pathway enrichment analysis of DEGs identified between PBS and Mn‐CDDP‐dBET6@CM groups. d) Heatmap showing the expression of genes related to innate immunity in Mn‐CDDP‐dBET6@CM‐treated cells compared to PBS. e) KEGG analysis of DEGs between Mn‐CDDP@CM‐treated cells and those treated with Mn‐CDDP‐dBET6@CM. f) Heatmap displaying the expression profiles of genes involved in the DNA‐sensing pathway between the two treatment groups.

To gain a comprehensive understanding of the molecular regulatory effects exerted by Mn‐CDDP‐dBET6@CM on tumor cells, we performed RNA sequencing analysis of 4T1 cells treated with PBS, Mn‐MOF@CM, Mn‐CDDP@CM, and Mn‐CDDP‐dBET6@CM. Venn diagram analysis revealed a core set of co‐expressed genes across all groups, reflecting the preservation of essential cellular functions (**Figure** **6**a). Notably, Mn‐CDDP‐dBET6@CM uniquely induced the expression of 780 specific genes, suggesting broad regulatory alterations triggered by the multifunctional nanoplatform. Volcano plot analysis further identified 4029 differentially expressed genes (DEGs) compared to the PBS control, including 1937 upregulated and 2092 downregulated genes (Figure 6b). Gene Ontology (GO) enrichment (Figure S23, Supporting Information) demonstrated that Mn‐CDDP‐dBET6@CM significantly influenced pathways related to DNA damage response, cellular senescence, and immune activation, aligning with our prior mechanistic observations. Kyoto Encyclopedia of Genes and Genomes (KEGG) pathway analysis (Figure 6c) further confirmed enrichment in immune‐relevant signaling cascades, including the IL‐17, TNF, RIG‐I‐like receptor, cytosolic DNA‐sensing, and NOD‐like receptor pathways‐many of which are central to antiviral and antitumor immunity. These transcriptomic shifts support the notion that Mn‐CDDP‐dBET6@CM promotes immune recognition through multiple innate immune axes. Analysis of immune‐related genes (Figure 6d) reinforced this conclusion, showing robust upregulation of genes involved in interferon signaling, cytokine secretion, and lymphocyte activation. To dissect the contribution of BRD4 degradation, we compared Mn‐CDDP@CM and Mn‐CDDP‐dBET6@CM groups. The addition of dBET6 significantly increased the number of DEGs (Figure  S24, Supporting Information) and expanded the affected pathways to include protein processing, chromatin remodeling, cell cycle control, and DNA repair (Figure 6e). Notably, alterations in ribosome‐related pathways were also observed. Given the critical role of ribosome biogenesis in cancer cell proliferation, this suppression could synergize with the direct transcriptional inhibition of oncogenes to compromise tumor cell viability. GO analysis (Figure S25, Supporting Information) further highlighted gene clusters related to DNA damage, senescence, and proliferation arrest. A focused heatmap of DNA‐sensing‐related genes (Figure 6f) revealed elevated expression of key players in DNA damage and senescence signaling in the Mn‐CDDP‐dBET6@CM group, supporting dBET6's role in amplifying cytosolic DNA stress responses.^[^ ^57^ ^]^ Likewise, cell cycle gene analysis (Figure S26, Supporting Information) confirmed the transcriptomic signature of G0/G1 arrest, in agreement with previous flow cytometry data. Altogether, the RNA‐seq data provide strong molecular evidence that Mn‐CDDP‐dBET6@CM orchestrates a multilayered response encompassing genotoxic stress, cell cycle disruption, senescence induction, and innate immune activation. These findings not only consolidate the results of our cellular experiments but also support the mechanistic rationale for the nanoplatform's immunotherapeutic potential.

### 2.7 Mn‐CDDP‐dBET6@CM Possesses Excellent Tumor Targeting and Induces Systemic Anti‐Tumor Immune Response In Vivo

**Figure 7:**
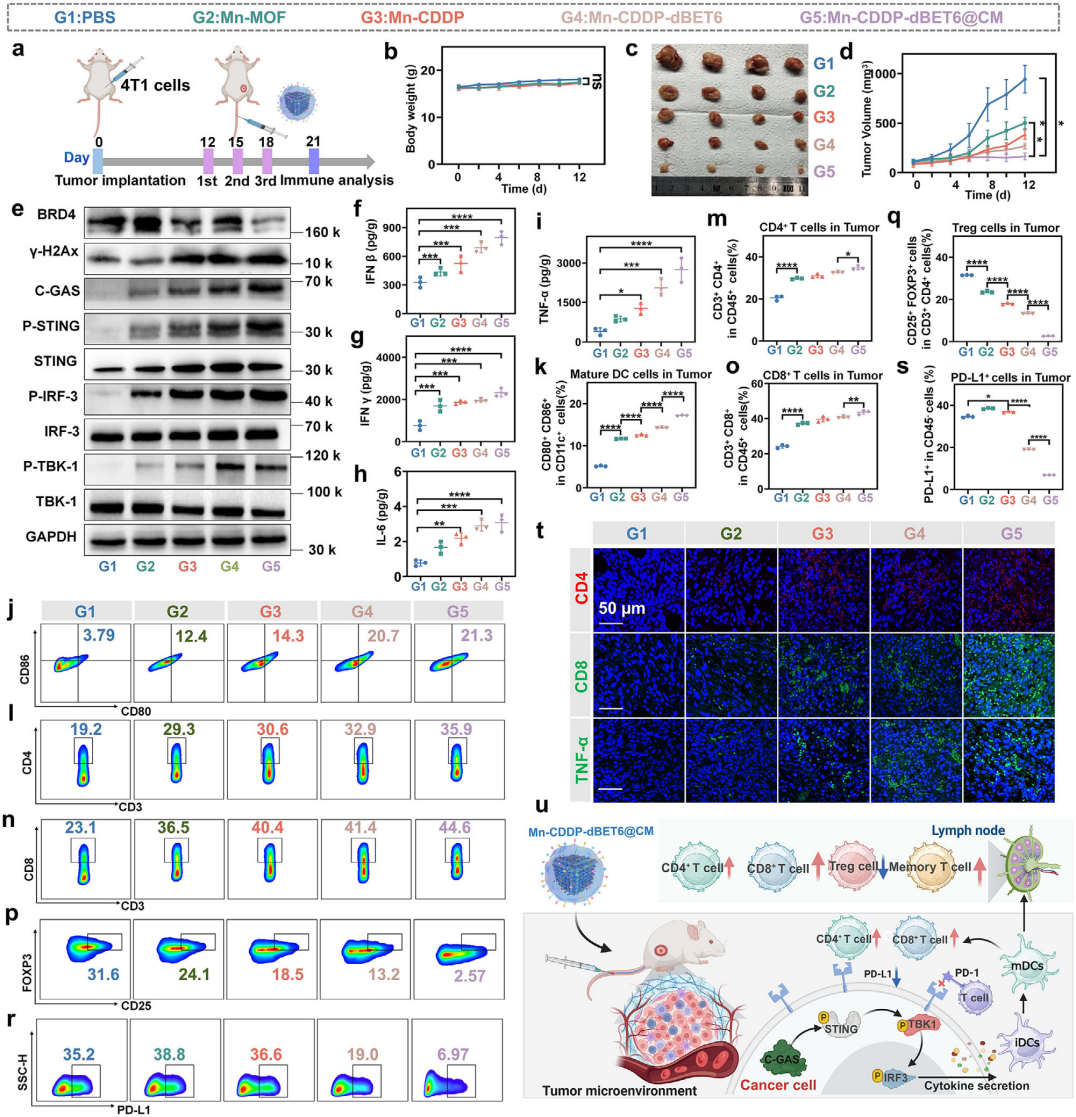
Therapeutic efficacy and immune activation induced by Mn‐CDDP‐dBET6@CM in vivo. a) Schematic diagram of the treatment schedule in the 4T1 tumor‐bearing mice model. b) Body weight changes of mice during treatment (n = 5). c) Representative photographs of excised tumor at the end of the study. d) Tumor growth curves following different treatments. e) Western blot analysis of cGAS‐STING pathway‐related protein expression in tumor tissues. f–i) ELISA‐based quantification of cytokines in tumor homogenates, including IFN‐β (f), IFN‐γ (g), IL‐6 (h), and TNF‐α (i). j) Representative flow cytometry plots showing the DCs in tumor tissues. k) Quantitative analysis of CD11c⁺ CD80⁺ CD86⁺ mature DCs populations. l, n, p) Representative flow cytometry plots of tumor‐infiltrating CD3⁺ CD4⁺ T cells, CD3⁺ CD8⁺ T cells, and CD25⁺ FOXP3⁺ Treg cells, respectively. m, o, q) Quantitative analysis of the corresponding immune cell populations. r) Flow cytometry analysis of PD‐L1 expression levels on tumor cells. s) Quantification of PD‐L1⁺ tumor cell percentages across treatment groups. t) Immunofluorescence section staining images of CD4, CD8, TNF‐α. u) Schematic representation of the proposed mechanism of immune activation mediated by Mn‐CDDP‐dBET6@CM in vivo. Data presented as mean ± S.D. (n = 3). *P < 0.05, **P < 0.01, ***P < 0.001, ****P < 0.0001.

After verifying the immunomodulatory potential of Mn‐CDDP‐dBET6@CM in vitro, the antineoplastic efficiency of Mn‐CDDP‐dBET6@CM was evaluated in 4T1 tumor‐bearing BALB/c mice. We first assessed the in vivo tumor‐targeting ability of the nanoplatform. Cy5‐labelled Mn‐CDDP‐dBET6@4T1 CM, Mn‐CDDP‐dBET6@B16F10 CM, and Mn‐CDDP‐dBET6 were administered to 4T1‐luc‐bearing mice, and real‐time fluorescence imaging revealed that the homologous membrane‐camouflaged formulation achieved significantly enhanced accumulation at the tumor site compared with heterologous cell membrane‐coated or uncoated controls. The signal persisted for more than 48 hours, and quantitative MFI analysis further confirmed the superior tumor‐targeting performance of the homologous strategy (Figure  S27a,b, Supporting Information). Ex vivo fluorescence imaging conducted 72 hours post‐injection demonstrated that fluorescence signals were predominantly retained in the tumor with minimal distribution in liver or lymph nodes (Figure S28a,b, Supporting Information), indicating effective tumor targeting and low off‐target accumulation. Following confirmation of tumor targeting, the anti‐tumor efficacy of Mn‐CDDP‐dBET6@CM was evaluated. The mice were randomly divided into 5 groups. 4T1 cells were subcutaneously inoculated into BALB/c mice. When tumor volumes reached approximately 80 mm^3^, the mice were intravenously injected with different nanoplatform. The treatment scheme is shown in **Figure** **7**a. As shown in Figure 7b, mice receiving the treatment showed no significant body weight fluctuations, indicating good biosafety and tolerance. Among all groups, Mn‐CDDP‐dBET6@CM exhibited the most potent tumor growth inhibition, as evidenced by tumor volume (Figure 7c,d), individual tumor growth curves (Figure S29, Supporting Information), and endpoint tumor weight (Figure  S30, Supporting Information), while TUNEL staining confirmed increased apoptotic cell populations (Figure S31, Supporting Information). These findings demonstrate that the combinatorial nanoplatform achieved efficient in vivo tumor suppression. Hematoxylin and eosin (H&E) staining of tumor tissues revealed disordered architecture and extensive necrosis in the Mn‐CDDP‐dBET6@CM group (Figure S32, Supporting Information), In contrast, no significant lesions were observed in major organs such as liver, heart, lung, spleen or kidney (Figure S33, Supporting Information), highlighting the biocompatibility of the formulation.

Given its strong tumor suppression capacity, we next explored whether Mn‐CDDP‐dBET6@CM could activate the innate immune pathway in vivo. Western blot analysis of tumor lysates showed significantly upregulated expression of cGAS and STING proteins (Figure 7e, uncropped blot image shown in Figure S34, Supporting Information), suggesting the activation of cytosolic DNA‐sensing signaling. In parallel, ELISA results revealed that cytokine levels of IFN‐β, IFN‐γ, IL‐6, and TNF‐α were elevated by 2.42∼6.87 fold following Mn‐CDDP‐dBET6@CM treatment relative to untreated cells (Figure 7f–i), indicating robust innate immune activation via the STING pathway. To further dissect immune remodeling within the TME, flow cytometric analysis was conducted. The proportion of mature dendritic cells (CD11c⁺ CD80⁺ CD86⁺) markedly increased following treatment (Figure 7j,k; gating strategy shown in Figure S17, Supporting Information), indicative of improved antigen presentation. Additionally, both CD4⁺ T cells (Figure 7l,m) and CD8⁺ T cells (Figure 7n,o, gating strategy shown in Figure S18, Supporting Information) were enriched in tumor tissues, increasing from from 24.16±0.71% in the control group to 43.67±0.91% in the Mn‐CDDP‐dBET6@CM‐treated group, corresponding to a 1.80‐fold increase, respectively, reflecting effective T cell recruitment and activation. Conversely, the immunosuppressive CD25⁺ FOXP3⁺ Treg cell population was reduced from 31.6±0.06% in the control group to 2.68±0.08% in the treated group (Figure 7p,q; gating strategy shown in Figure S35, Supporting Information), corresponding to a 11.7‐fold decrease, signifying relief from the immunosuppressive TME. Interestingly, PD‐L1 expression in tumor cells was significantly downregulated in the BRD4 degradation group (Figure 7r,s; gating strategy shown in Figure S36, Supporting Information), consistent with the previously described mechanism. In contrast, other groups showed upregulated PD‐L1 expression, indicating activation of the immune checkpoint axis, which provides a rationale for potential combination therapies with checkpoint blockade.^[^ ^58^ ^]^

The same conclusion was verified by evaluating immunofluorescence section staining images of tumors (Figure 7t). Additionally, upregulation of MHC‐I expression in tumor tissues (Figure S37, gating strategy shown in Figure S34, Supporting Information) supported enhanced antigen presentation and immune recognition.

In parallel, systemic immune responses were also evaluated. In the draining lymph nodes, Mn‐CDDP‐dBET6@CM promoted DCs maturation (Figure S38a,b, Supporting Information gating strategy shown in Figure S17, Supporting Information) and enhanced the proportions of CD4^+^ and CD8^+^ T cells (Figure S37c–f, Supporting Information gating strategy shown in Figure S18, Supporting Information), including a significant increase in IFN‐γ‐producing CD8^+^ effector T cells (Figure S38g,h, Supporting Information gating strategy shown in Figure S18, Supporting Information), reflecting systemic immune activation beyond the local tumor site.

To assess whether the therapeutic response could initiate long‐term immunological memory, we analyzed memory T cell subpopulations in lymph nodes after treatment. The Mn‐CDDP‐dBET6@CM group exhibited elevated levels of CD4⁺ effector memory T cells (T_EM_, CD44^+^ CD62L^−^, Figure S39a,b, Supporting Information, gating strategy shown in Figure S40, Supporting Information) and CD8^+^ central memory T cells (T_CM_, CD44^+^ CD62L^+^, Figure S39c,d, Supporting Information gating strategy shown in Figure S40, Supporting Information), suggesting that this strategy not only induces immediate tumor clearance but also primes the immune system for sustained surveillance. Figure 7u schematically illustrates the immune activation induced by Mn‐CDDP‐dBET6@CM, highlighting enhanced infiltration of effector T cells and the establishment of long‐lasting immune memory.

Taken together, Mn‐CDDP‐dBET6@CM demonstrates excellent tumor‐targeting ability and in vivo biosafety, effectively inducing nucleus DNA damage while robustly activating both innate and adaptive immune responses. By remodeling the immunosuppressive microenvironment, promoting T cell infiltration, and generating durable immune memory, this nanoplatform embodies a well‐designed, multifaceted approach that integrates metal ion‐mediated immune stimulation with PROTAC‐driven epigenetic modulation, offering a promising and precise strategy for cancer immunotherapy.

## 3 Conclusion

In conclusion, we have developed a rationally engineered nanoplatform, Mn‐CDDP‐dBET6@CM, that synergistically integrates metalloimmunotherapy, chemotherapy, senescence induction, and PROTAC‐mediated epigenetic modulation into a single system for enhanced cancer immunotherapy. By co‐delivering Mn^2+^, CDDP, and the BRD4‐targeting PROTAC dBET6 within a tumor cell membrane‐camouflaged manganese metal‐organic framework, this platform achieves triple activation of the cGAS‐STING pathway via Mn^2+^‐induced immune sensing, nucleus DNA damage, and BRD4 degradation‐mediated mitochondrial dysfunction. Simultaneously, BRD4 degradation suppresses PD‐L1 expression and promotes cellular senescence, collectively overcoming CDDP resistance and immune checkpoint‐mediated immunosuppression. The tumor cell membrane coating facilitates homologous targeting, immune evasion, and enhanced tumor accumulation. Compared with previous nanodelivery systems, which often suffer from insufficient tumor targeting, limited STING activation, and lack of synergy with epigenetic modulators or PROTACs, our platform overcomes these limitations by integrating precise tumor targeting, combinatorial DNA damage, immune activation, and PROTAC‐enhanced checkpoint modulation into a single system. Together, these features establish a closed‐loop, self‐amplifying immune activation circuit that drives robust innate and adaptive immune responses. This work demonstrates the potential of leveraging PROTAC‐enabled epigenetic remodeling to augment traditional chemo‐metalloimmunotherapy, offering a versatile and precise strategy for the development of next‐generation immunotherapeutic nanomedicines.

## Conflict of Interest

The authors declare no conflict of interest.

## Author Contributions

J.Z. and Z.C. conceived and designed this study. Z.C. performed the experiments, analyzed the results, drew the figures, and wrote the manuscript. Z.F. modeled part of the scheme. S.W. proofread the format of the manuscript. J.Z. provided funding and oversaw the project. All authors reviewed the figures and approved the final version of the manuscript.

## Supporting information



Supporting Information

## Data Availability

The data that support the findings of this study are available from the corresponding author upon reasonable request.
