# Supplementary material for: Engineering Metal‐Organic‐Framework‐Based STING Nanoagonists for PROTAC‐Enhanced Cancer Chemo‐Metalloimmunotherapy
Source: Adv Sci (Weinh). 2025 Oct 13;13(2):e15006. doi: 10.1002/advs.202515006 (PMC12786333; doi:10.1002/advs.202515006)
Supplement: Supplementary file 1 — Supporting Information [file ADVS-13-e15006-s001.docx]

Supplementary information

Engineering metal-organic-framework-based STING nanoagonists for PROTAC-enhanced cancer chemo-metalloimmunotherapy

*Zhenzhen Chen, Zhe Feng, Siyuan Wang, and* *Jingjing Zhang**

Table of Contents

**Experimental ProceduresS3**

Materials and reagentsS3

Synthesis of Mn-CDDP-dBET6@CM Nanoplatform S3

Coating with 4T1 Cell Membranes S4

Characterizations S4

Membrane Protein Analysis by SDS-PAGE S4

Nanoparticle Stability and Serum Protein Adsorption S4

Dynamic Light Scattering (DLS) and Zeta Potential S5

pH-Responsive Drug Release Assay S5

Cell Culture S5

Cellular Uptake and Targeting Specificity Analysis S5

Cell Viability Assay S5

BRD4 Degradation and DNA Damage Detection S6

Western Blot Analysis S6

Flow cytometry analysis of cell cycle S6

Cellular Senescence Assay S6

Mitochondrial Dysfunction Assessment S7

Flow Cytometry and Immunofluorescence for PD-L1 Expression S7

STING Pathway Activation and Immune Cell Co-culture S7

RNA Sequencing AnalysisS7

Animal model S8

Tumor Growth and Body Weight Monitoring S8

H&E and immunohistochemical staining S8

Flow Cytometry Analysis of Tumor and Lymph Node Immune Cells S8

Memory T Cell Analysis (TEM/TCM) S9

Cytokine Quantification by ELISA S9

TUNEL Assay for Apoptosis S9

Immunofluorescence Staining of Tumor Sections S9

Statistical Analysis S9

**Supporting FiguresS11-S50**

Experimental Procedures

***Materials and reagents***

Manganese acetate (Mn(CH_3_COO)_2_∙4H_2_O, 99.99%, Cat# 229776), polyvinylpyrrolidone (PVP, K-30, Cat#81420), and potassium hexacyanocobaltate (K_3_[Co(CN)_6_], Cat#218642) were purchased from Sigma-Aldrich, Cisplatin (CDDP, 99.63%, Cat#T1564) were purchased from TargetMol. dBET6 (99.90%, Cat#HY-112588) was obtained from MedChemExpress. Cyanine5- CDDP (≥95%) obtained was obtained from QIYUE BIOLOGY. Ethylenediaminetetraacetic acid disodium salt dihydrate (EDTA) was obtained from Sigma-Aldrich. All solutions were prepared with Millipore water (18.25 MΩ·cm^-1^).

Cell culture reagents including RPMI-1640 medium, phosphate buffered saline (1×PBS) (10 mM, pH 7.4), fetal bovine serum (FBS), and cell cycle analysis kit with propidium iodide (PI) staining were bought from Keygen Biotech. Annexin V-FITC/PI Apoptosis Detection Kit was purchased from Vazyme Biotech Co., Ltd. Live & Dead TM Viability/ Cytotoxicity Assay Kit for Animal Cells (Calcein AM, PI), Cell Counting Kit-8, Mito-Tracker Deep Red FM, Mitochondrial Membrane Potential Assay Kit, and β-galactosidase staining kit were purchased from Beyotime. LysoTracker Green DND-26 was obteined from Yeasen Biotechnology. ELISA kits for cytokine detection, including IFN-β, IFN-γ, IL-6, and TNF-α were obtained from Thermo Fisher.

Western blots were performed with antibodies against of BRD4 (28486-1-AP, Proteintech, 1:2000), γH2AX (9718, Cell Signaling Technology, 1:2000), RAD51 (8875, Cell Signaling Technology, 1:2000), p21 (2947, Cell Signaling Technology, 1:2000), p16 (23200, Cell Signaling Technology, 1:2000), Clipped H3 (68345-1-Ig, Proteintech, 1:1000), cGAS (31659, Cell Signaling Technology, 1:1000), STING (13647, Cell Signaling Technology, 1:1000), Phospho-STING (72971, Cell Signaling Technology, 1:1000), TBK1/NAK (3504, Cell Signaling Technology, 1:1000), Phospho-TBK1/NAK (5483, Cell Signaling Technology, 1:1000), IRF-3 (4302, Cell Signaling Technology, 1:1000), Phospho-IRF-3 (29047, Cell Signaling Technology, 1:1000), PD-L1 (ab213480, abcam,1:1000), β-Actin (4967, Cell Signaling Technology, 1:1000), GAPDH (2118, Cell Signaling Technology, 1:1000), and anti-rabbit IgG, HRP-conjugated secondary antibody (#7074, Cell Signaling Technology, 1:5000).

Immunofluorescence were performed with antibodies against of BRD4 (28486-1-AP, Proteintech, 1:200), γH2AX (9718, Cell Signaling Technology, 1:200), CD4 (ab183685, abcam, 1:200), CD8 (ab217344, abcam, 1:200), TNF-α (ab2151188, abcam, 1:200), cy5-conjugated secondary antibody (ab6564, abcam, 1:1000).

The antibodies used in Immunoassays include PerCP/Cyanine5.5-labeled anti-mouse CD11c (117327), PE-labeled anti-mouse CD80 (104707), PE/Cyanine7-labeled anti-mouse CD86 (105013), PE-labeled anti-mouse CD45 (103105), Pacific Blue-labeled anti-mouse CD3 (100214), PE-labeled anti-mouse CD8 (100708), PE/Cyanine7-labeled anti-mouse CD4 (100422), APC-labeled anti-mouse PD-L1 (124311), FITC-labeled anti-mouse MHC I (116605), FITC-labeled anti-mouse/human CD44 (103005), PerCP/Cyanine5.5-labeled anti-mouse CD62L (104431), APC-labeled anti-mouse CD25 (101909), FITC-labeled Anti-Mouse Granzyme B (515403), and APC anti-mouse CD95 (Fas) (152603) were purchased from Biolegend. FITC-labeled Anti-Mouse FOXP3 (ab210230) was purchased from Abcam. FITC-labeled Anti-Mouse IFN-γ (12-7311-82) and FITC anti-mouse CD178 (FasL) (11-5911-82) were purchased from Thermo Fisher.

All animal experiments were approved by the Institutional Animal Care and Use Committee (IACUC) of Nanjing University (Approval No: IACUC-2401003). 6-8 week-old BALB/c mice (female) were provided by Gempharmatech.

***Synthesis of Mn-CDDP-dBET6@CM Nanoplatform***

For the synthesis of Mn-CDDP-dBET6 nanoparticles, solution A: K_3_[CoIII(CN)_6_] (0.15 mmol) and PVP K-30 (0.3 g) were dispersed in mixed water/ethanol solution (5/15 mL). Subsequently, solution B: Mn(CH_3_COO)_2_∙4H_2_O (0.1 mmol), CDDP (10 mg, dissolved in DMSO), and dBET6 (200 mM in DMSO) in water (10 mL) was added into solution A with stirring. Milk-white turbidity was generated immediately upon the addition. All samples were collected after centrifugation with washing using deionized H_2_O (5 mL) twice (9000 rpm, 15 minutes). The products were dried under vacuum and collected after crushing into fine powder. For the synthesis of (cy5)-labeled Mn-CDDP-dBET6 nanoparticles, CDDP was replaced by cy5-labeled CDDP.

***Coating with 4T1 Cell Membranes***

Cell membranes were extracted from 4T1 cells via repeated freeze-thaw cycles and differential centrifugation, followed by ultracentrifugation (100,000 × g, 1 h, 4 °C). Membrane vesicles were mixed with Mn-CDDP-dBET6 at a mass ratio of 1:1 and subjected to extrusion through a 200 nm polycarbonate membrane using a mini-extruder (Avanti) to yield Mn-CDDP-dBET6@CM. The resulting nanoparticles were collected by centrifugation and washed.

***Characterizations***

The transmission electron microscopy (TEM) images were collected by a JEOL JEM 2100plus microscopy at an accelerating voltage of 200 kV. Powder X-ray diffraction (XRD) patterns were acquired on a MiniFlex300/600 (Rigaku) equipped with Cu/Kα radiation (λ = 1.5418 Å). For measurements of Zeta-potential and size distribution, a DLS instrument (Nano ZS ZEN3600, Malvern) was used.

***Membrane Protein Analysis by SDS-PAGE***

Protein extraction from native 4T1 membranes and Mn-CDDP-dBET6@CM was performed using RIPA buffer. Equal amounts of protein (10 μg) were loaded onto a 10% SDS-PAGE gel and visualized by Coomassie Brilliant Blue staining. Band profiles were compared to assess membrane protein retention.

***Nanoparticle Stability and Serum Protein Adsorption***

Mn-CDDP-dBET6@CM nanoparticles were suspended in PBS or RPMI-1640 medium containing 10% fetal bovine serum (FBS) at a concentration of 50 μg /mL and incubated at 37 °C with gentle shaking. Particle sizes were measured by dynamic light scattering at 2, 4, 6, 12, and 24 h to assess colloidal stability.

***Dynamic Light Scattering (DLS) and Zeta Potential***

Hydrodynamic size and surface zeta potential were measured using a Malvern Zetasizer Nano ZS at 25 °C. Samples were dispersed in PBS at a concentration of 0.1 mg/mL and analyzed in triplicate.

***Hemolysis Assay***

Whole blood was collected from healthy Balb/c mice. Red blood cells (RBC) were obtained through centrifugation and suspended into PBS solution. Next, RBC solutions (0.5 mL) were mixed with PBS solution (0.5 mL) containing 5-50 μg/mL of Mn-CDDP-dBET6@CM. PBS solution and distilled water were used as negative control (0% hemolysis) and positive control (100% hemolysis), respectively. After incubation at room temperature, the samples were centrifuged. The absorbance of supernatants at 541 nm were measured with a Shimadzu UV-3600 spectrometer. The hemolysis rate (HR%) was calculated with equation: HR% = [(A_testing_ - A_negative_ _control_) / ( A_positive control_ - A_negative control_)] × 100%.

***pH-Responsive Drug Release Assay***

Mn-CDDP-dBET6@CM nanoparticles were dispersed in PBS at pH 7.4 or 5.6 (50 μg/mL) and incubated at 37 °C under gentle shaking. At designated time points, samples were centrifuged at 12,000 rpm for 10 min, and supernatants were collected. The morphological changes of Mn-CDDP-dBET6 were monitored by transmission electron microscope (TEM, JEOL JEM 2100plus). Aliquots were filtered through 0.22 μm membranes for dBET6 quantification by high-performance liquid chromatography (HPLC). The concentrations of Mn and Pt in the collected solutions were measured by ICP-MS (EXPEC 7200, EXPEC Technology, China).

***Cell Culture***

HEK-293T, 4T1, and B16F10 cells purchased from ATCC were used as cell models for this study. They were cultured in a cell culture incubator (37°C, 5% CO2) using RPMI 1640 medium containing 10% FBS, 100 U/mL penicillin and 100 μg/mL streptomycin. Dendritic cells (DCs), and T cells were cultured in RPMI 1640 medium supplemented with 10% fetal bovine serum, 1% penicillin-streptomycin, and 2 mM L-glutamine. Cells were maintained at 37 °C in a humidified atmosphere with 5% CO_2_. For immune cell co-culture assays, DCs and T cells were isolated from spleens of BALB/c mice using magnetic bead separation and co-cultured with 4T1 cells.

***Cellular Uptake and Targeting Specificity Analysis***

Nanoparticles for synthesizing used Cyanine5- CDDP were incubated with 4T1 breast cancer cells, B16F10 melanoma cells, and HEK-293T cells. Cells were exposed to nanoparticles at predetermined concentrations (50 μg/mL) and time points. Flow cytometry quantified intracellular nanoparticle fluorescence intensity across different cell types. Images were acquired using a Leica TCS SP8 confocal laser scanning microscope equipped with a 63× oil-immersion visualized intracellular localization and lysosomal escape using LysoTracker staining (Beyotime). Cytotoxicity of nanoparticles was evaluated via the CCK-8 assay after 24 h treatment at varying doses to assess selective cytotoxic effects.

***Cell Viability Assay***

To evaluate tumor selectivity and biocompatibility, cell viability was assessed in homologous (4T1), heterologous (B16F10), and non-malignant (HEK-293T) cell lines. Cells were seeded into 96-well plates at a density of 5 × 10^3^ cells/well and allowed to adhere overnight. The following day, cells were treated with Mn-CDDP-dBET6@CM at various concentrations for 24 h. Cell viability was measured using a Cell Counting Kit-8 following the manufacturer's instructions. After adding 10 μL CCK-8 solution per well, plates were incubated for 2 h at 37 °C, and absorbance at 450 nm was recorded using a microplate reader. Cell viability was normalized to untreated controls. All conditions were tested in triplicate. To determine the different groups obtained cytotoxicity 4T1 cells were seeded into 96-well plates at 5 × 10^3^ cells/well and allowed to adhere overnight. Cells were treated with various formulations (Mn-MOF, Mn-CDDP, Mn-CDDP-dBET6, and Mn-CDDP-dBET6@CM, 50 μg/mL) at equivalent drug concentrations for 24 h. Same as above for other methods.

***BRD4 Degradation and DNA Damage Detection***

4T1 cells were seeded on glass coverslips and treated with the indicated formulations (Mn-MOF, Mn-CDDP, Mn-CDDP-dBET6, and Mn-CDDP-dBET6@CM, 50 μg/mL) for 24 h. Cells were fixed with 4% paraformaldehyde, permeabilized with 0.2% Triton X-100, and blocked with 5% BSA. Cells were incubated overnight at 4 °C with primary antibodies against BRD4 (Abcam, ab128874, 1:200) and γ-H2AX (Cell Signaling Technology, #9718, 1:200), followed by cy5-conjugated secondary antibody (ab6564, abcam, 1:5000). Nuclear were labelled with hoechst 33342 (1 μg/mL) for 5 min. Images were acquired using a Leica TCS SP8 confocal laser scanning microscope equipped with a 63× oil-immersion objective.

***Western Blot Analysis***

Cells and tumor tissues were lysed in RIPA buffer containing protease and phosphatase inhibitors. Equal amounts of protein were resolved by SDS-PAGE and transferred to PVDF membranes. After blocking with 5% BSA, membranes were incubated with appropriate primary antibodies (1:1,000-1:2000) overnight at 4 °C, followed by HRP-conjugated secondary antibodies (1:5,000) for 1 h at room temperature. Protein bands were visualized using enhanced chemiluminescence (ECL) and imaged using a chemiluminescence imaging system.

***Flow cytometry analysis of cell cycle***

4T1 cells were treated for 24 h, then fixed in 70% ethanol at -20 °C overnight. After washing with PBS, cells were stained with PI/RNase A solution for 30 min at room temperature in the dark. DNA content was analyzed by flow cytometry (BD FACSCanto II), and cell populations in G0/G1, S, and G2/M phases were quantified using Flowjo software.

***Cellular Senescence Assay***

Cellular senescence was evaluated using a Senescence β-Galactosidase Staining Kit (Beyotime, C0602). 4T1 cells were seeded in 12-well plates and treated with Mn-CDDP-dBET6@CM for 48 h. Cells were washed, fixed, and incubated with staining solution at 37 °C (no CO₂) overnight. Blue-stained senescent cells were observed under a bright-field microscope (Olympus IX71), and positive cells were quantified from random fields.

***Mitochondrial Dysfunction Assessment***

Mitochondrial membrane potential was evaluated using a JC-1 staining kit. Briefly, 4T1 cells were seeded in confocal dishes and treated with different groups for 24 h. Cells were washed with PBS and incubated with JC-1 working solution at 37 °C for 20 min. After rinsing, red (J-aggregates) and green (monomers) fluorescence signals were recorded using a Leica TCS SP8 confocal microscope.

***Flow Cytometry and Immunofluorescence for PD-L1 Expression***

To quantify surface PD-L1 levels, cells were treated with indicated formulations for 24 h, then harvested and stained with FITC-conjugated anti-PD-L1 antibody in the dark for 30 min at 4 °C. Flow cytometric analysis was performed on a BD FACSCanto II system, and data were analyzed with FlowJo software.

For immunofluorescence detection, cells were fixed, blocked, and incubated with primary antibody against PD-L1, followed by fluorescent secondary antibody. Nuclear were labelled with Hoechst 33342 (1 μg/mL) for 5 min, then fluorescence signals were recorded using a Leica TCS SP8 confocal microscope. All groups were processed under identical acquisition parameters.

***STING Pathway Activation and Immune Cell Co-culture***

Bone marrow-derived dendritic cells (BMDCs) were isolated from femurs of BALB/c mice and cultured in RPMI-1640 supplemented with 10% FBS, 20 ng/mL GM-CSF, and 10 ng/mL IL-4 (PeproTech). 4T1 cells pretreated with Mn-CDDP-dBET6@CM for 24 h were seeded in the lower chamber of Transwell inserts (0.4 μm pore size) at a density of 5 × 10^4^ cells/well. BMDCs and splenic T cells were co-seeded in the upper chamber at a 1:1 ratio to the tumor cells. After 48 h of co-culture, cells from both chambers were harvested for flow cytometric analysis. BMDC maturation was assessed by staining with anti-CD80 and anti-CD86 antibodies. T cell activation was evaluated by staining with anti-CD4 and anti-CD8 antibodies. For cytotoxic function analysis, CD8⁺ T cells were intracellularly stained with anti-Granzyme B antibody following fixation and permeabilization, and surface-stained with anti-Fas L antibody. Tumor cells from the lower chamber were collected and stained with anti-Fas antibody for analysis.

***RNA Sequencing Analysis***

4T1 cells were treated with PBS, Mn-MOF@CM, Mn-CDDP@CM, or Mn-CDDP-dBET6@CM for 24 h. Total RNA was extracted using a standard RNA isolation kit, and RNA integrity was confirmed with a Bioanalyzer. Sequencing libraries were prepared using poly(A) selection and sequenced on an Illumina platform to generate paired-end reads. Raw data were subjected to quality control using FastQC, followed by alignment to the mouse reference genome (GRCm38) using STAR. Transcript abundance was quantified using HTSeq, and differential expression analysis was performed with DESeq2. Genes with adjusted *p*-value < 0.05 and | log_2_ (fold change) | > 1 were considered differentially expressed. Gene Ontology (GO) and Kyoto Encyclopedia of Genes and Genomes (KEGG) pathway enrichment analyses were conducted using cluster Profiler. Immune-related gene sets were annotated based on published databases, and heatmaps were generated using pheatmap in R.

***Animal model***

The female BALB/c mice were inoculated subcutaneously with 5 × 10^5^ 4T1-luc cells in the right flank. When tumors reached ~100 mm³, mice were intravenously injected with Cy5-labeled Mn-CDDP-dBET6@CM, Mn-CDDP-dBET6@heterologous CM, or uncoated Mn-CDDP-dBET6 at a dose of 1 mg/kg. Each group received a single injection, and real-time whole-body fluorescence imaging was performed at 0, 6, 12, 24, 48, and 72 h post-injection using an IVIS Spectrum imaging system. To minimize pain and distress, mice were anesthetized with isoflurane inhalation prior to invasive procedures. At the designated experimental endpoints, mice were euthanized by CO₂ inhalation followed by cervical dislocation to ensure death, in accordance with the approved protocol. Blood and tissue samples were collected immediately after euthanasia for further analysis. Tumors and major organs were collected at 72 h post-injection for *ex vivo* fluorescence quantification.

***Tumor Growth and Body Weight Monitoring***

Tumor size was measured with a digital caliper every 2 days and calculated using the formula: volume = (length × width^2^) / 2. Body weights were recorded concurrently to monitor systemic toxicity. At the endpoint, mice were sacrificed, and tumors and major organs were excised for further analysis.

***H&E and immunohistochemical staining***

The mice were killed after treatment, and representative heart, liver, spleen, lung, kidney, and tumor tissues were collected for histology analysis. The tissues were sliced and dehydrated, dehydration and stained with hematoxylin and eosin (H&E). The H&E staining images were observed under the brightfield ***microscopy*** (Olympus, Japan).

***Flow Cytometry Analysis of Tumor and Lymph Node Immune Cells***

Tumor tissues and draining lymph nodes were harvested, minced, and digested in RPMI-1640 containing collagenase IV (1 mg/mL) and DNase I (50 U/mL) at 37 °C for 30  minutes. Resulting cell suspensions were filtered through 70 μm cell strainers and washed with PBS. Cells were stained with fluorescence-labeled antibodies targeting CD11c, CD80, CD86 (DCs), CD3, CD4, CD8 (Helper and cytotoxic T cells), CD25, FOXP3 (regulatory T cells), PD-L1, CD44, and CD62L (memory T cells), along with viability dyes to exclude dead cells. Samples were acquired on a BD FACSCanto II flow cytometer and analyzed using FlowJo software. All flow cytometry data were acquired with at least 100,000 events per sample, and dead cells/debris were excluded using FSC/SSC gating.

***Memory T Cell Analysis (T_EM_/T_CM_):***

Draining inguinal and axillary lymph nodes were harvested from tumor-bearing mice at the study endpoint. Lymph nodes were mechanically dissociated into single-cell suspensions by gentle grinding through a 70 μm nylon cell strainer, followed by red blood cell lysis. After washing, cells were resuspended in staining buffer and incubated with fluorophore-conjugated antibodies against CD3, CD4, CD8, CD44, and CD62L (BioLegend) for 30 min at 4 °C. Flow cytometry was performed using a BD LSRFortessa, and data were analyzed with FlowJo software. Effector memory T cells (T_EM_) were defined as CD3⁺CD4⁺CD44⁺CD62L⁻ while central memory T cells (T_CM_) were defined as CD3⁺CD8⁺CD44⁺CD62L⁺.

**Cytokine Quantification by ELISA**

Tumor tissues were homogenized in cold PBS containing protease inhibitors and centrifuged at 12,000 × g for 15 minutes at 4 °C. The supernatants were collected and analyzed for cytokine levels. Quantification of IFN-β, IFN-γ, IL-6, and TNF-α was performed using commercial ELISA kits following the manufacturer’s instructions. Briefly, samples and standards were added in duplicates to antibody-coated 96-well plates, incubated with detection antibodies, followed by streptavidin-HRP and TMB substrate. After stopping the reaction, absorbance was measured at 450 nm, and cytokine concentrations were calculated using standard curves. All procedures were performed at room temperature unless otherwise stated.

**TUNEL Assay for Apoptosis**

Terminal deoxynucleotidyl transferase dUTP nick end labeling (TUNEL) staining was performed on paraffin-embedded tumor sections using an In Situ Cell Death Detection Kit. After deparaffinization and proteinase K treatment, sections were incubated with TUNEL reaction mixture at 37 °C for 1 h in the dark. The tissue sections were prepared by the Servicebio Biotech Company (Wuhan, China) for histopathology evaluation. Fluorescence signals indicating apoptotic nuclear were visualized by confocal microscopy (Leica TCS SP8).

**Immunofluorescence Staining of Tumor Sections**

Tumor tissues were harvested and immediately embedded in OCT compound, then snap-frozen in liquid nitrogen. Frozen sections (8 μm) were prepared using a cryostat (Leica CM1950) and fixed with cold acetone for 10 min at -20 °C. After air drying, sections were rehydrated in PBS and blocked with 5% normal goat serum for 1 h at room temperature. Primary antibodies against target proteins were diluted in blocking buffer and incubated overnight at 4 °C. After washing with PBS, sections were incubated with species-appropriate Alexa Fluor-conjugated secondary antibodies for 1 h at room temperature in the dark. Nuclear were labelled with Hoechst 33342 (1 μg/mL) for 5 min. Slides were mounted with antifade reagent and imaged using a confocal laser scanning microscope (Leica TCS SP8).

**Statistical Analysis**

All data from at least three independent experiments were expressed as mean ± standard deviation (n = 3). The statistical difference between different groups of data was evaluated by one-way ANOVA, and *P* < 0.05 was considered to be statistically significant. Asterisk (*) *denotes statistical significance between bars (***P* < 0.05, ***P* < 0.01, ****P* < 0.001, *****P* < 0.0001). All statistical analyses were performed using GraphPad Prism software (version 9.4.1).

Supporting Figures

**
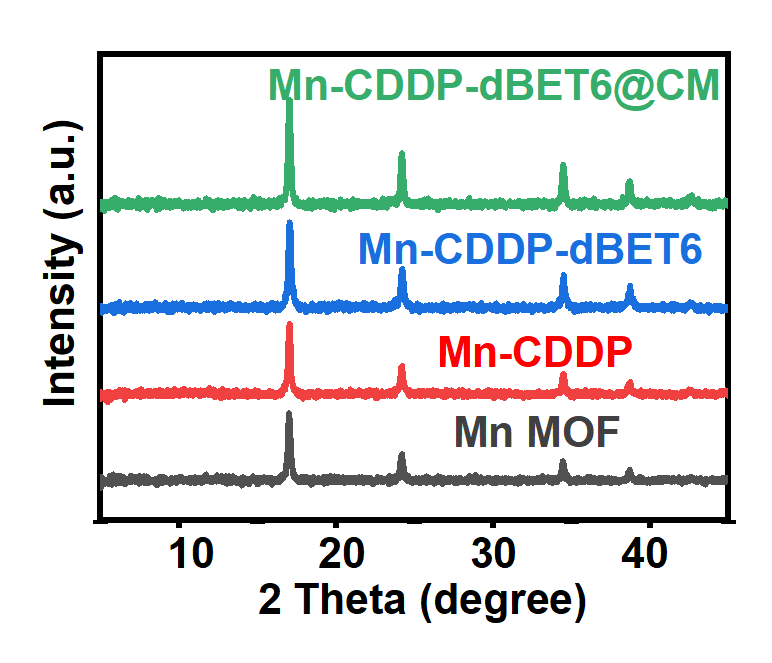
**

**Figure S1.** Powder XRD patterns of different formulations.

**
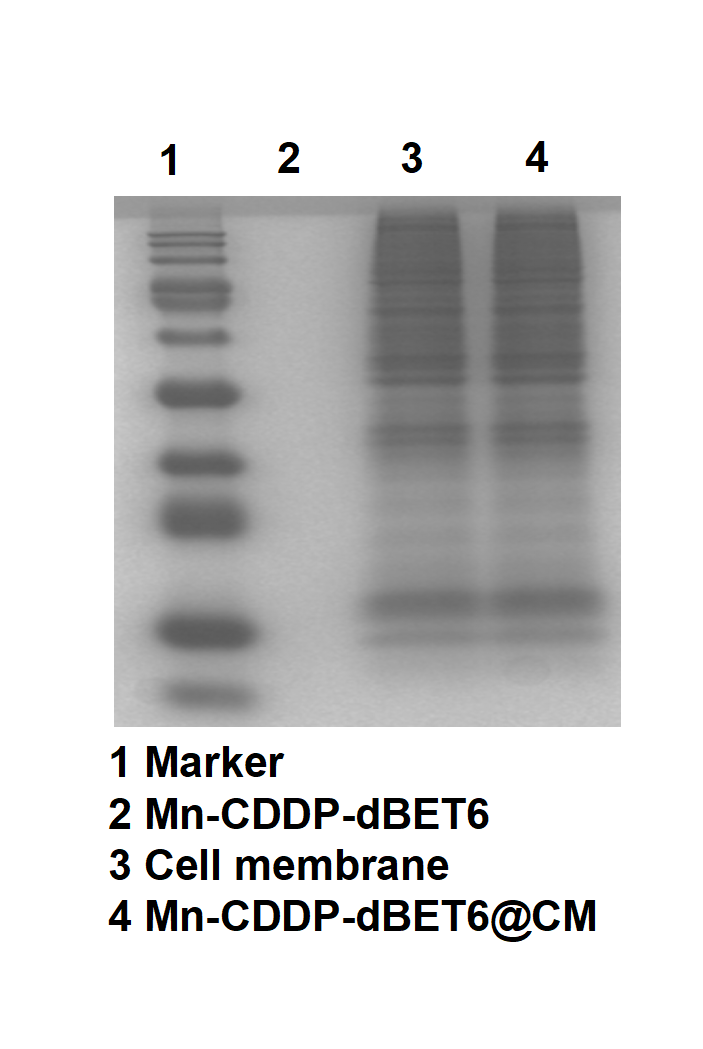
**

**Figure S2.** SDS-PAGE protein analysis of Mn-CDDP-dBET6, Cell membrane (CM), Mn-CDDP-dBET6@CM.

**
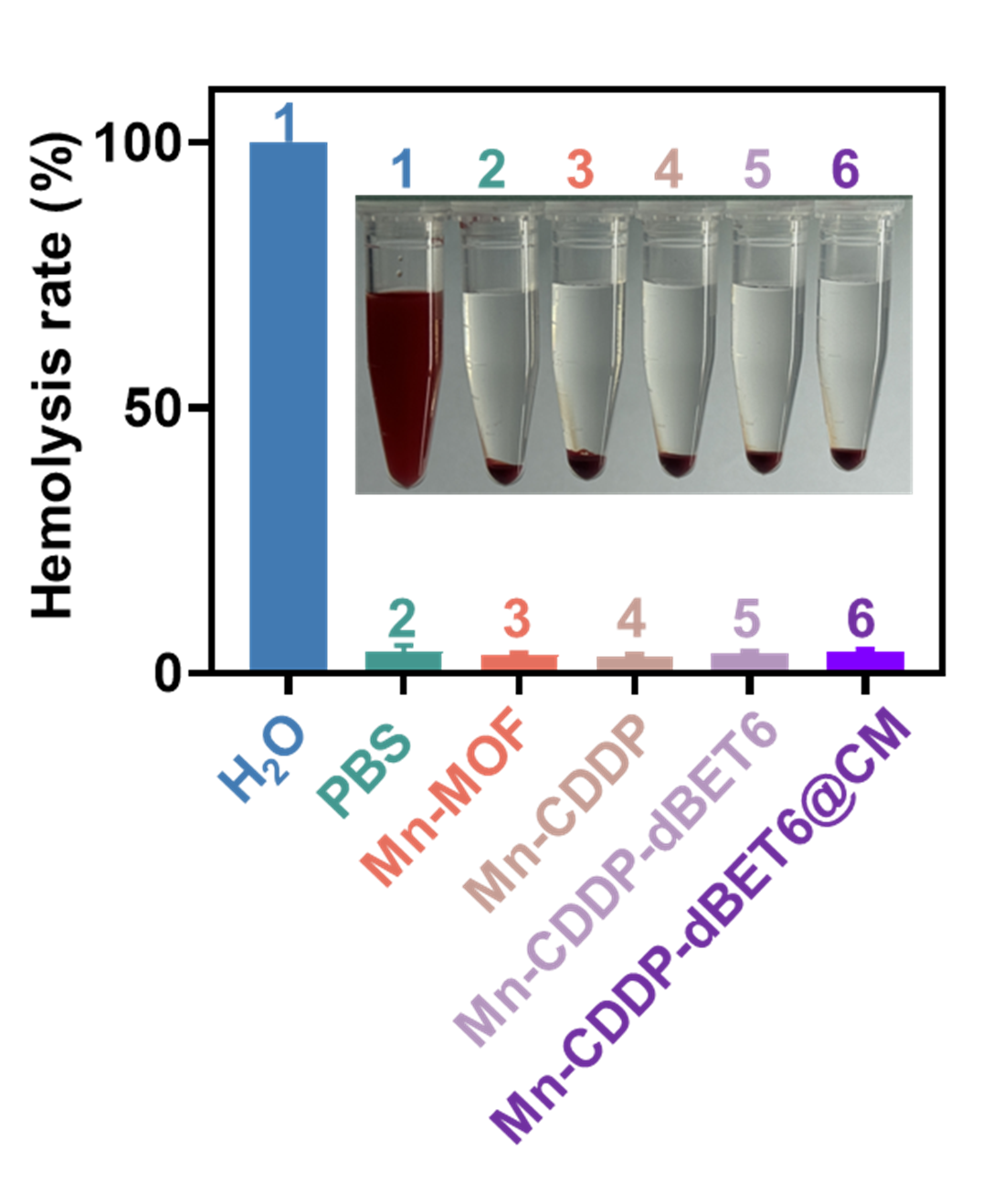
**

**Figure S3.** Representative photographs and relative hemolysis rates of RBCs treated with different formulations, using H_2_O as a positive control and PBS as a negative control.

**
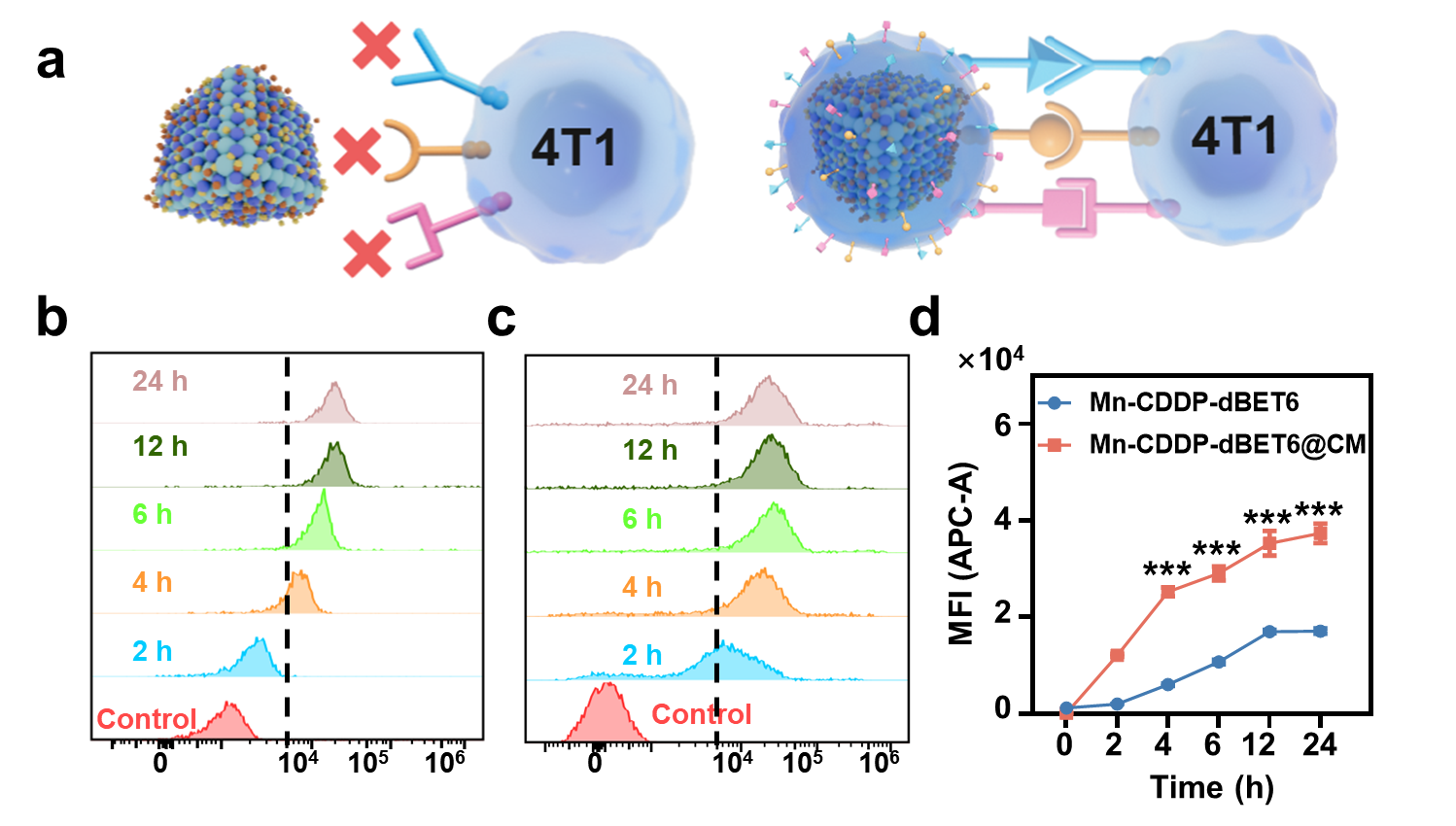
**

**Figure S4.** (a) Schematic representation of the experimental setup: 4T1 breast cancer cells are co-incubated with either Mn-CDDP-dBET6 or Mn-CDDP-dBET6@CM nanoparticles, followed by interaction with 4T1 to assess particle uptake. Flow cytometry histograms showing uptake of Mn-CDDP-dBET6 (b) and Mn-CDDP-dBET6@CM (c) by 4T1 after various times of coincubation and quantitative analysis (d). Data presented as mean ± S.D. (n=3). **P* < 0.05, ***P* < 0.01, ****P* < 0.001, *****P* < 0.0001.

**
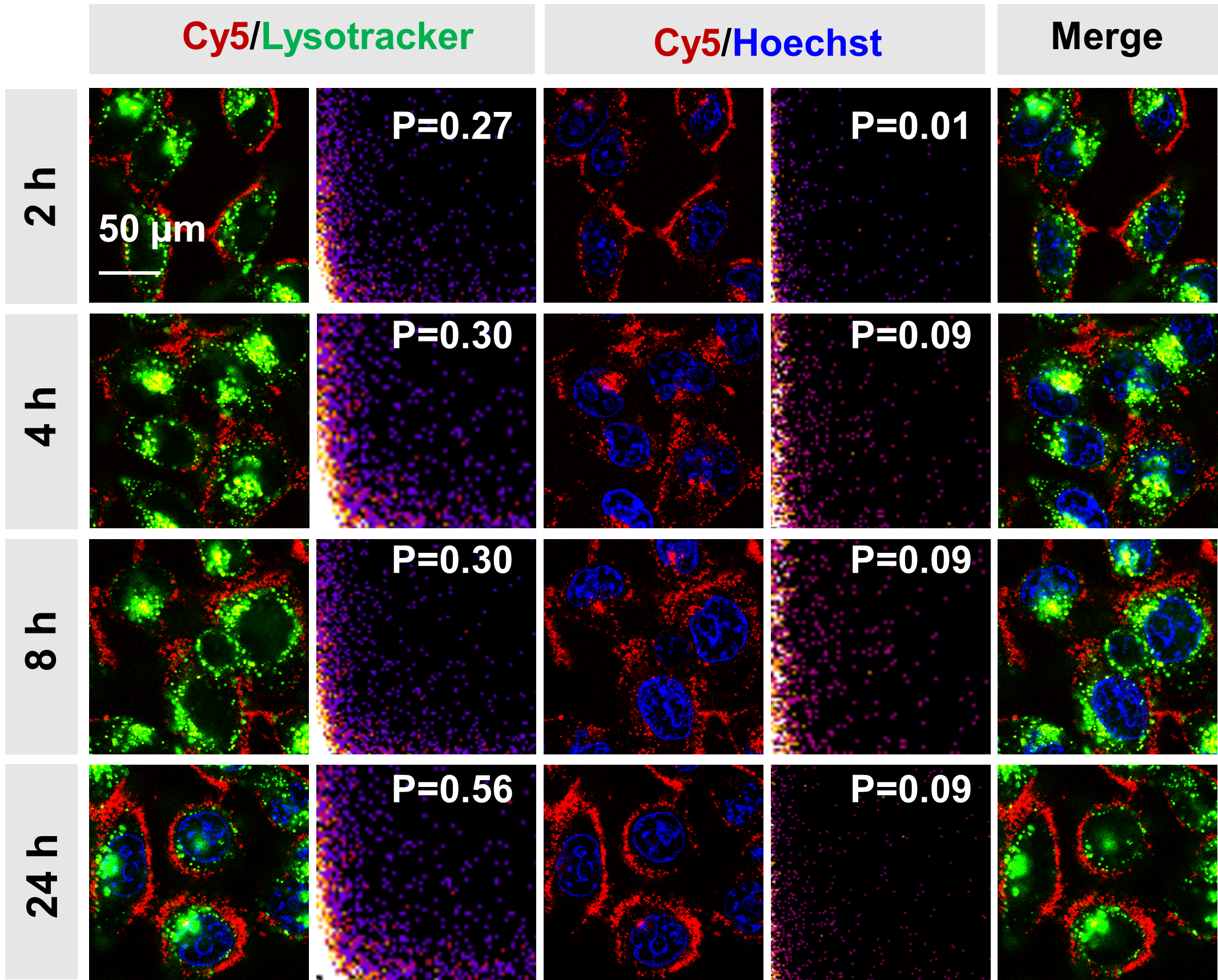
**

**Figure S5.** Time-dependent cellular uptake of Mn-CDDP-dBET6@4T1 CM in B16F10 cells observed by confocal laser scanning microscopy (CLSM).

**
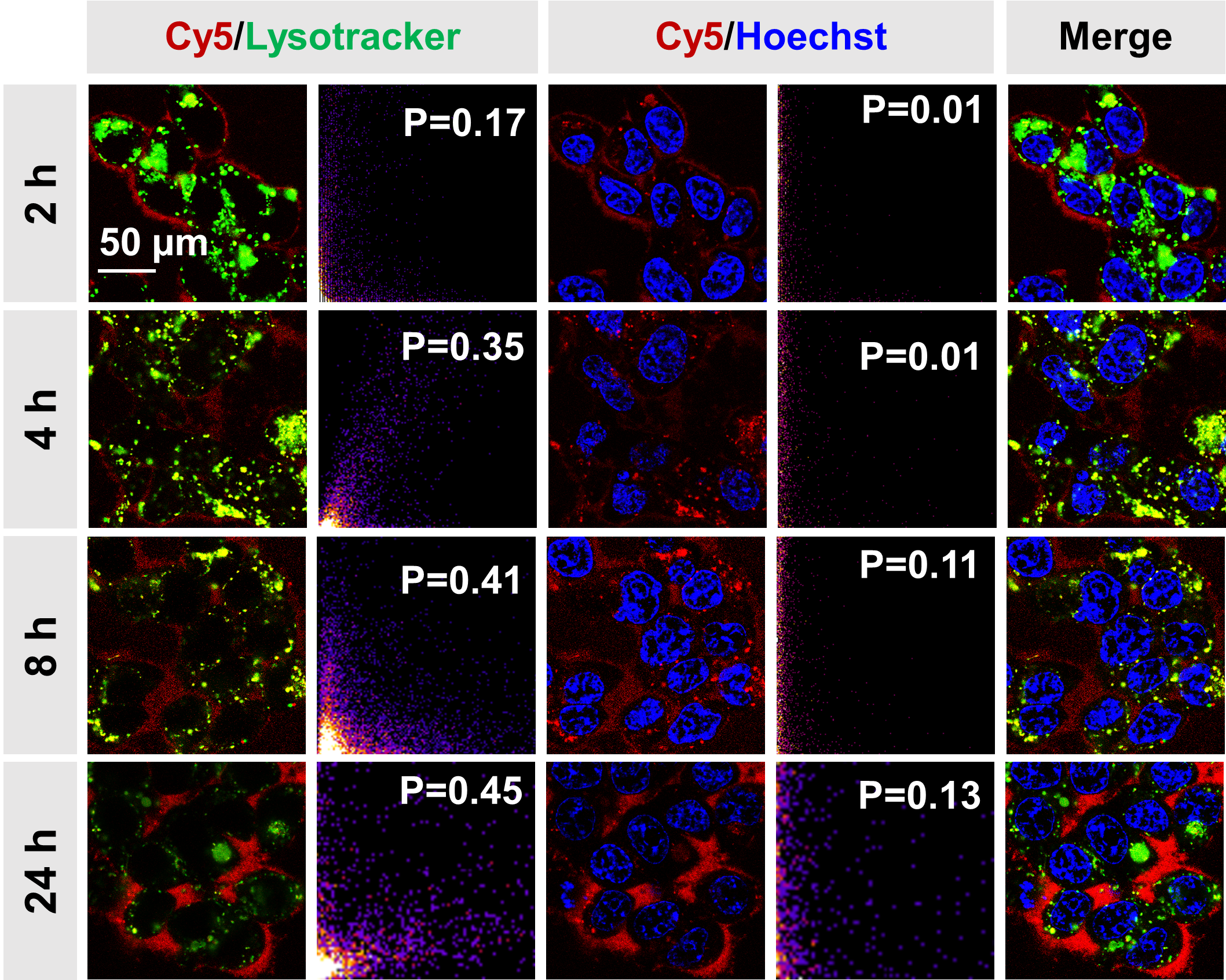
**

**Figure S6.** Time-dependent cellular uptake of Mn-CDDP-dBET6@4T1 CM in HEK-293T cells observed by confocal laser scanning microscopy (CLSM).

**
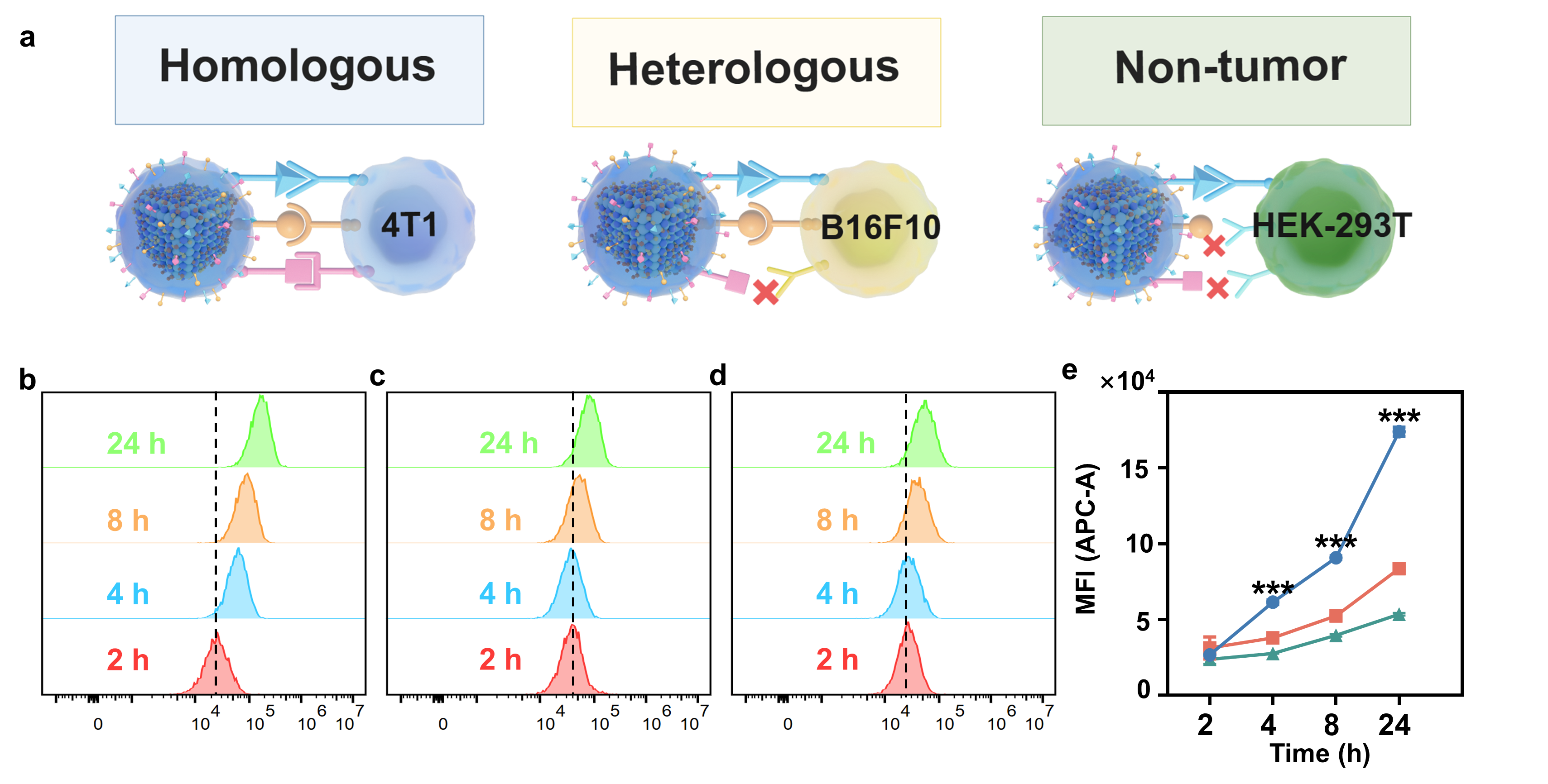
**

**Figure S7.** (a) Schematic description of HEK-293T, B16F10 and 4T1 cells with Mn-CDDP-dBET6@4T1 CM coincubated. Flow cytometry of cellular uptake of cy5-labeled Mn-CDDP-dBET6@4T1 CM with 4T1 cells (b), B16F10 cells (c) and HEK-293T (d) after various times of coincubation and quantitative analysis (e). Data presented as mean ± S.D. (n=3). **P* < 0.05, ***P* < 0.01, ****P* < 0.001, *****P* < 0.0001.

**
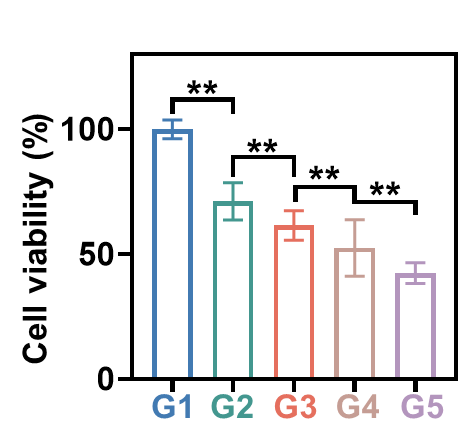
**

**Figure S8.** Cell viabilities of 4T1 cells after 24 h of incubation with varied nanoplatforms. G1: PBS, G2: Mn-MOF, G3: Mn-CDDP, G4: Mn-CDDP-dBET6, G5: Mn-CDDP-dBET6@CM. Data presented as mean ± S.D. (n=3). **P* < 0.05, ***P* < 0.01, ****P* < 0.001, *****P* < 0.0001.

**
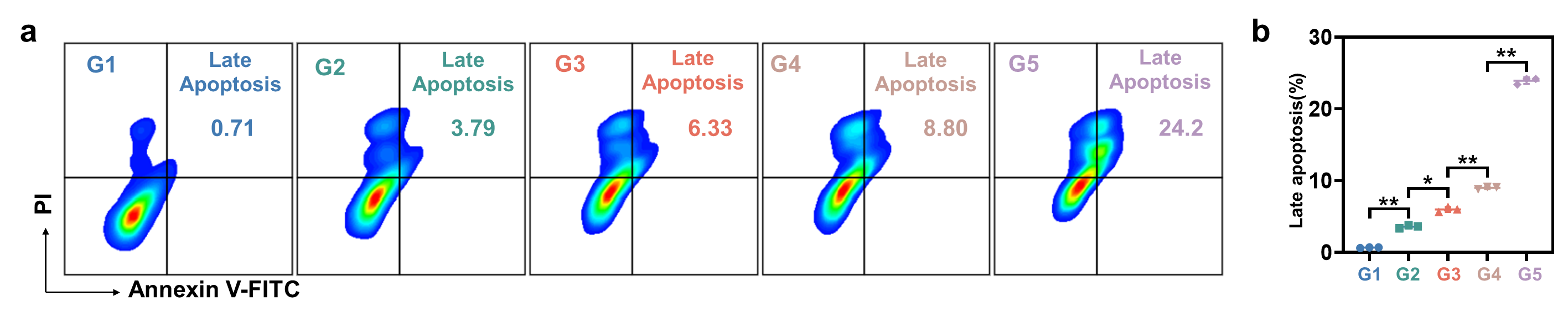
**

**Figure S9.** (a) Representative flow cytometry dot plots showing apoptosis in 4T1 cells stained with Annexin V-FITC and propidium iodide (PI) after 24 h treatment with different formulations. Early apoptotic cells (Annexin V^+^/PI^-^), late apoptotic or necrotic cells (Annexin V^+^/PI^+^), and viable cells (Annexin V^-^/PI^-^) were distinguished. (b) Quantitative analysis of total apoptotic cells (sum of early and late apoptosis) from each group. G1: PBS, G2: Mn-MOF, G3: Mn-CDDP, G4: Mn-CDDP-dBET6, G5: Mn-CDDP-dBET6@CM. Data presented as mean ± S.D. (n=3). **P* < 0.05, ***P* < 0.01, ****P* < 0.001, *****P* < 0.0001.

**
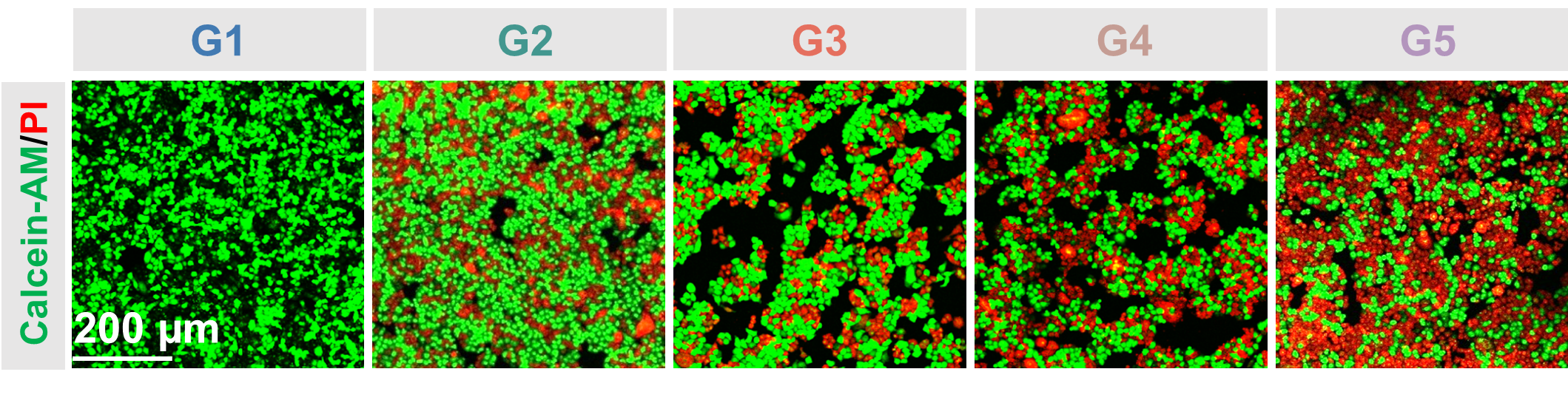
**

**Figure S10.** Fluorescence microscopy images of 4T1 cells co-stained with Calcein AM and propidium iodide (PI) after different treatments. G1: PBS, G2: Mn-MOF, G3: Mn-CDDP, G4: Mn-CDDP-dBET6, G5: Mn-CDDP-dBET6@CM.

**
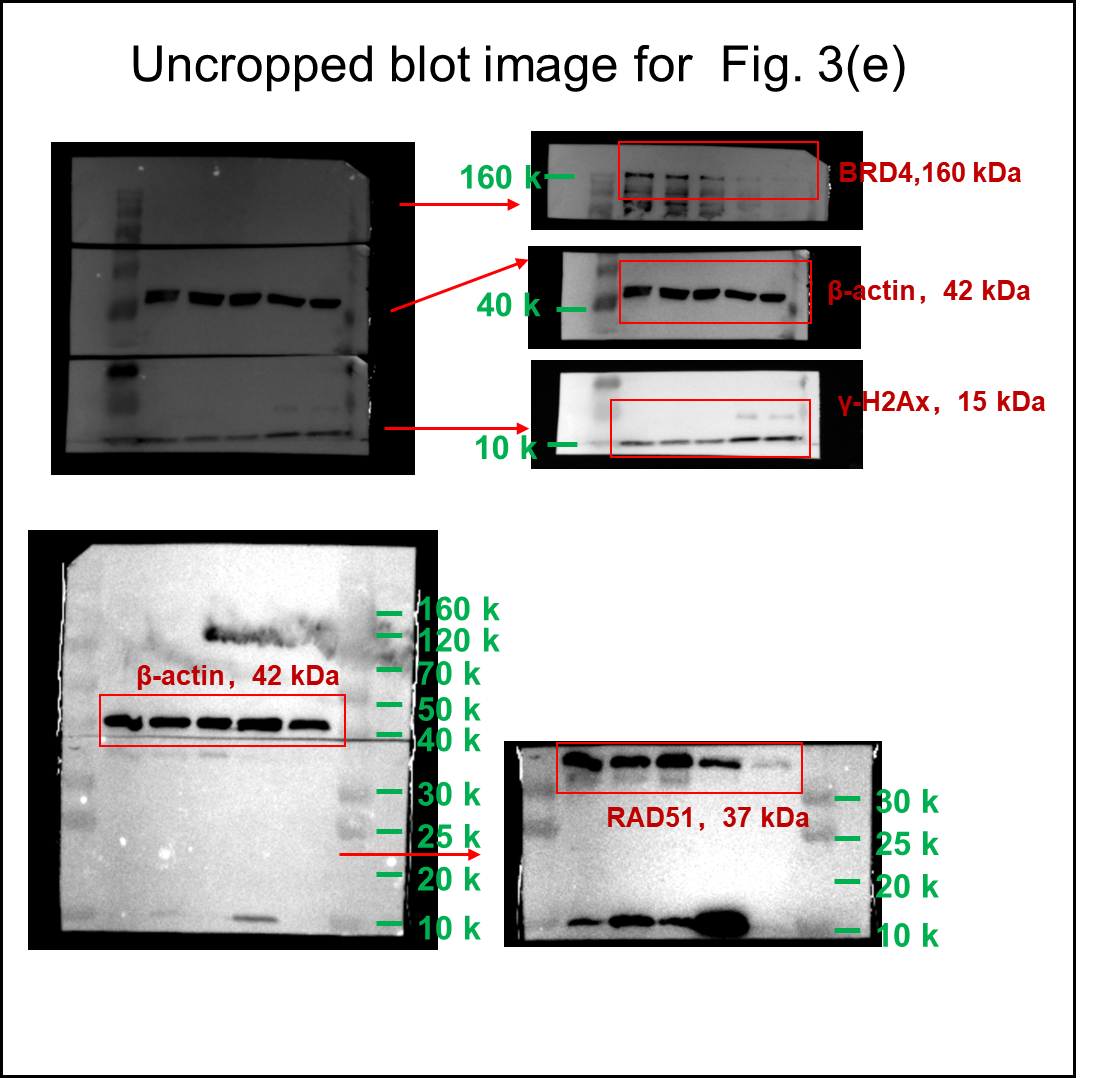
**

**Figure S11.** Uncropped blot image for Fig. 3(e).

**
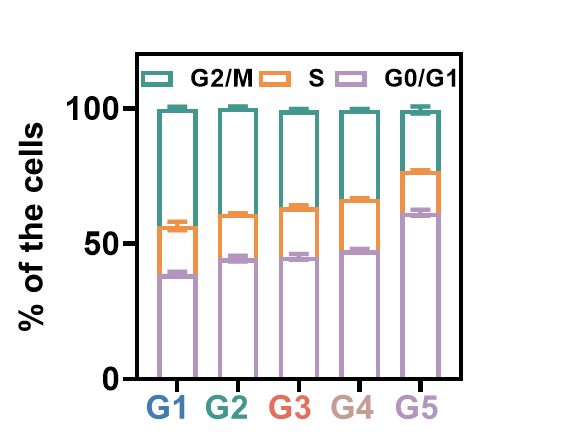
**

**Figure S12.** The corresponding quantitative analysis of cell cycle profiles by flow cytometry after propidium iodide staining after various treatments. G1: PBS, G2: Mn-MOF, G3: Mn-CDDP, G4: Mn-CDDP-dBET6, G5: Mn-CDDP-dBET6@CM. Data presented as mean ± S.D. (n=3). **P* < 0.05, ***P* < 0.01, ****P* < 0.001, *****P* < 0.0001.

**
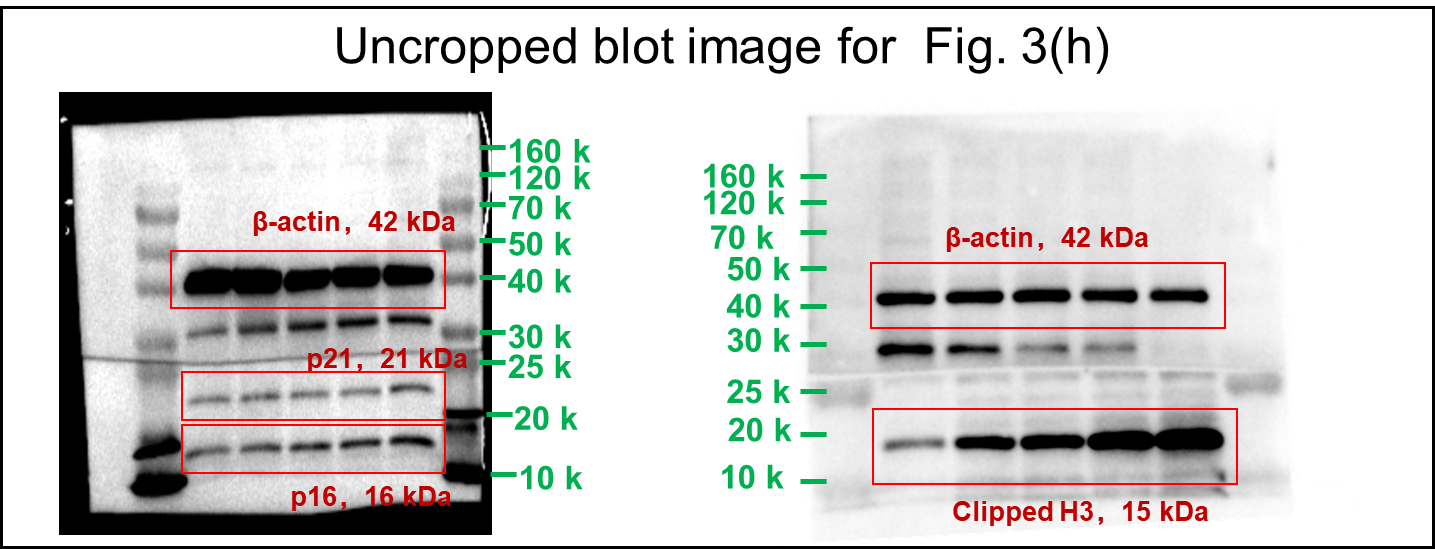
**

**Figure S13.** Uncropped blot image for Figure 3(h).

**
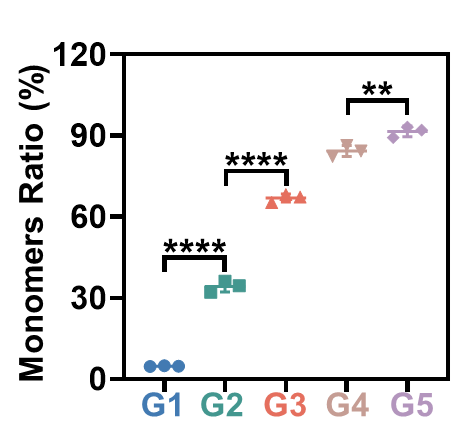
**

**Figure S14.** The corresponding quantitative analysis of representative flow cytometry patterns of 4T1 cells treated with the mitochondrial probe JC-1. G1: PBS, G2: Mn-MOF, G3: Mn-CDDP, G4: Mn-CDDP-dBET6, G5: Mn-CDDP-dBET6@CM. Data presented as mean ± S.D. (n=3). **P* < 0.05, ***P* < 0.01, ****P* < 0.001, *****P* < 0.0001.

**
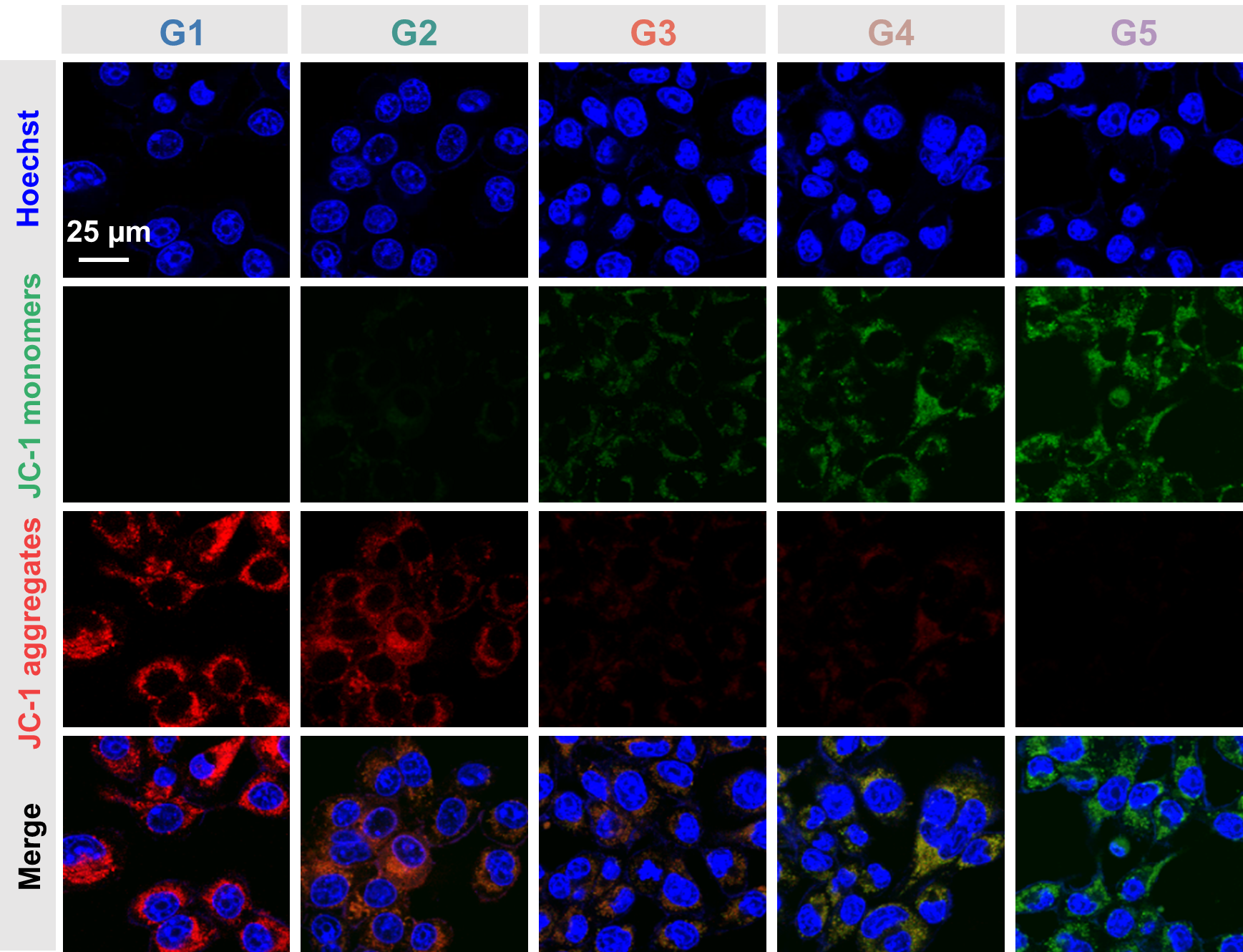
**

**Figure S15.** CLSM images of JC-1-stained 4T1 cells after different treatments for 24 h. G1: PBS, G2: Mn-MOF, G3: Mn-CDDP, G4: Mn-CDDP-dBET6, G5: Mn-CDDP-dBET6@CM.

**
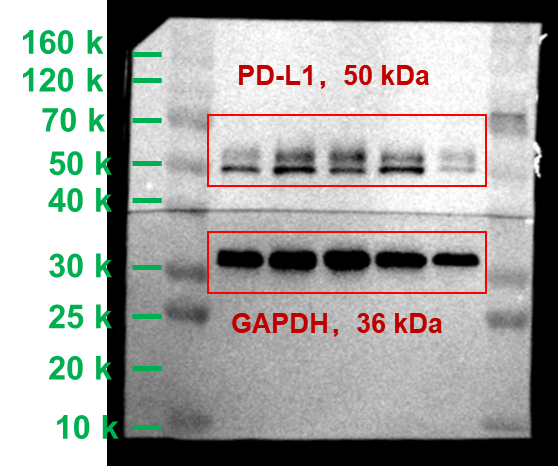
**

**Figure S16.** Uncropped blot image for Figure 4(d).

**
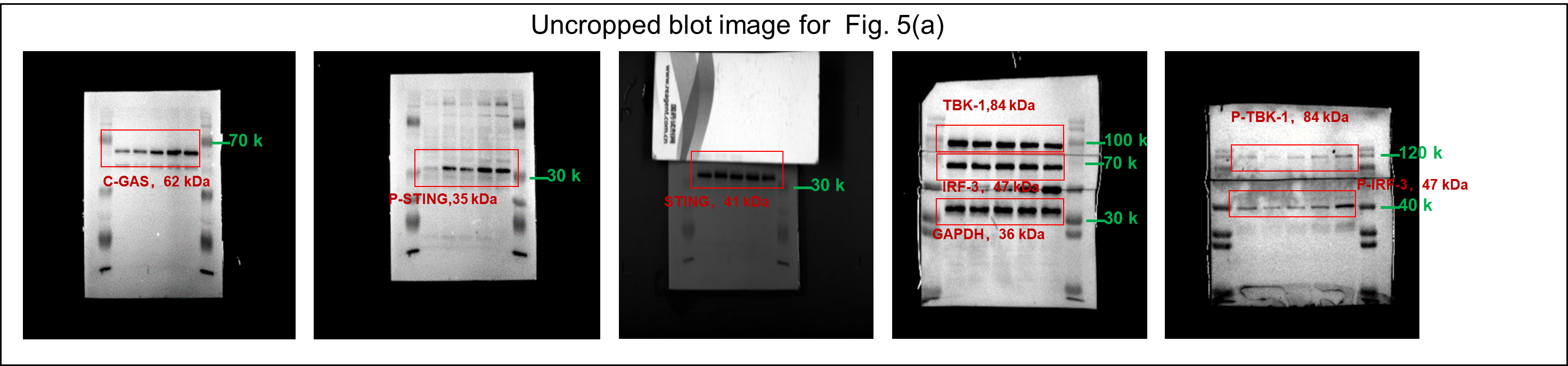
**

**Figure S17.** Uncropped blot image for Figure 5(a).

**
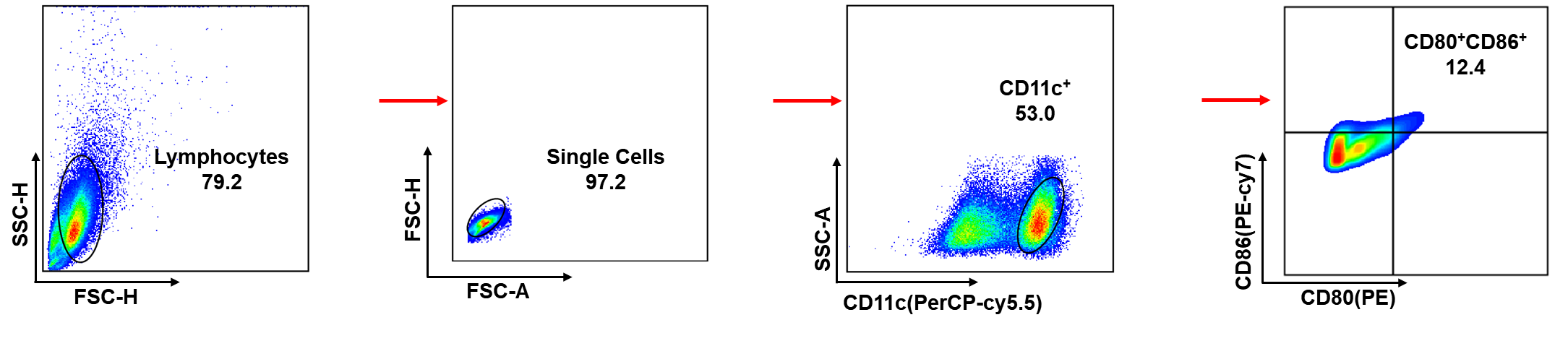
**

**Figure S18.** Gating strategy for flow cytometry analysis of mature DCs (CD11c^+^ CD80^+^ CD86^+^) in the tumors and lymph nodes.

**
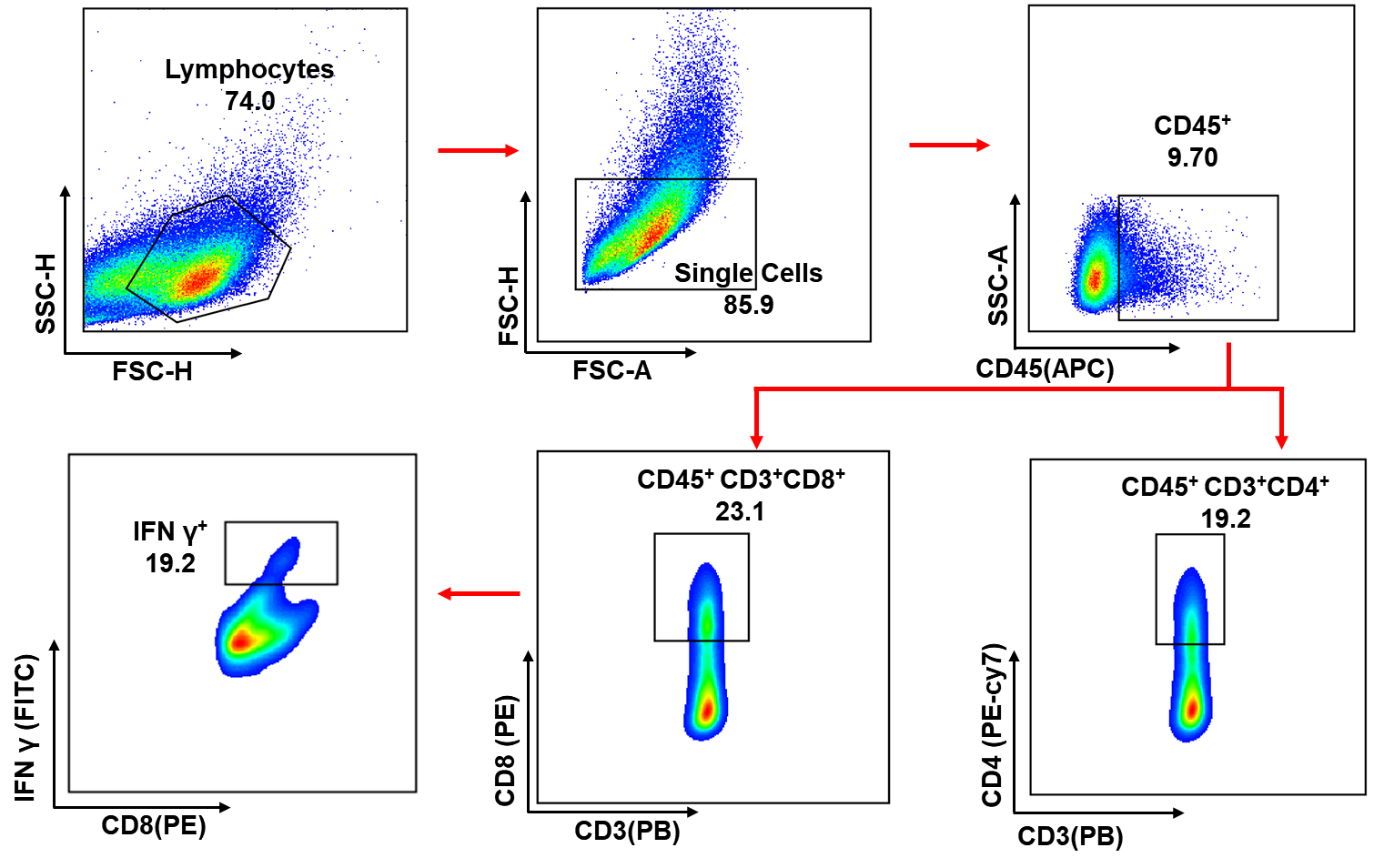
**

**Figure S19.** Gating strategy for flow cytometry analysis of CD4^+^ T cells (CD45^+^ CD3^+^ CD4^+^), CD8^+^ T cells (CD45^+^ CD3^+^ CD8^+^) in the tumors and lymph nodes. IFN γ^+^ T cells (CD3^+^ CD8^+^ IFN γ^+^) T cells in the lymph nodes.

**
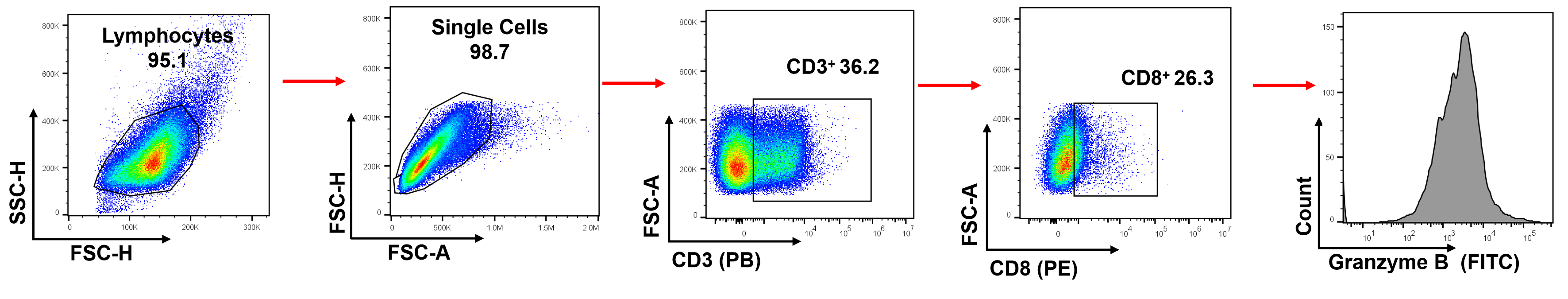
**

**Figure S20.** Gating strategy for flow cytometry analysis of Granzyme B^+^ T cells (CD3^+^ CD8^+^ Granzyme B^+^) in the co-culture system of tumor cells and immune cells.


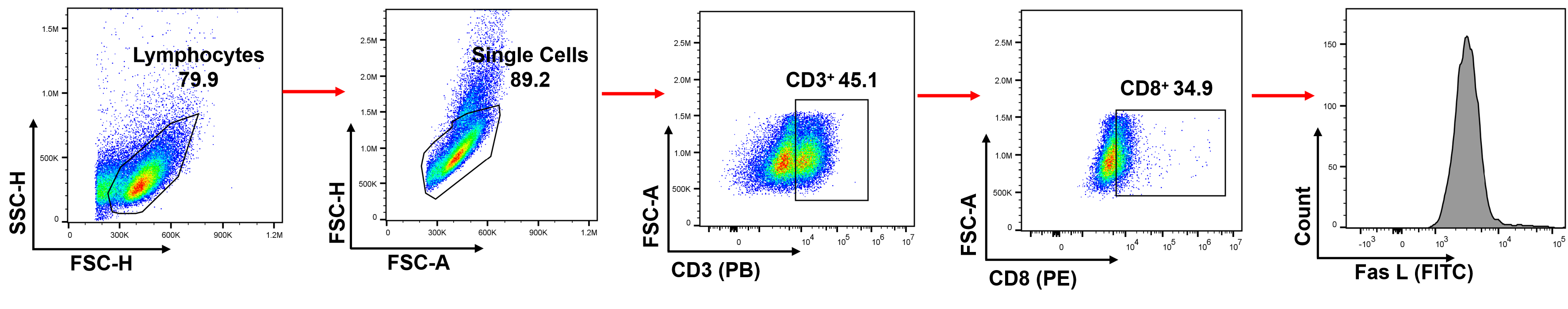
**Figure S21.** Gating strategy for flow cytometry analysis of Fas L^+^ T cells (CD3^+^ CD8^+^ Fas L^+^) in the co-culture system of tumor cells and immune cells.
**
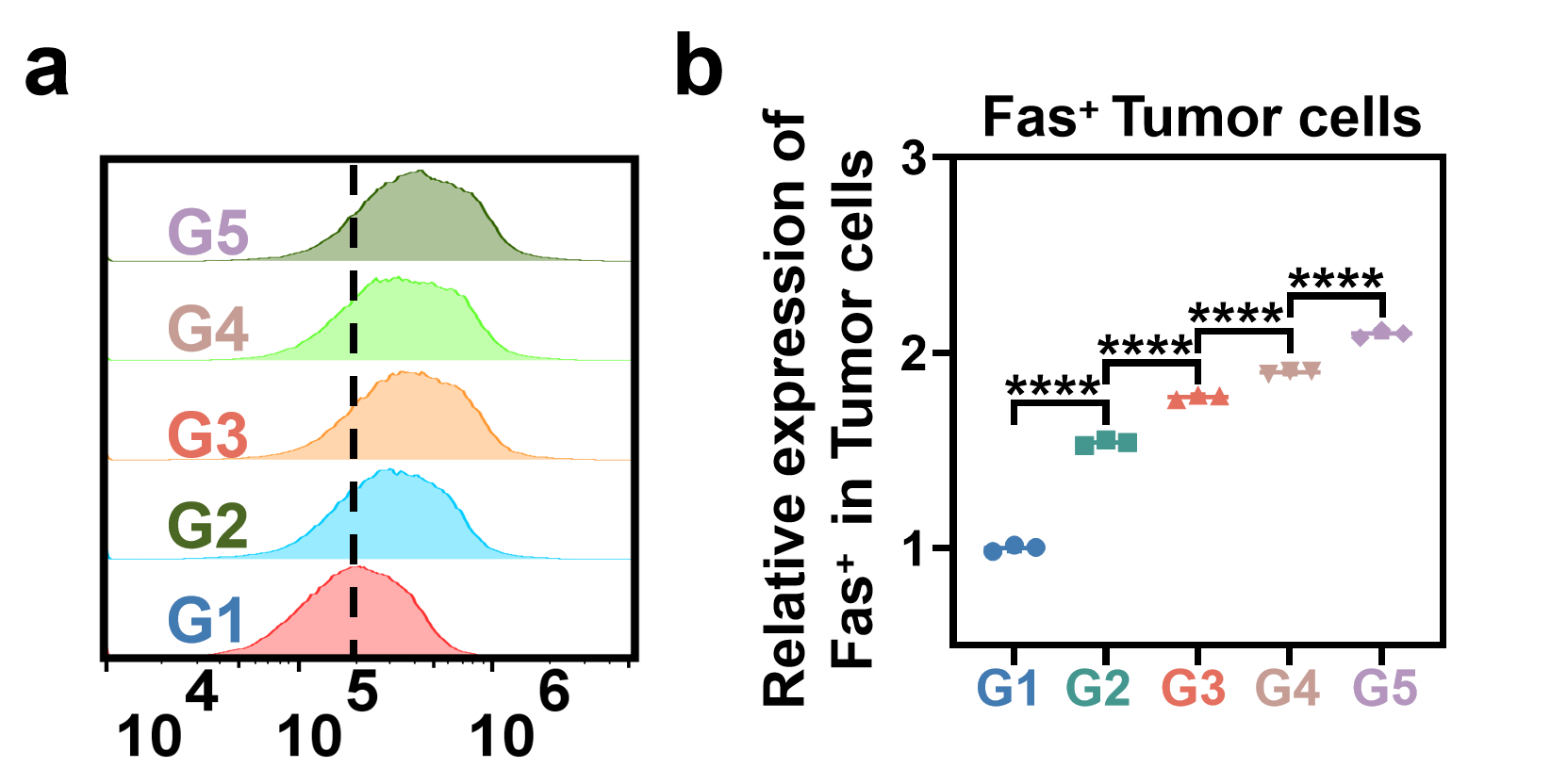
**

**Figure S22.** (a) Representative flow cytometry plots showing Fas receptor expression on tumor cells and (b) quantitative analysis of the mean MFI of Fas on tumor cells. **P* < 0.05, ***P* < 0.01, ****P* < 0.001, *****P* < 0.0001.

**
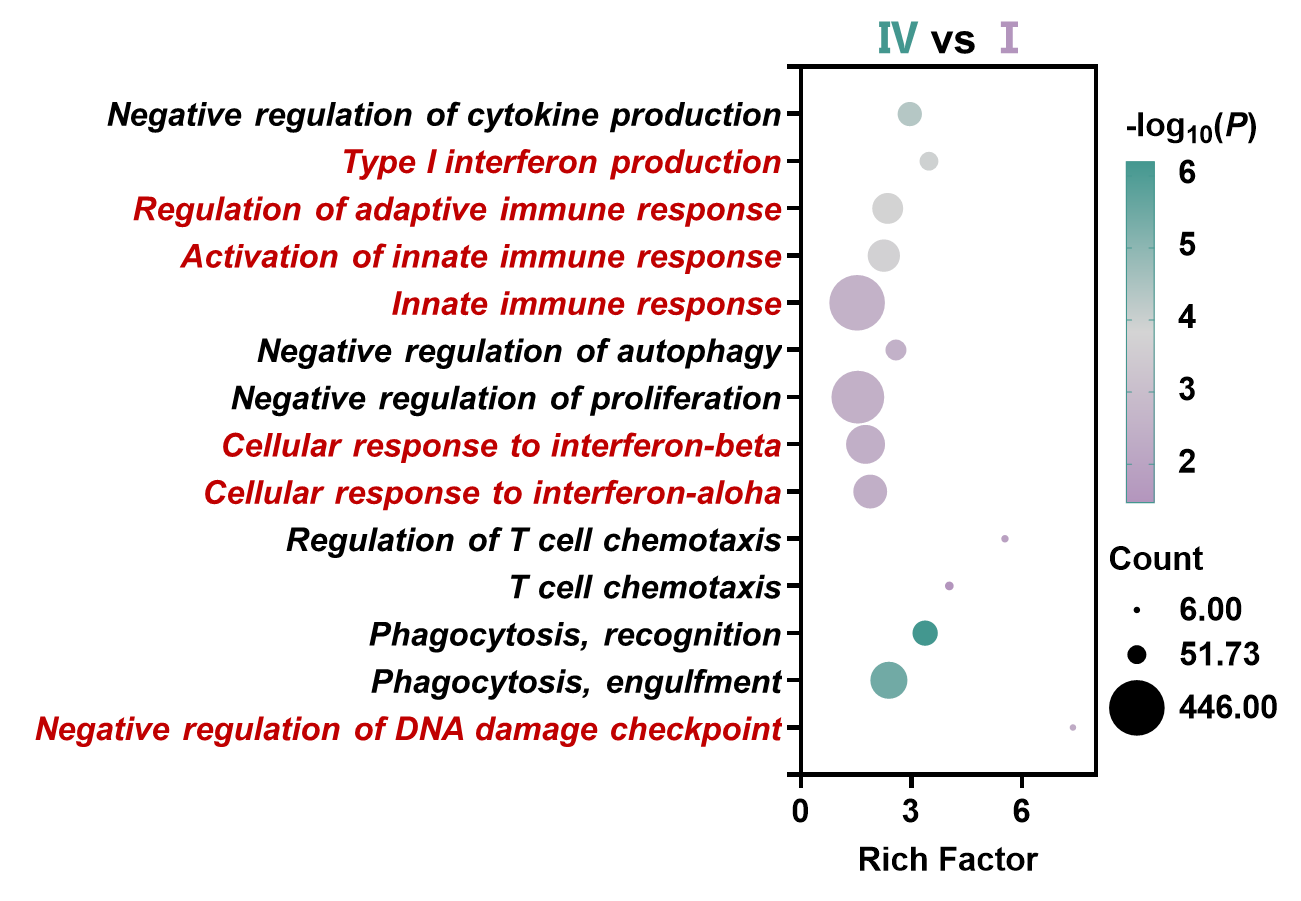
**

**Figure S23.** Gene Ontology (GO) biological process enrichment analysis of differentially expressed genes (DEGs) between PBS-treated and Mn-CDDP-dBET6@CM-treated 4T1 cells. Ⅰ: PBS, Ⅱ: Mn-MOF@CM, Ⅲ: Mn-CDDP@CM, Ⅳ: Mn-CDDP-dBET6@CM.

**
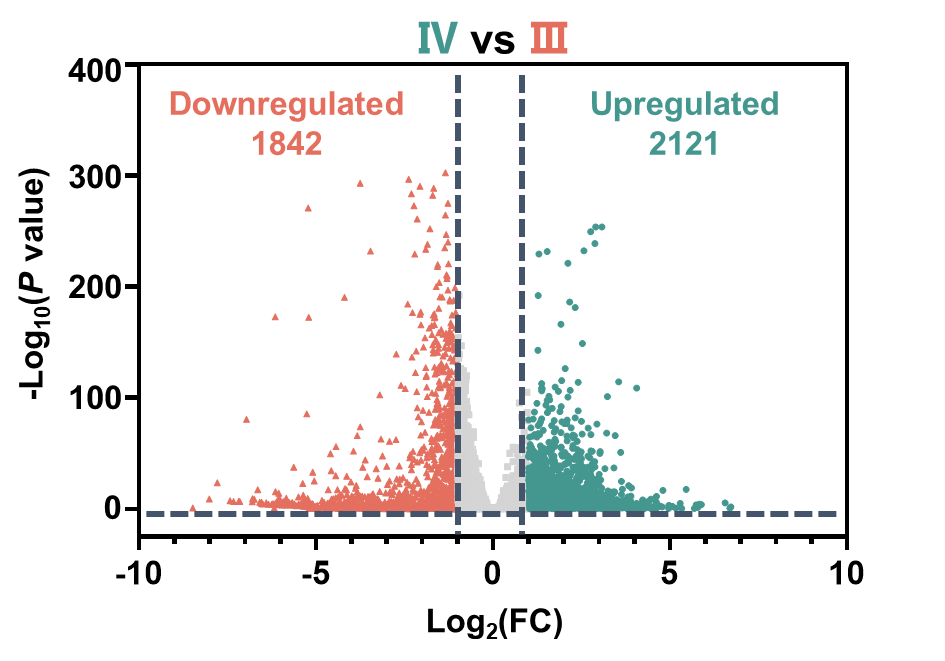
**

**Figure S24.** Volcano plot illustrating differentially expressed genes (DEGs) between Mn-CDDP@CM- and Mn-CDDP-dBET6@CM-treated 4T1 cells. Ⅰ: PBS, Ⅱ: Mn-MOF@CM, Ⅲ: Mn-CDDP@CM, Ⅳ: Mn-CDDP-dBET6@CM.

**
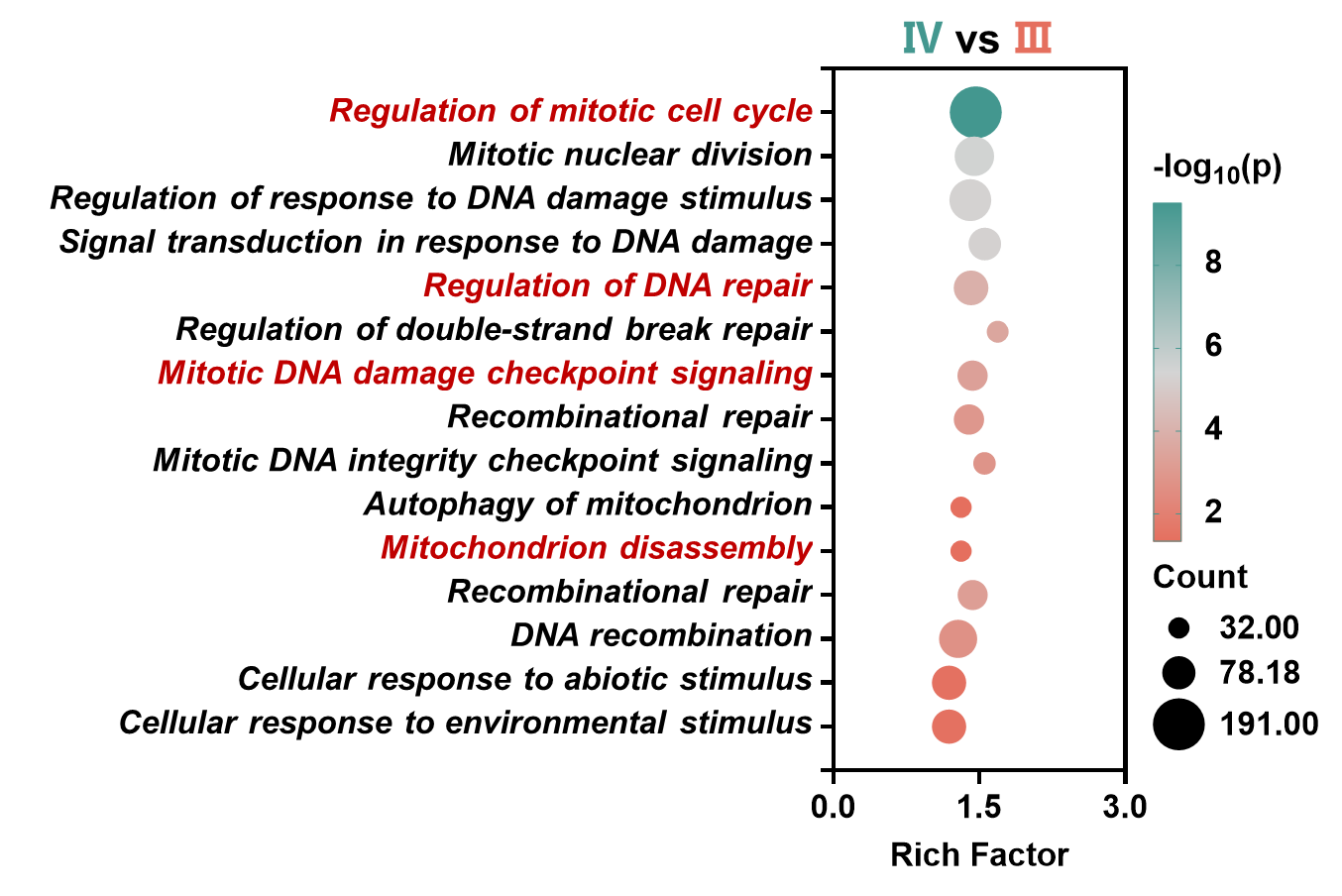
**

**Figure S25.** Gene Ontology (GO) biological process enrichment analysis of differentially expressed genes (DEGs) between Mn-CDDP@CM- and Mn-CDDP-dBET6@CM-treated 4T1 cells. Ⅰ: PBS, Ⅱ: Mn-MOF@CM, Ⅲ: Mn-CDDP@CM, Ⅳ: Mn-CDDP-dBET6@CM.

**
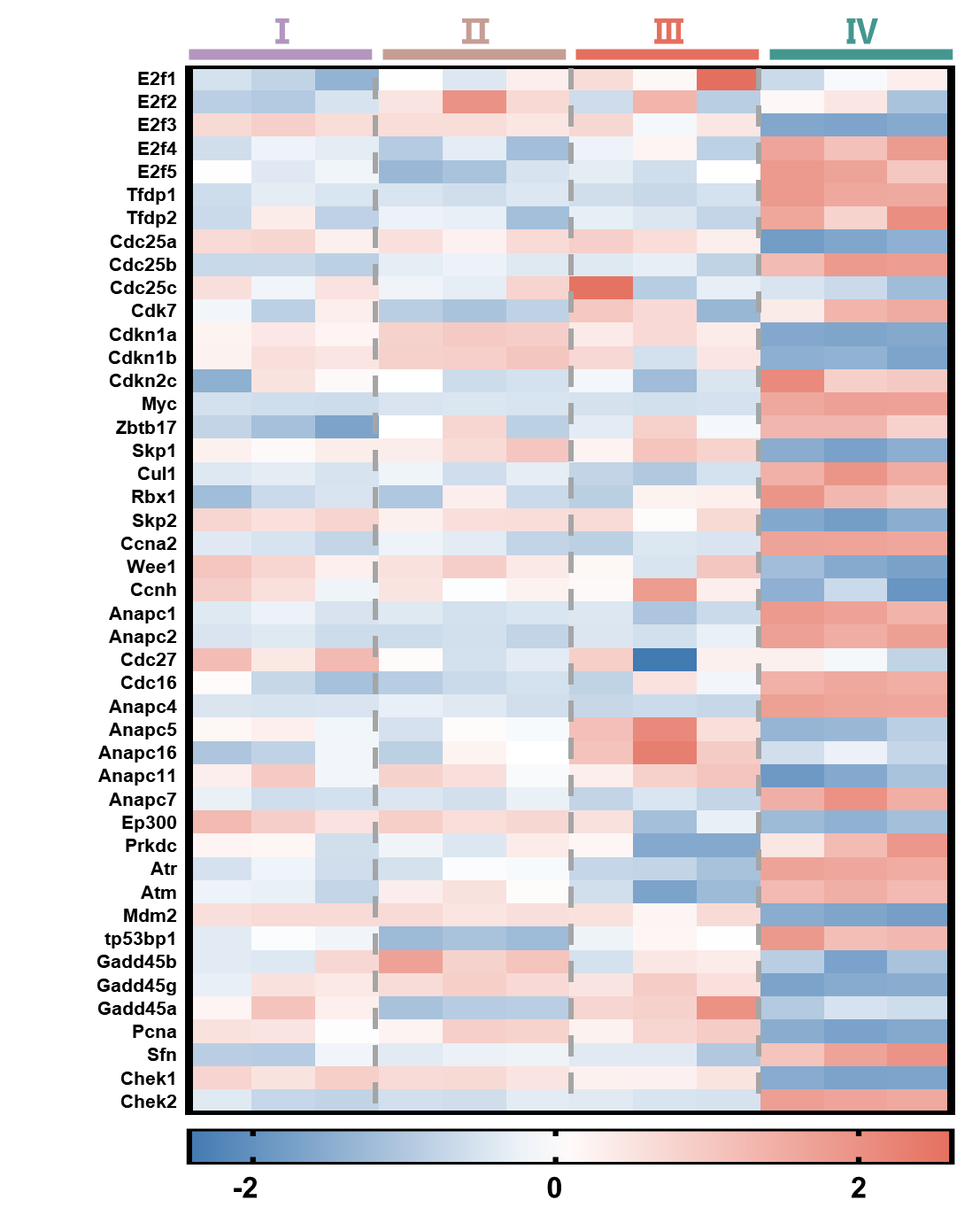
**

**Figure S26.** Heat map of cell cycle-related gene expression in 4T1 cells treated with PBS, Mn-MOF@CM, Mn-CDDP@CM, and Mn-CDDP-dBET6@CM. Ⅰ: PBS, Ⅱ: Mn-MOF@CM, Ⅲ: Mn-CDDP@CM, Ⅳ: Mn-CDDP-dBET6@CM.

**
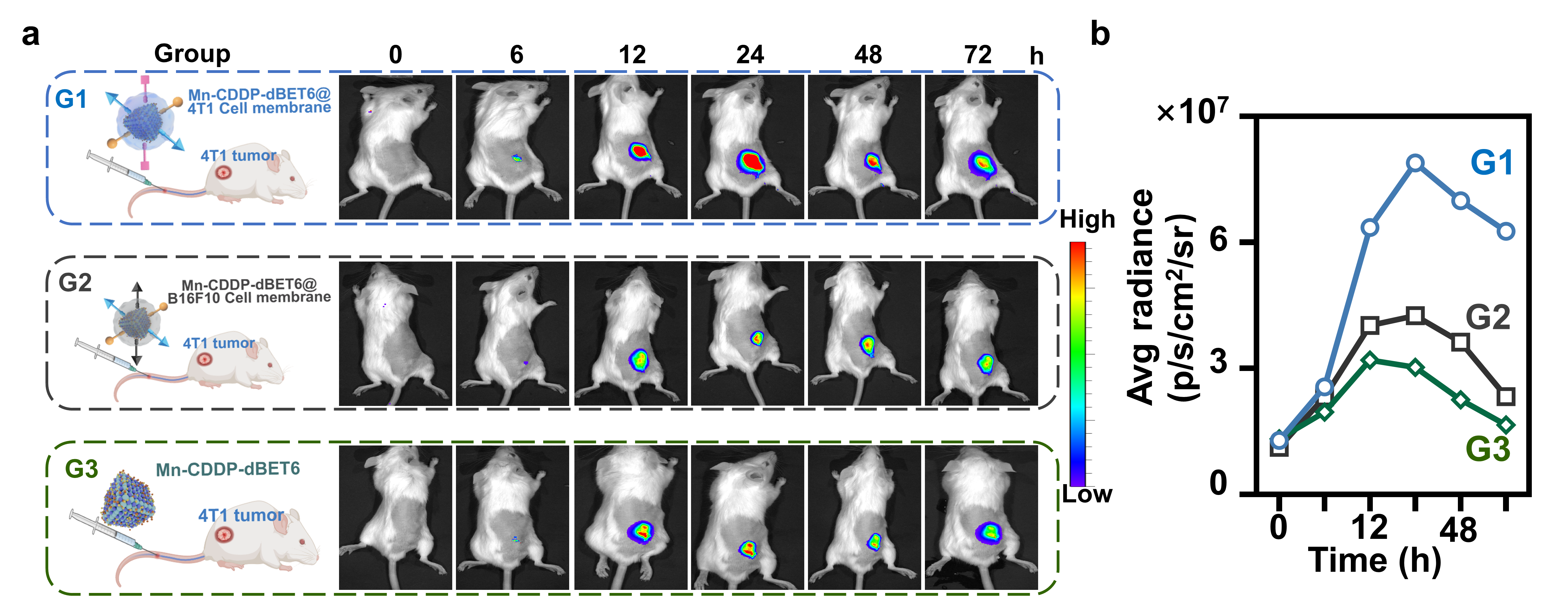
**

**Figure S27.** *In vivo* biodistribution of different nanoparticle formulations in 4T1-luc tumor-bearing mice. (a) Representative *in vivo* bioluminescence and fluorescence imaging of 4T1-luc tumor-bearing mice at various time points following intravenous injection of Cy5-labeled nanoparticles: Mn-CDDP-dBET6@4T1 CM (homologous membrane-coated), Mn-CDDP-dBET6@B16F10 CM (heterologous membrane-coated), and Mn-CDDP-dBET6 (non-coated). Fluorescence imaging was used to monitor the accumulation and retention of each formulation in the tumor region over time. (b) Quantitative analysis of mean fluorescence intensity (MFI) in the tumor region, showing enhanced tumor targeting and prolonged retention for the homologous membrane-coated Mn-CDDP-dBET6@4T1 CM group.

**
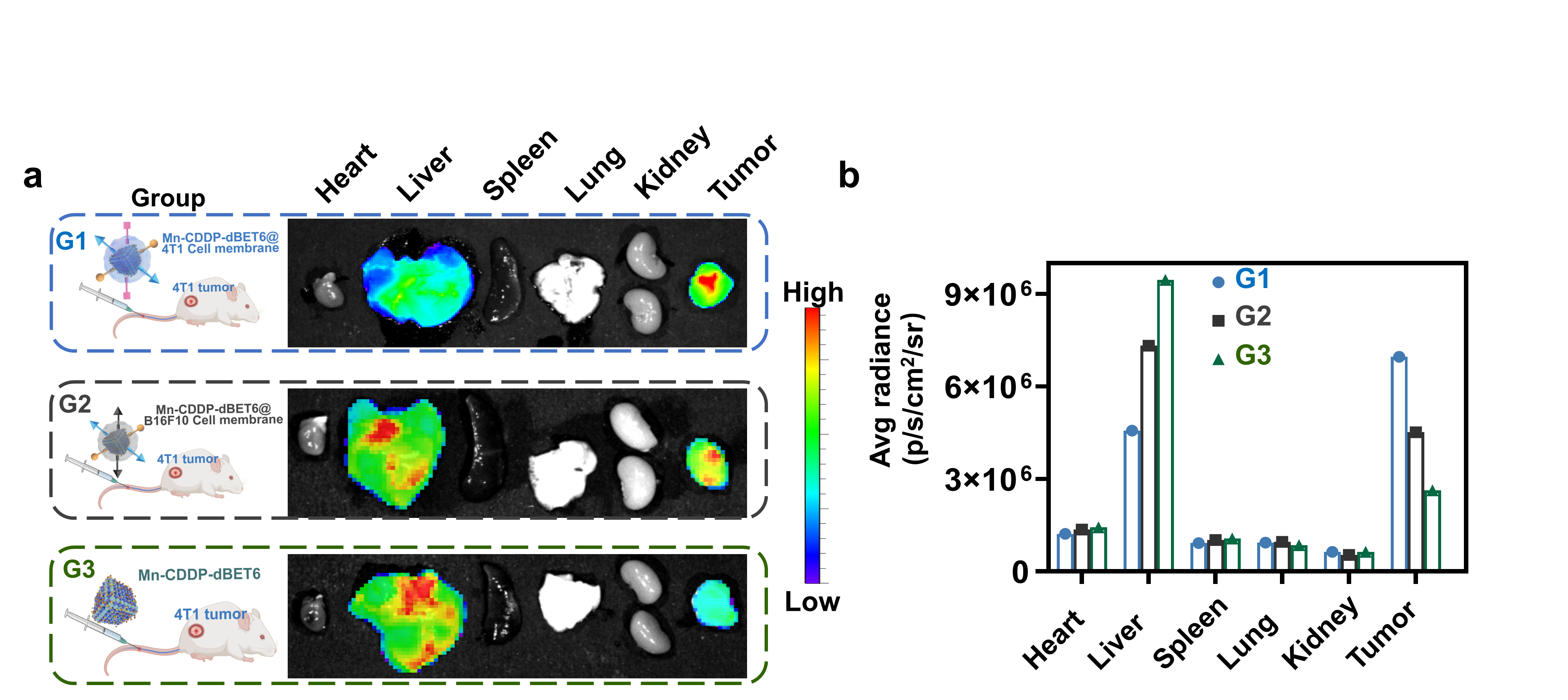
**

**Figure S28.** *Ex vivo* biodistribution analysis of Cy5-labeled Mn‑CDDP‑dBET6@CM in 4T1 tumor-bearing mice. (a) Representative *ex vivo* fluorescence images of tumors and major organs (heart, liver, spleen, lungs, kidneys, and tumor) collected 72 hours after intravenous injection of Cy5-labeled Mn‑CDDP‑dBET6@CM. Fluorescence intensity reflects the accumulation and retention of nanoparticles in each tissue. (b) Quantitative analysis of mean fluorescence intensity (MFI) in the excised organs, demonstrating preferential accumulation in the tumor tissue compared to non-target organs in homologous membrane-coated group.

**
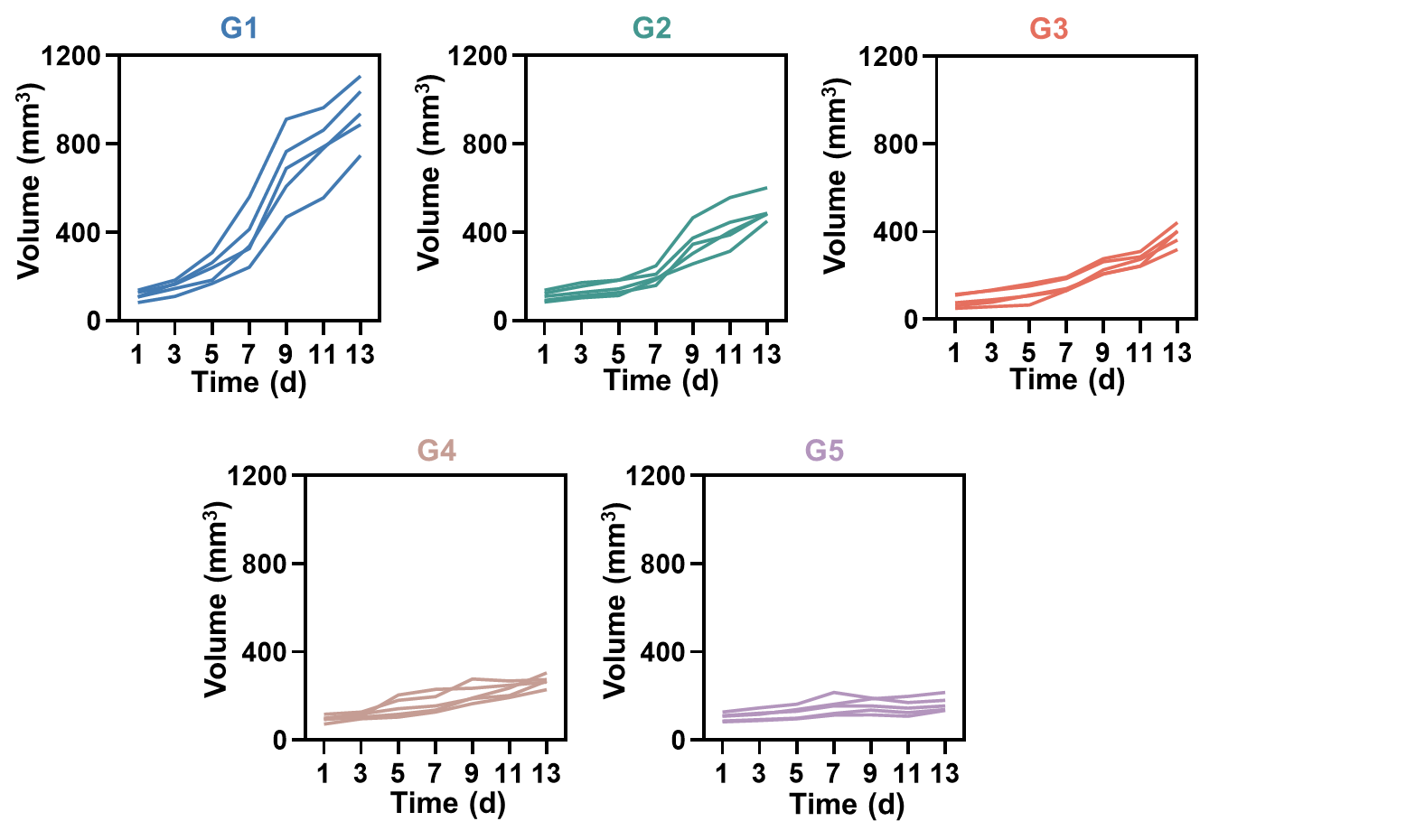
**

**Figure S29.** Individual tumor growth curves of 4T1 tumor-bearing mice after different hydrogel treatment (n = 5). G1: PBS, G2: Mn-MOF, G3: Mn-CDDP, G4: Mn-CDDP-dBET6, G5: Mn-CDDP-dBET6@CM.

**
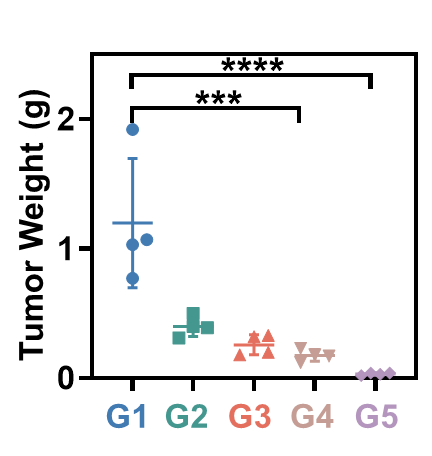
**

**Figure S30.** Mice’s Tumor weight changes after different treatments. (n = 4). G1: PBS, G2: Mn-MOF, G3: Mn-CDDP, G4: Mn-CDDP-dBET6, G5: Mn-CDDP-dBET6@CM. Data presented as mean ± S.D. (n=4). **P* < 0.05, ***P* < 0.01, ****P* < 0.001, *****P* < 0.0001.

**
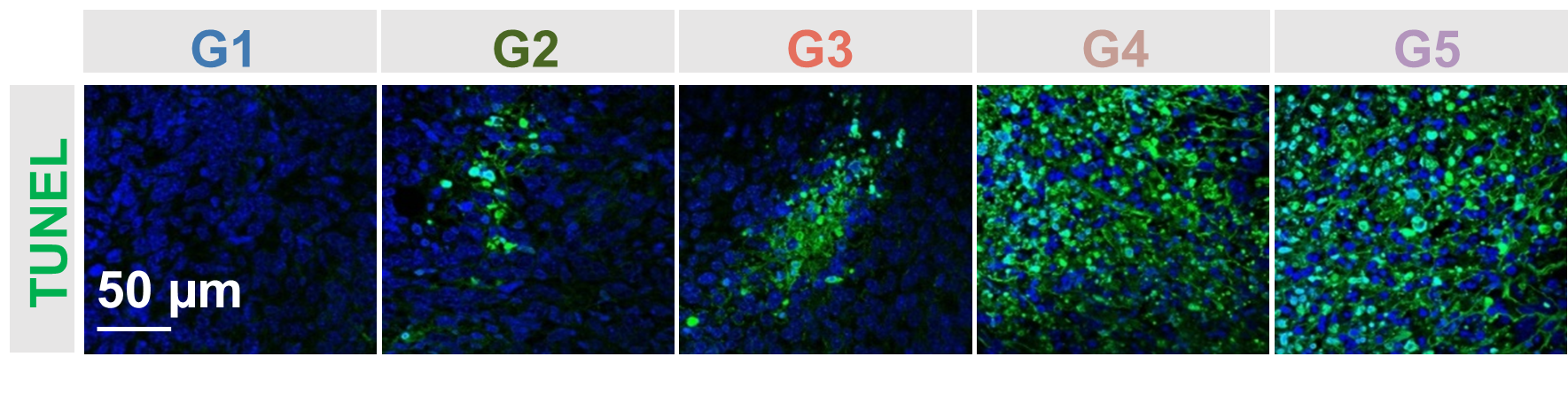
**

**Figure S31.** TUNEL staining of tumor tissues treated with different nanoplatform. G1: PBS, G2: Mn-MOF, G3: Mn-CDDP, G4: Mn-CDDP-dBET6, G5: Mn-CDDP-dBET6@CM.

**
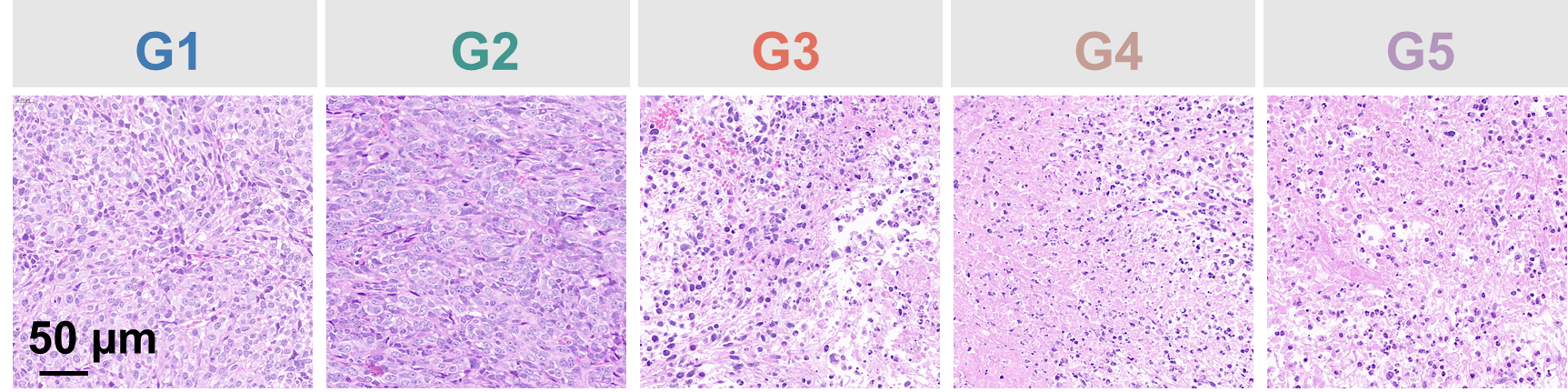
**

**Figure S32.** H&E staining of tumor tissues treated with different nanoplatform. G1: PBS, G2: Mn-MOF, G3: Mn-CDDP, G4: Mn-CDDP-dBET6, G5: Mn-CDDP-dBET6@CM.

**
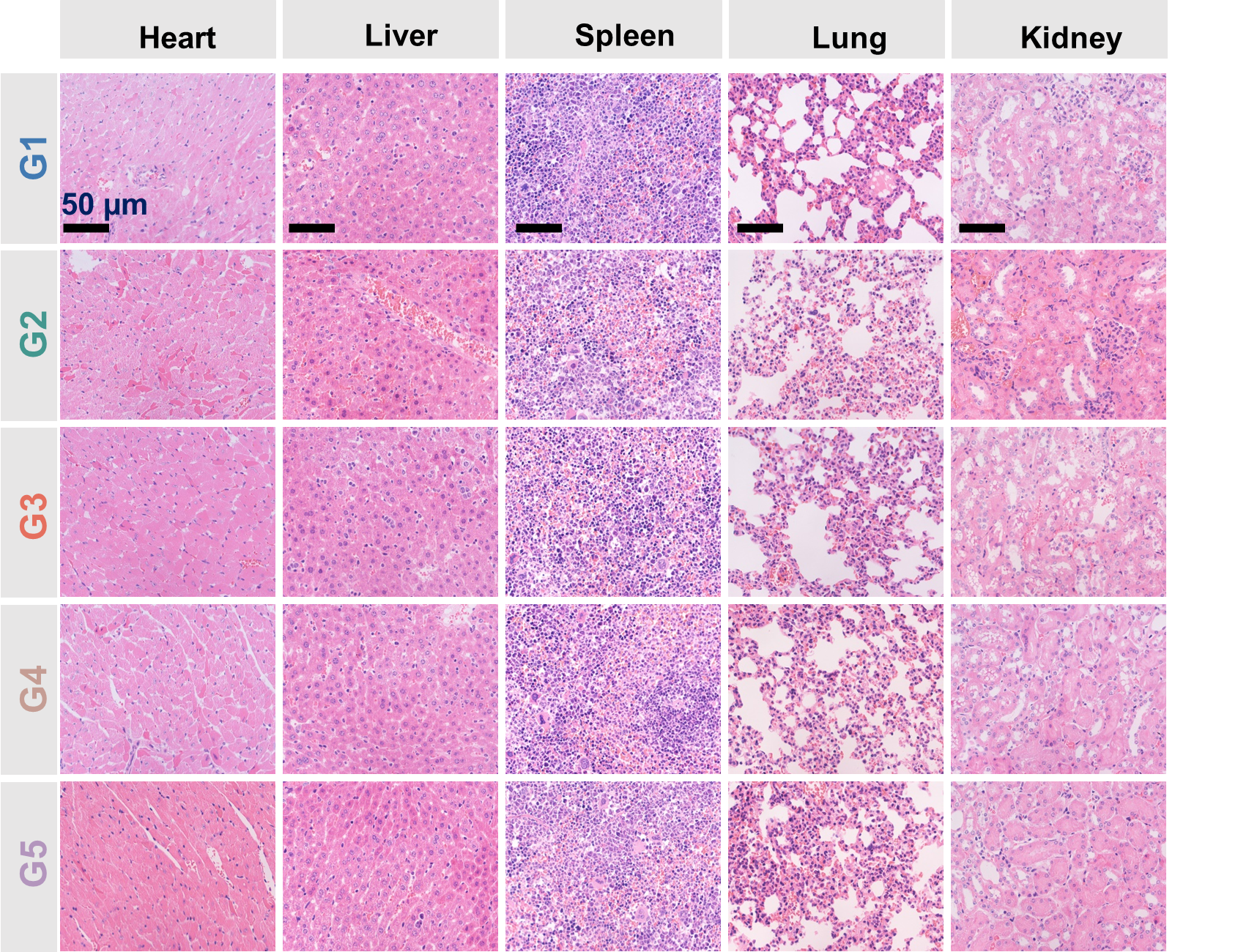
**

**Figure S33.** H&E staining images of major organs (heart, liver, spleen, lung and kidney) after various treatments. G1: PBS, G2: Mn-MOF, G3: Mn-CDDP, G4: Mn-CDDP-dBET6, G5: Mn-CDDP-dBET6@CM.

**
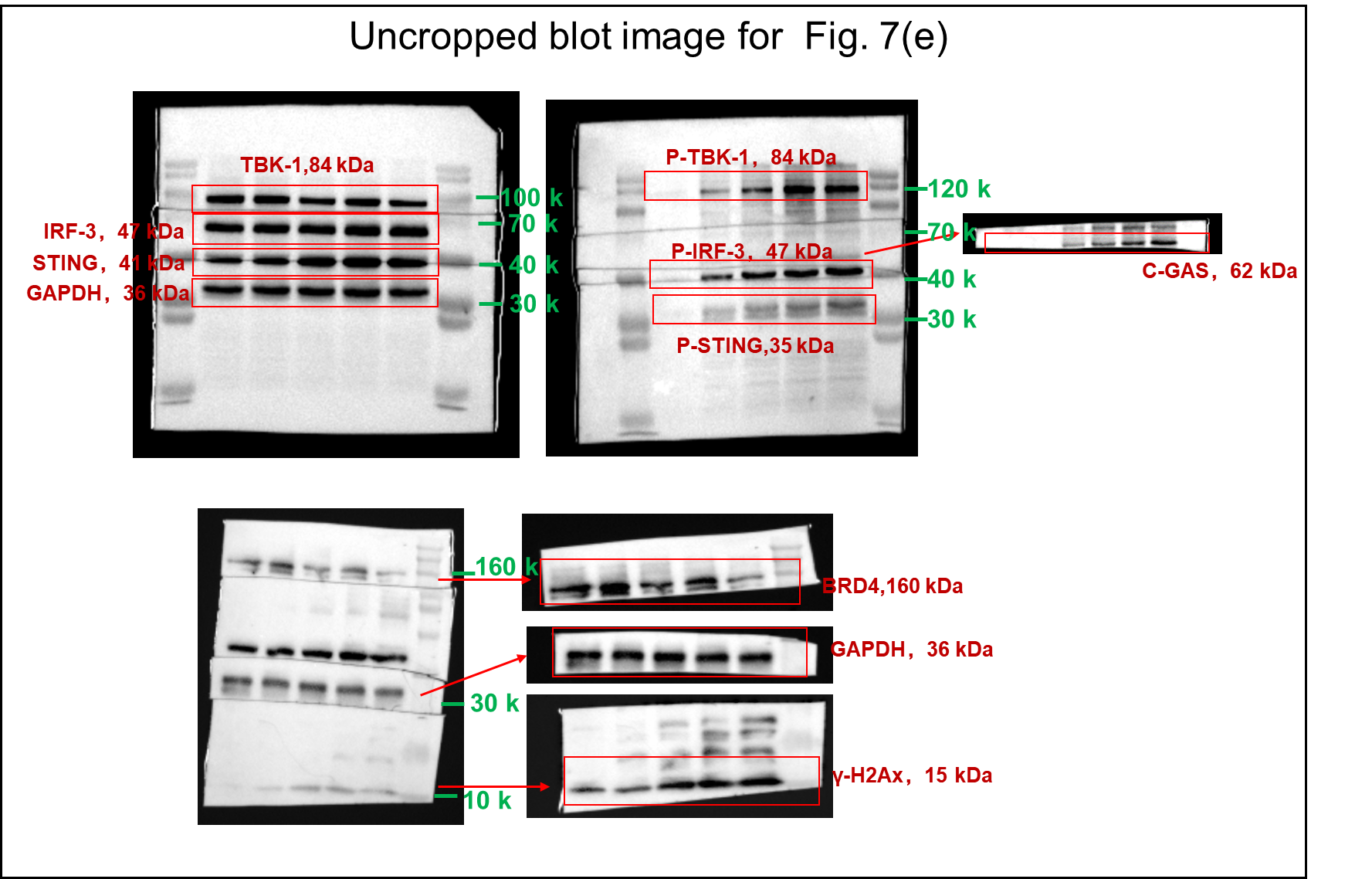
**

**Figure S34.** Uncropped blot image for Figure 7(e).

**
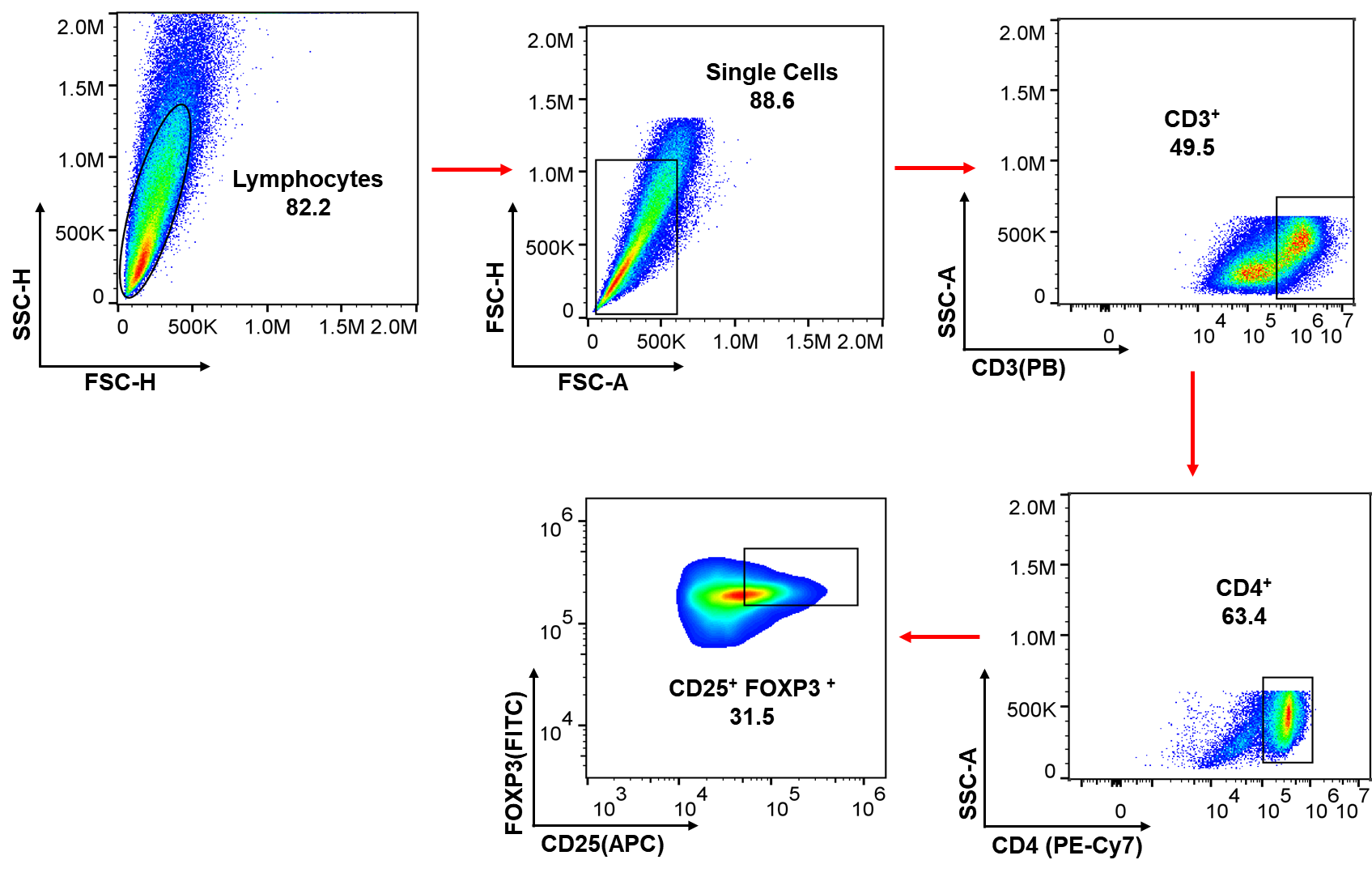
**

**Figure S35.** Gating strategy for flow cytometry analysis of CD25^+^ FOXP3^+^ Treg cells in tumors.

**
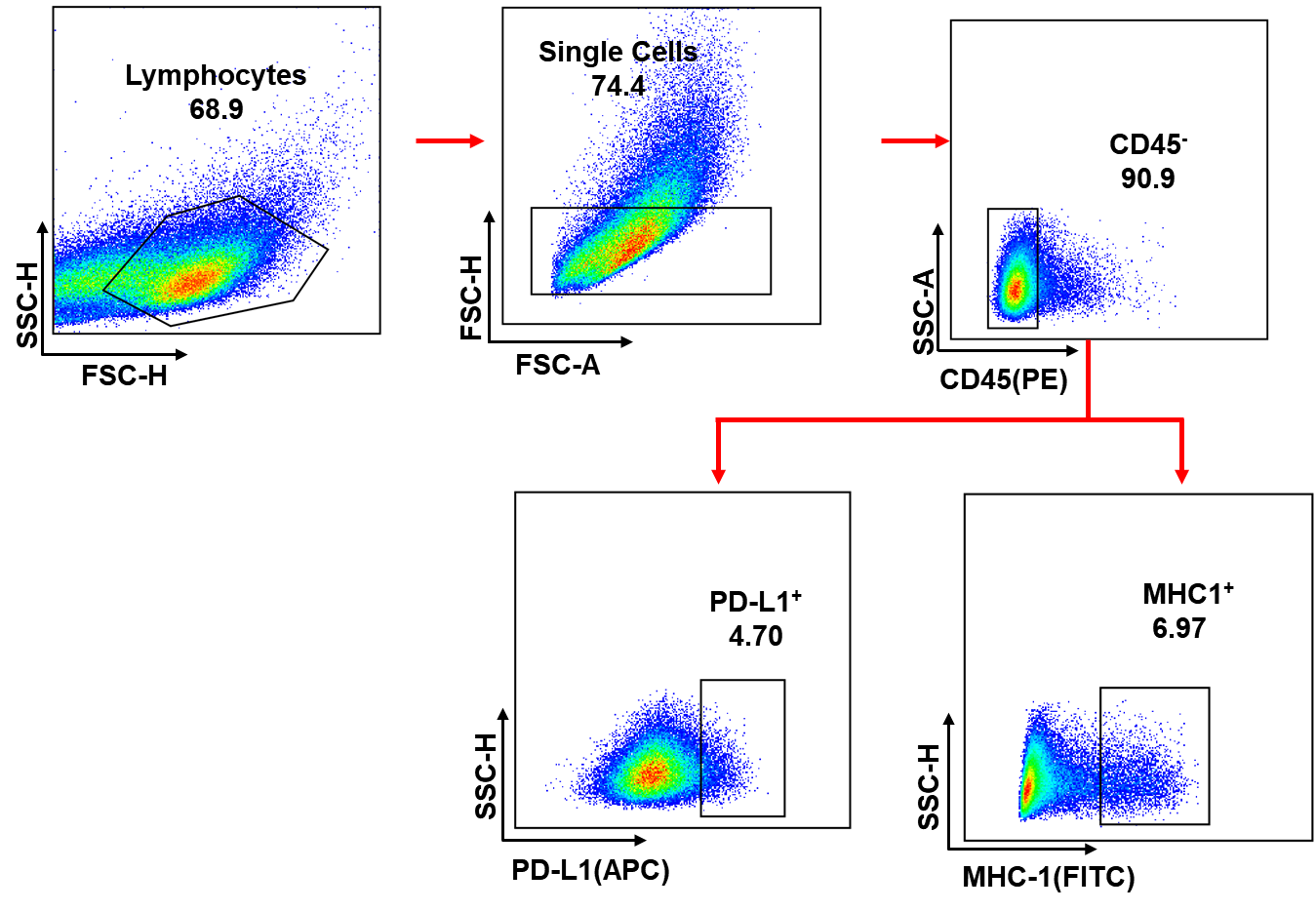
**

**Figure S36.** Gating strategy for flow cytometry analysis of MHC-I expression (CD45^-^ MHC-I^+^) and PD-L1 expression (CD45^-^ PD-L1^+^) in tumors.

**
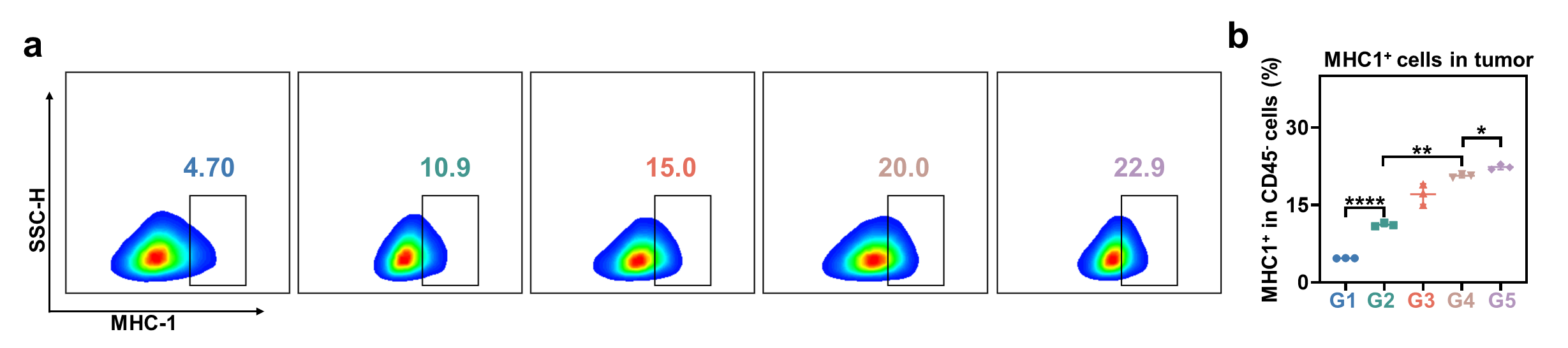
**

**Figure S37.** Representative flow cytometry patterns of MHC-1 (a) levels in tumors subjected to different treatments and corresponding quantitative analysis (b). G1: PBS, G2: Mn-MOF, G3: Mn-CDDP, G4: Mn-CDDP-dBET6, G5: Mn-CDDP-dBET6@CM. **P* < 0.05, ***P* < 0.01, ****P* < 0.001, *****P* < 0.0001.

**
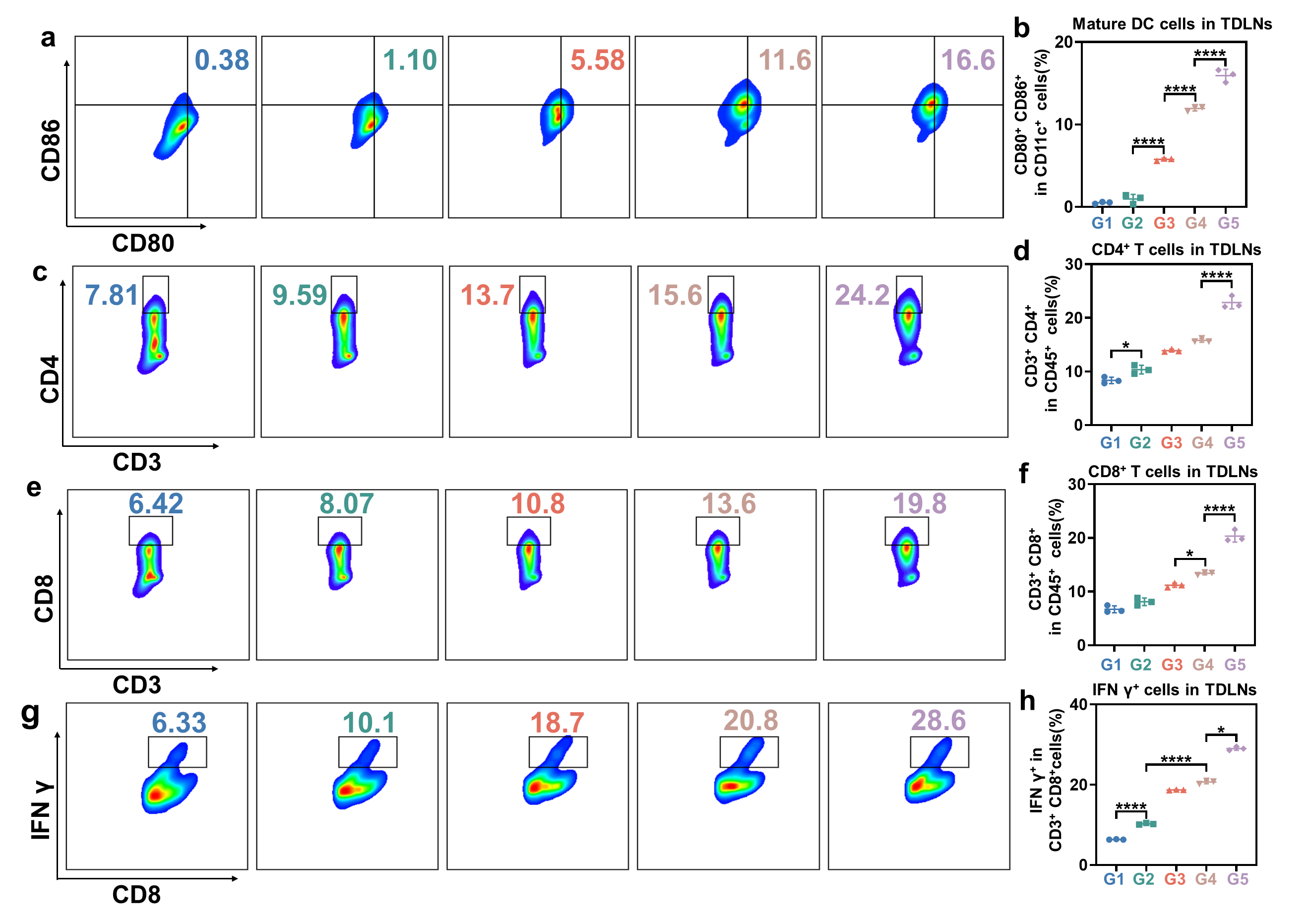
**

**Figure S38.** Immunoassays of mice with different treatments on day 21. Representative flow cytometry patterns of DC maturation levels in the lymph nodes subjected to different treatments (a) and corresponding quantitative analysis (b). Flow cytometry analysis of CD3^+^ CD4^+^ T cells in the lymph nodes (c) and quantitative analysis (d). Flow cytometry analysis of CD3^+^ CD8^+^ T cells in the lymph nodes (e) and quantitative analysis (f). Flow cytometry analysis of CD3^+^ CD8^+^ IFN γ^+^ T cells in the lymph nodes (g) and quantitative analysis (h). G1: PBS, G2: Mn-MOF, G3: Mn-CDDP, G4: Mn-CDDP-dBET6, G5: Mn-CDDP-dBET6@CM. **P* < 0.05, ***P* < 0.01, ****P* < 0.001, *****P* < 0.0001.

**
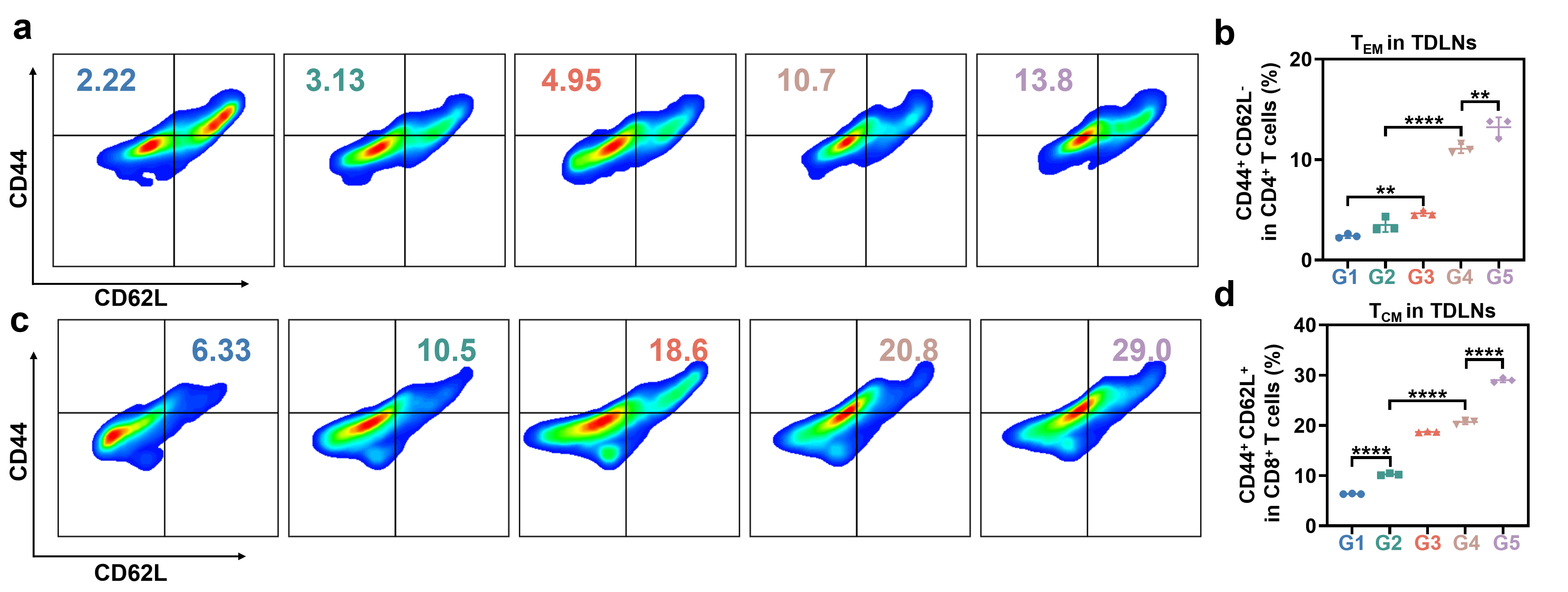
**

**Figure S39.** Memory T cells Immunoassays of mice with different treatments on day 21 (n = 3). T_EM_ (CD44^+^ CD62L^−^) among CD4^+^ T cells (a) and T_CM_ (CD44^+^ CD62L^+^) among CD8^+^ T cells (c) in the lymph nodes and quantitative analysis (b, d). G1: PBS, G2: Mn-MOF, G3: Mn-CDDP, G4: Mn-CDDP-dBET6, G5: Mn-CDDP-dBET6@CM. **P* < 0.05, ***P* < 0.01, ****P* < 0.001, *****P* < 0.0001.

**
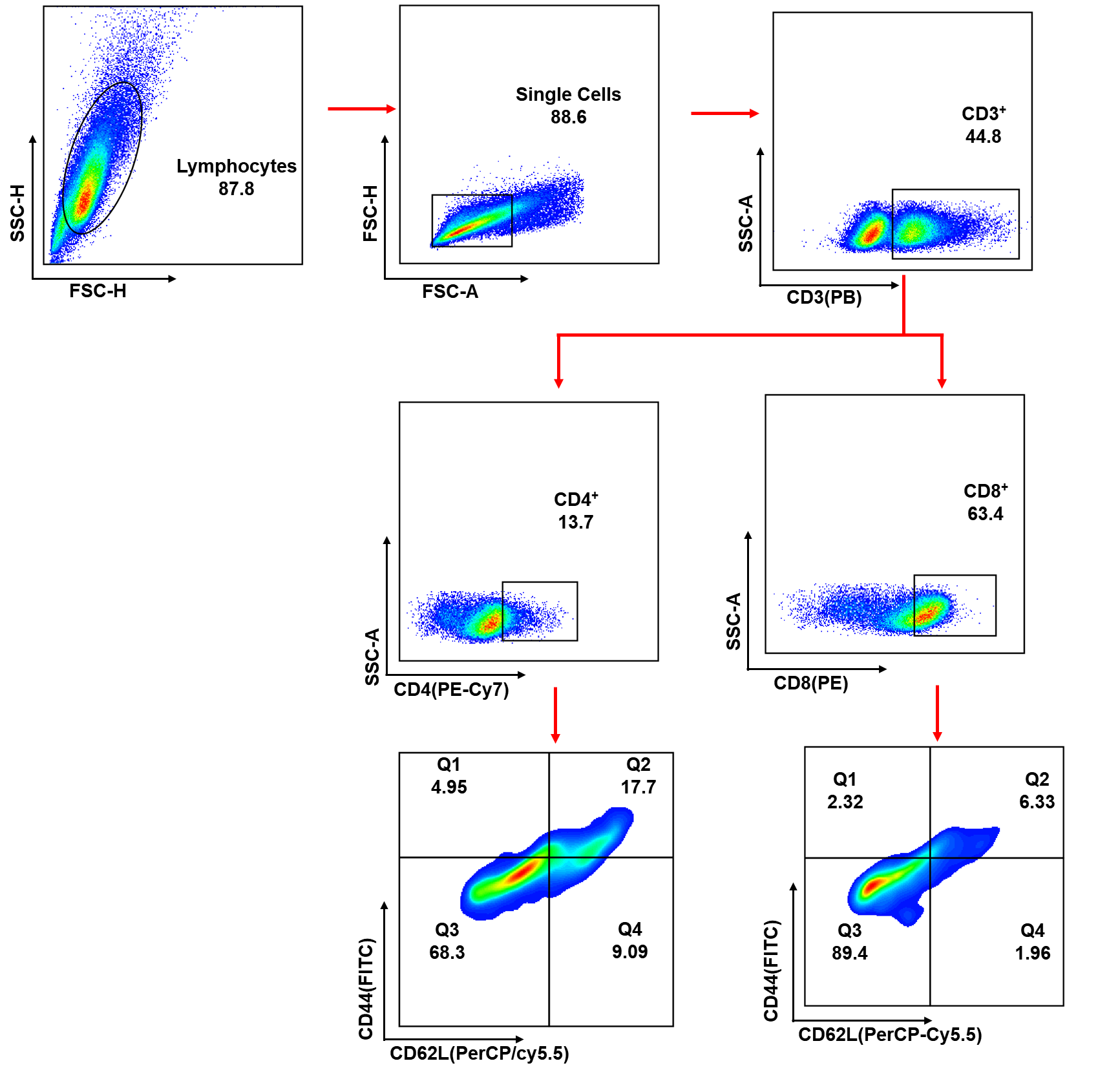
**

**Figure S40.** Gating strategy for flow cytometry analysis of T_EM_ (CD44^+^ CD62L^-^) among CD4^+^ T cells and T_CM_ (CD44^+^ CD62L^+^) among CD8^+^ T cells in the lymph nodes.
